# Investigating heterogeneity in IRTree models for multiple response processes with score‐based partitioning

**DOI:** 10.1111/bmsp.12367

**Published:** 2024-11-04

**Authors:** Rudolf Debelak, Thorsten Meiser, Alicia Gernand

**Affiliations:** ^1^ University of Zurich Zurich Switzerland; ^2^ University of Mannheim Mannheim Germany; ^3^ RPTU Kaiserslautern‐Landau Landau Germany

**Keywords:** IRTree models, item response theory, model‐based recursive partitioning, parameter heterogeneity, response styles, score‐based tests

## Abstract

Item response tree (IRTree) models form a family of psychometric models that allow researchers to control for multiple response processes, such as different sorts of response styles, in the measurement of latent traits. While IRTree models can capture quantitative individual differences in both the latent traits of interest and the use of response categories, they maintain the basic assumption that the nature and weighting of latent response processes are homogeneous across the entire population of respondents. In the present research, we therefore propose a novel approach for detecting heterogeneity in the parameters of IRTree models across subgroups that engage in different response behavior. The approach uses score‐based tests to reveal violations of parameter heterogeneity along extraneous person covariates, and it can be employed as a model‐based partitioning algorithm to identify sources of differences in the strength of trait‐based responding or other response processes. Simulation studies demonstrate generally accurate Type I error rates and sufficient power for metric, ordinal, and categorical person covariates and for different types of test statistics, with the potential to differentiate between different types of parameter heterogeneity. An empirical application illustrates the use of score‐based partitioning in the analysis of latent response processes with real data.

## 1 INTRODUCTION

Likert response formats are commonly used to assess latent traits, but it is widely known that the response categories can elicit individual response processes that affect the measurement independently of item content. Examples of such additional response processes, or response styles, include midscale and extreme responding (Baumgartner & Steenkamp, 2001; Mellenbergh, 2011), which lead to the methodological challenge of modeling effects in such a way as to disentangle the latent traits of interest from potential biases.

To account for response styles, traditional IRT models like the partial credit model (Masters, 1982) and nominal response model (Takane & De Leeuw, 1987) have been extended with additional trait dimensions (Bolt et al., 2014; Falk & Cai, 2016) or random threshold parameters (Jin & Wang, 2014; Wang & Wu, 2011) that capture individual differences in the use of response categories (for an overview, see Henninger & Meiser, 2020). An alternative approach to modeling response styles comes from item response tree (IRTree) models, that is, decision trees with IRT parameterizations of decision nodes (Böckenholt, 2012; De Boeck & Partchev, 2012). IRTree models provide a general framework for specifying multiple response processes that lead to an observed response category (Jeon & De Boeck, 2016), and they can be used to model the joint influences of substantive traits and response styles on ordinal responses (Böckenholt & Meiser, 2017; Khorramdel & von Davier, 2014; Plieninger & Meiser, 2014). Unlike partial credit and nominal response models, IRTree models do not fall into the class of divide‐by‐total models and thus allow independent specifications of the category probabilities on a Likert scale (Schoenmakers et al., 2024).

### 1.1 IRTree models for ordinal responses with response styles

**FIGURE 1:**
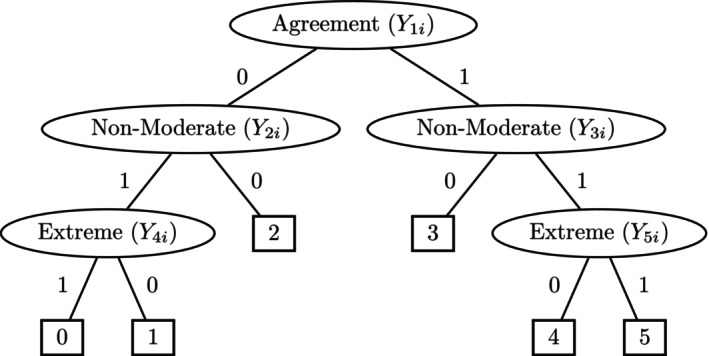
IRTree model for Likert items with a six‐point rating scale. Theoretical nodes in ellipses denote assumed decision processes, terminal nodes in squares denote observed item responses.

Conceptually, IRTree models for ordinal responses assume a sequence of logically contingent decisions, where latent traits and response styles determine the outcomes of theoretical decision nodes that terminate in an observed Likert response. Figure 1 illustrates the structure of an IRTree model for Likert items with six response categories ranging from 0 to 5 (Meiser et al., 2019; see Böckenholt, 2017, for a similar model structure). The first decision node concerns the general agreement (Categories 3, 4, 5) versus disagreement (Categories 0, 1, 2) with the item content. Conditional on the first decision, the second decision refers to the choice of a nonmoderate (i.e., .1 or 4.5) versus moderate (i.e., 2 or 3) category of (dis)agreement. Given a nonmoderate response, the third decision differentiates between extreme (i.e., 0 or 5) and nonextreme (i.e., 1 or 4) (dis)agreement.

In IRTree modeling, the decision nodes are specified as pseudo‐items with an appropriate IRT parameterization. The IRTree in Figure 1 contains five binary pseudo‐items for each Likert item i. The pseudo‐item at the first node $Y_{1i}$ distinguishes between disagreement with $Y_{1i}=0$ and agreement with $Y_{1i}=1$, and it can be parameterized, for instance, by a one‐parameter logistic function of the substantive trait θ and an intercept (or easiness) parameter $\delta_{1i}$ (Rasch, 1960). The probability that person v will agree with item i is thus

(1) $$p(y_{1vi}=1)=\frac{\exp(\theta_{v}+\delta_{1i})}{1+\exp(\theta_{v}+\delta_{1i})}.$$

Given disagreement or agreement, respectively, the pseudo‐items $Y_{2i}$ and $Y_{3i}$ reflect moderate responses close to the midscale for $Y_{2i}=0$ and $Y_{3i}=0$, as opposed to nonmoderate responses for $Y_{2i}=1$ and $Y_{3i}=1$. Because the choice of a nonmoderate versus moderate response can be driven by an individual response style to prefer or avoid clear‐cut responses in combination with the actual strength of (dis)agreement, the pseudo‐items $Y_{2i}$ and $Y_{3i}$ are parameterized through a multidimensional IRT model (Reckase, 2009; see Meiser et al., 2019, for details). The response style to prefer nonmoderate over moderate response categories is denoted by $\eta^{\text{nm}}$, while the strength of agreement is reflected by the substantive trait θ. As stronger agreement with the item content decreases the probability of a nonmoderate disagreement response but increases the probability of a nonmoderate agreement response, the trait θ enters the model equations for $Y_{2i}$ and $Y_{3i}$ with opposite signs. The probabilities of observing a nonmoderate disagreement or agreement response for person v in item i follow as

(2) $$p(y_{2vi}=1)=\frac{\exp(\eta_{v}^{\text{nm}}-\alpha^{\text{nm}}\cdot \theta_{v}+\delta_{2i})}{1+\exp(\eta_{v}^{\text{nm}}-\alpha^{\text{nm}}\cdot \theta_{v}+\delta_{2i})},$$

and

(3) $$p(y_{3vi}=1)=\frac{\exp(\eta_{v}^{\text{nm}}+\alpha^{\text{nm}}\cdot \theta_{v}+\delta_{3i})}{1+\exp(\eta_{v}^{\text{nm}}+\alpha^{\text{nm}}\cdot \theta_{v}+\delta_{3i})},$$

where the loading parameter $\alpha^{\text{nm}}>0$ allows for a different weight of the trait θ in the nuanced selection of responses within the subset of disagreement or agreement categories compared to the overall (dis)agreement decision on the first node (Meiser et al., 2019).

Contingent on a nonmoderate disagreement or agreement response, the subsequent pseudo‐items $Y_{4i}$ and $Y_{5i}$ model the selection of a nonextreme category with $Y_{4i}=0$ and $Y_{5i}=0$, or an extreme category with $Y_{4i}=1$ and $Y_{5i}=1$. Like the previous decision of choosing a (non‐)moderate category, the decision to select a (non‐)extreme category can be driven by a combination of an individual response style and the substantive trait. The response style to prefer extreme response categories is denoted by $\eta^{\text{e}}$, and the probabilities of observing an extreme disagreement or agreement response for person v in item i are given by

(4) $$p(y_{4vi}=1)=\frac{\exp(\eta_{v}^{\text{e}}-\alpha^{\text{e}}\cdot \theta_{v}+\delta_{4i})}{1+\exp(\eta_{v}^{\text{e}}-\alpha^{\text{e}}\cdot \theta_{v}+\delta_{4i})},$$

and

(5) $$p(y_{5vi}=1)=\frac{\exp(\eta_{v}^{\text{e}}+\alpha^{\text{e}}\cdot \theta_{v}+\delta_{5i})}{1+\exp(\eta_{v}^{\text{e}}+\alpha^{\text{e}}\cdot \theta_{v}+\delta_{5i})}.$$

Following the same rationale as in the decision for (non‐)moderate responses, the trait θ affects extreme disagree and agree responses with opposite signs, and the loading parameter $\alpha^{\text{e}}>0$ reflects the weight of θ in the selection of (non‐)extreme responses relative to the overall agreement node.

In the model Equations (1), (2), (3), (4), (5), we used constant loadings of the latent dimensions across rating items i, thereby following the Rasch model because of its parsimony and unique measurement properties of specific objectivity (see also Meiser et al., 2019). The parameterization of the pseudo‐items can be naturally extended, however, to include item‐specific loading parameters when the strict assumptions of the Rasch model are violated. For the substantive trait θ, variability of loading parameters across rating items can be captured by introducing item‐specific discrimination parameters $\alpha_{i}$ in (1) and item‐specific loading parameters $\alpha_{i}^{\text{nm}}$ and $\alpha_{i}^{\text{e}}$ in Equations (2), (3), (4), (5), respectively. Because the effects of response styles can be defined as being independent of item content, however, we decided to maintain constant loadings of the response style dimensions $\eta^{\text{nm}}$ and $\eta^{\text{e}}$ for theoretical reasons (see Böckenholt & Meiser, 2017, for a similar rationale).

### 1.2 Modeling heterogeneity in response processes

The probability of a given response category for item i results as the product over the pseudo‐items that are involved in the response process along the branches of the IRTree (Böckenholt & Meiser, 2017; Jeon & De Boeck, 2016). Because the model Equations (1), (2), (3), (4), (5) contain different person parameters across the pseudo‐items and even within individual pseudo‐items, the IRTree model accommodates quantitative differences between respondents on several dimensions, that is, the trait to be measured and response styles. However, IRTree models preserve the assumption that the relative weighting of the latent dimensions and the structural item parameters are homogeneous, that is, invariant across the population of respondents. Accounting for differences in the response processes and model parameters between subgroups of respondents can be essential, however, for investigating the nature of response behavior and adjusting psychometric measurement to heterogeneous response strategies.

So far, heterogeneity in the parameters of IRTree models has been analyzed with discrete mixture distribution models for latent subpopulations showing different response processes, like subpopulations with a purely trait‐based response process as opposed to subpopulations with a combination of trait‐based processes and response styles (Alagöz & Meiser, 2023; Khorramdel et al., 2019; Kim & Bolt, 2021; Tijmstra et al., 2018). In the present research, we propose a novel approach to testing for heterogeneity in IRTree models via model‐based partitioning along person covariates. The partitioning method makes it possible to detect different relative weights $\alpha^{\text{nm}}$ and $\alpha^{\text{e}}$ of the substantive trait θ in the selection of (dis)agreement categories between subgroups of respondents. This makes the new method more flexible than the previous mixture distribution approaches, in that all subgroups of respondents can use combinations of latent trait and response style dimensions θ and η, though with different strengths. In addition, partitioning along observed covariates facilitates the analysis of sources of heterogeneity and enhances the interpretability of identified subgroups.

Although in the present research the main focus is on heterogeneity in the loading parameters $\alpha^{\text{nm}}$ and $\alpha^{\text{e}}$, the partitioning method also allows for detecting the variability of other model parameters like the intercept parameters $\delta_{1i}$ to $\delta_{5i}$ in Equations (1), (2), (3), (4), (5). While heterogeneity in $\alpha^{\text{nm}}$ and $\alpha^{\text{e}}$ reflects differential strengths in the effects of individual trait values on the choices of disagree and agree responses, heterogeneity in the intercepts of pseudo‐items indicates overall trends to select specific kinds of response categories that differ among subgroups. In either case, the analysis of heterogeneity in the structural loading and intercept parameters can reveal distinct characteristics of the underlying response behavior in terms of the differentiatedness and overall tendencies of judgments in subgroups of respondents.

In the following sections, we first outline the score‐based partitioning method for testing the invariance of IRT parameters with person covariates. Then we provide evidence for the method's validity for multidimensional IRTree models in simulation studies and illustrate the analysis of heterogeneity with an empirical application of IRTrees for response styles.

## 2 SCORE‐BASED TESTS FOR PARAMETER HETEROGENEITY

Score‐based tests are a widely used family of statistical tests that aim at checking the invariance of maximum likelihood estimates for statistical models in psychometrics that were originally developed in the fields of statistics and econometrics (Andrews, 1993; Hjort & Koning, 2002; Zeileis & Hornik, 2007). Technically, these tests are closely related to the score test (for a technical and historical overview: Rao, 2005). In the field of IRT, in particular Glas suggested numerous applications of the score test (Glas, 1998, 1999, 2001, 2010; Glas & Suárez‐Falcón, 2003; Glas & van der Linden, 2010), although earlier applications of this test in psychometrics can be found (Satorra, 1989). Later work applied score‐based tests for checking parameter invariance in a variety of models, including Bradley–Terry models (Strobl et al., 2011), Rasch and IRT models (Debelak & Strobl, 2019; Komboz et al., 2018; Strobl et al., 2015), models of factor analysis (Merkle et al., 2014; Merkle & Zeileis, 2013; Sterner & Goretzko, 2023; Wang et al., 2014), structural equation models (Arnold et al., 2021; Brandmaier et al., 2013, 2016), and multilevel models (Wang & Merkle, 2023). Variations of score‐based tests were also proposed for Bayesian estimation methods in item response theory (IRT) (Debelak et al., 2022). In another branch of research, these tests were embedded in algorithms for model‐based recursive partitioning (Zeileis et al., 2008), which uses an approach similar to decision trees to detect subpopulations for which model parameters are invariant. For IRT models, such approaches were exemplified by Strobl et al. (2015) and others.

### 2.1 Score‐based tests in IRT(ree) models

The underlying reasoning of score‐based tests in the context of IRT can be described as follows. We consider the responses observed in a sample, together with a metric, ordinal or categorical person covariate, and we are interested in testing the null hypothesis that the item parameter estimates are stable with regard to this person covariate. In a first step, we order the responses with regard to the person covariate. In a second step, we want to investigate fluctuations of the maximum likelihood estimates along the sequence of test takers. This step is based on the premise that for item parameters that are invariant with regard to the person covariate, maximum likelihood estimates do not depend on the value of the person covariate. For any sample taken from the population of respondents, estimates for the item parameters would thus fluctuate randomly around their true values. If, on the other hand, the item parameters are not invariant with regard to a person covariate, there is no single true value of the item parameters, but their true values depend on the value of the person covariate. In this scenario, estimates for the item parameters would fluctuate not around a single true value but around a value that depends on the person covariate. If we would calculate common maximum likelihood estimates for the item parameters, our maximum likelihood estimates would therefore vary systematically from this common estimate.

In the framework of score‐based tests, the score, that is, the gradient of the log‐likelihood, is used to obtain a measure for the fluctuation of maximum likelihood estimates with regard to a person covariate. By definition, maximum likelihood estimates maximize the log‐likelihood; therefore, the gradient of the log‐likelihood at the point of the maximum likelihood estimate has to be 0. For the case of IRTree models, we summarize all item parameters of the pseudo‐items as a vector Ψ, for which we get estimates $\hat{\Psi }$. We further use $y_{1},\text{…},y_{N}$ to denote the response vectors of the N respondents, which summarize the personwise responses to the individual items.

In the context of IRT and other psychometric models, it can further be shown that the score s of the log‐likelihood can be represented as a sum of N individual score contributions, with N being the overall sample size:

(6) $$s(\hat{\Psi };y_{1},\text{…},y_{N})=\overset{N}{\underset{v=1}{\sum }}s(\hat{\Psi };y_{v})=0.$$

Following the outlined reasoning for score‐based tests, we now order these individual score contributions with regard to a person covariate of interest and calculate the cumulative sums of the individual score contributions. In what follows, we write the index in round brackets (v) to indicate this ordering of the individual score contributions. To obtain the cumulative sums, we first use ⌊⌋, denoting the floor function, and a real value t from the interval [0,1] to obtain the value ⌊Nt⌋. This value represents the count of persons in the lowest t fraction of the sample, ordered by the person covariate. The cumulative sums now correspond to terms of the following form:

(7) $$\overset{\left\lfloor Nt\right\rfloor }{\underset{v=1}{\sum }}s(\hat{\Psi };y_{\left(v\right)}).$$

We further decorrelate the individual score contributions, so that their covariance matrix over the whole sample is equal to the unit matrix. If Î is a consistent estimate of the covariance matrix of the individual score contributions, we obtain the following term:

(8) $$B(t,\hat{\Psi })={Î}^{-1/2}N^{-1/2}\overset{\left\lfloor Nt\right\rfloor }{\underset{v=1}{\sum }}s(\hat{\Psi };y_{\left(v\right)}).$$

In this study, we derived Î by initially computing the individual score contributions of all item parameters for all respondents after the convergence of the item parameter estimation via numerical quadrature, using the estfun.AllModelClass() function of mirt (Chalmers, 2012). Subsequently, we obtained Î by estimating the covariance matrix of this sample of individual score contributions, that is, we estimated the covariance matrix using the observed cross‐product of the gradients (Falk & Monroe, 2018).

### 2.2 Testing for heterogeneity across different kinds of covariates

It can be shown that the distribution of $B(t,\hat{\Psi })$ can be described by a standard stochastic process, namely, a Brownian bridge (Hjort & Koning, 2002; Zeileis et al., 2008). This property makes it possible to compare the observed distribution of the individual score contributions with the distribution expected under the null hypothesis. At this point, we need to define a test statistic that summarizes any deviations of the observed distribution of the cumulative score contributions from their expected distribution. Depending on the type of person covariate, a canon of test statistics was proposed for this purpose (Merkle et al., 2016; Wang et al., 2014). Important examples include the double‐maximum statistic, the Cramer–von Mises statistic, and the maximum Lagrange multiplier statistic for settings with a metric person covariate. If we denote the observed score contributions by a matrix $B{\left(\hat{\Psi }\right)}_{vj}$, with v corresponding to the individual respondents and j to the individual item parameters, these statistics are given by

(9) $$DM=\underset{v}{\max}\underset{j}{\max}|B{\left(\hat{\Psi }\right)}_{vj}|,$$

(10) $$CvM=\frac{1}{N}\underset{v}{\max}\underset{j}{\max}{\left(B{\left(\hat{\Psi }\right)}_{vj}\right)}^{2},$$

(11) $$maxLM=\underset{v}{\max}{\left\{\frac{v}{N}\left(1-\frac{v}{N}\right)\right\}}^{-1}.{\left(B{\left(\hat{\Psi }\right)}_{vj}\right)}^{2},$$

CvM and maxLM are only available for models with a limited number of item parameters. Since IRTree models as multidimensional IRT models usually use a large number of item parameters, these statistics would only be applicable for tests with very few items. For this reason, we did not include these statistics in the study at hand. For settings with an ordinal categorical covariate with m categories, there are two test statistics available, namely, the ordered weighted double‐maximum test statistic and the maxLM test for ordinal covariates. To define these test statistics, we use $t_{l},l=1,\text{…},m-1$ to denote the proportion of respondents in the first l categories, and $v_{l}=\left\lfloor N\cdot t_{l}\right\rfloor$. Using this notation, we define these statistics as

(12) $$\begin{array}{ll} WDM_{o} & =\underset{v\in \{v_{1},\text{…},v_{m-1}\}}{\max}{\left\{\frac{v}{N}\left(1-\frac{v}{N}\right)\right\}}^{-1/2}\underset{j=1,\text{…},k}{\max}|B{\left(\hat{\Psi }\right)}_{vj}|, \end{array}$$

(13) $$\begin{array}{ll} maxLM_{o} & =\underset{v\in \{v_{1},\text{…},v_{m-1}\}}{\max}{\left\{\frac{v}{N}\left(1-\frac{v}{N}\right)\right\}}^{-1}\underset{j=1,\text{…},k}{\sum }{\left(B{\left(\hat{\Psi }\right)}_{vj}\right)}^{2}. \end{array}$$

For settings with an unordered categorical covariate with m categories, an unordered Lagrange Multiplier test is available as a test statistic. This statistic is given by Merkle et al. (2014) and Wang et al. (2014)

(14) $$LM_{uo}=\underset{l}{\sum }\underset{j}{\sum }{\left(B{\left(\hat{\Psi }\right)}_{v_{l}j}-B{\left(\hat{\Psi }\right)}_{v_{l-1}j}\right)}^{2}.$$

For any of these test statistics, p‐values for testing the null hypothesis of parameter invariance can be obtained by analytical considerations (DM, $WDM_{o}$, and $LM_{uo}$) or simulations (maxLM, CvM, $maxLM_{o}$) via the strucchange package in R (Zeileis et al., 2002). Technical details on the calculation of the p‐values for the various test statistics are provided, for instance, by Wang et al. (2014). By calculating these test statistics only for a subset of the item parameters, it is further possible to check the invariance of individual item parameters or selections of parameters (Schneider et al., 2022). This property can be used for specific tests of invariance of item parameters corresponding to factor loadings and intercepts of pseudo‐items in an IRTree model by calculating these test statistics selectively for loading and intercept parameters, respectively.

In the remainder of this article, we will evaluate the approach with simulation studies and an empirical application. In particular, the DM statistic is used when testing for parameter heterogeneity in a metric covariate, the $LM_{uo}$ statistic when testing for parameter heterogeneity in an unordered categorical person covariate, and the $WDM_{o}$ and $maxLM_{o}$ statistics when testing for parameter heterogeneity in an ordinal person covariate.

After detecting heterogeneity in the model parameters, we further used the following partitioning algorithm, which is based on an algorithm of Strobl et al. (2015), to detect at which value of the person covariate the parameters change. First, we defined all possible split points for the person covariate that allowed us to split the sample into two subsamples of 300 or more respondents. Second, we estimated for each of these split points the item parameters of the IRTree models in both subsamples separately. Here, all item parameters are allowed to differ between both estimated models. This step leads to two IRTree models for each split point. In a final step, we calculate the sum of the log‐likelihoods of the two IRTree models for each cutpoint and select the cutpoint for which this sum is maximized. This cutpoint is interpreted as the value where the model parameters change.

## 3 GOALS OF CURRENT RESEARCH

The proposed new approach to testing for parameter heterogeneity in IRTree models will allow researchers to identify subgroups of respondents that differ in response behavior and, thus, to tailor psychometric models to optimally measure the traits of interest. In particular, heterogeneity can be observed with respect to the relative weighting of trait‐based response processes in the selection of disagree and agree categories via the parameters $\alpha^{\text{nm}}$ and $\alpha^{\text{e}}$ (Equations (2), (3), (4), (5)). Whereas previous mixture distribution approaches for IRTree models analyzed differences in the use of latent trait and response style dimensions in a binary yes‐or‐no fashion by constraining specific dimension weights to zero (Alagöz & Meiser, 2023; Khorramdel et al., 2019; Kim & Bolt, 2021; Tijmstra et al., 2018), the new score‐based partitioning algorithm can identify subgroups with more subtle differences in the influence of response dimensions. Moreover, while the focus of the present research is on differences in the loading parameters $\alpha^{\text{nm}}$ and $\alpha^{\text{e}}$, the partitioning algorithm can also reveal heterogeneity in the intercept parameters of pseudo‐items δ, reflecting differences between subgroups concerning overall tendencies to select or avoid certain kinds of response categories.

The proposed application of score‐based tests differs significantly from previous applications of score‐based tests in IRT (e.g., Debelak & Strobl, 2019) in at least three respects. The underlying IRT models are (a) significantly more complex and (b) estimated with the Metropolis–Hastings Robbins–Monro estimation algorithm. Finally, our tests (c) aim at detecting changes in specific item parameters instead of being omnibus tests for parameter heterogeneity. To demonstrate the validity and suitability of the new approach, in what follows, we evaluate score‐based tests for IRTree models of response styles in simulation studies and illustrate their application with an empirical example. The R Code for the new method, simulations, and empirical example is available in an OSF repository.

## 4 SIMULATION DESIGN

To evaluate the new method, we carried out three simulation studies that investigated the power and Type I error of score‐based tests for parameter heterogeneity in IRTree models for response styles between two groups. The first simulation study investigated parameter heterogeneity with regard to an unordered categorical covariate.

We investigated all combinations of the following conditions:

- *Test length*: The test consisted of 30 or 50 pseudo‐items. As each rating item is represented by five pseudo‐items as defined in Equations (1), (2), (3), (4), (5), this corresponds to scenarios with 6 or 10 rating items, respectively.
- *Sample size*: The test was worked on by 1000 or 2000 respondents.
- *Factor loading of trait in nonmoderate responses*: The factor loading $\alpha^{\text{nm}}$ for the tendency to give nonmoderate responses (Equations 2 and 3) was either .2 or .8.
- *Factor loading of trait in extreme responses*: The factor loading $\alpha^{\text{e}}$ for the tendency to give extreme responses (Equations 4 and 5) was either .2 or .8.
- *Parameter heterogeneity*: In our simulations, either all model parameters were invariant or the parameters $\alpha^{\text{nm}}$ and $\alpha^{\text{e}}$ increased or decreased by .2 or by .5 for all respondents of the second group, which represents the focal group.

Each respondent was randomly assigned to one of the two groups, with each member having a probability of .5 of being assigned to each group. There were two latent personality traits of interest, of which one had nonzero factor loadings on the first and the other on the second half of the rating items. Together with the two latent factors that modeled response styles, we therefore obtained four latent dimensions, which were assumed to be drawn from a multivariate normal distribution with mean vector **0**. The variance covariance matrix was chosen to be

$$\text{Cov}=\left(\begin{array}{cccc} 2.89 & 1.02 & .00 & .00\\ 1.02 & 1.44 & .00 & .00\\ .00 & .00 & 1.00 & .75\\ .00 & .00 & .75 & 2.25 \end{array}\right),$$

with latent correlations of ρ=.5 between the two traits (Dimensions 1 and 2) and the two response styles (Dimensions 3 and 4). The simulated scenario thereby resembled earlier empirical analyses with two correlated traits of different variance and with latent dimensions of nonmoderate and extreme response styles, where the variance of extreme responding exceeds the variance of nonmoderate responding (Böckenholt & Meiser, 2017; Meiser et al., 2019).

The intercept parameters were drawn from a uniform distribution U(−1,1) for $\delta_{i1}$ in the agreement node, from a uniform distribution U(−1,.5) for $\delta_{i2}$ and $\delta_{i3}$ in the nonmoderate response nodes, and from a uniform distribution U(−2.5,−1.5) for $\delta_{i4}$ and $\delta_{i5}$ in the extremity nodes (Equations (1), (2), (3), (4), (5)). The slope and intercept parameters remained constant for all conditions with tests of the same length.

The second simulation study investigated the detection of parameter heterogeneity with regard to a metric covariate, which was drawn from a uniform distribution U(20,80). If parameter heterogeneity was present, it affected the persons up to a cutoff value of 40. The other settings were identical to those of the first simulation study.

In the third simulation study, we focused on detecting parameter heterogeneity with regard to an ordinal covariate, which was represented by integer values ranging from 1 to 5. We simulated two distinct scenarios based on the probability distribution of observing each category of the covariate. In the first scenario, each category had an equal probability of .2, leading to a uniform distribution across the five possible values. In the second scenario, we implemented a nonuniform distribution, characterized by the relative frequencies of the categories as follows: .1 for Categories 1 and 2, .35 for Categories 3 and 4, and .1 for Category 5. If parameter heterogeneity was present, the item parameters differed between persons with a covariate category of 3 or less and persons with a category of 4 or higher. The other settings were identical to those of the first two simulation studies.

For each setting of the three simulation studies, we applied two separate score‐based tests to test for parameter heterogeneity in $\alpha^{\mathrm{nm}}$ and $\alpha^{e}$, leading to two p‐values in each simulated data set. These tests considered only the individual score contributions for the respective item parameters and thus implemented the approach outlined in the introduction, where test statistics of score‐based tests are only calculated for a subset of the item parameters. We evaluated the rate of p‐values below .05 for each setting, which led to an evaluation of the Type I error and the power of both statistical tests under each setting.

In the second and third simulation studies, we further investigated the accuracy of the algorithm used for the split point detection. In this evaluation, we first determined the split point based on the algorithm outlined in the previous section if true parameter heterogeneity was detected by the corresponding statistical test. The result of the second statistical test was ignored for this evaluation. For each condition with simulated parameter heterogeneity effects and positive statistical tests, we calculated the mean and the standard deviation of the split points and compared it to the true value of 40 in the second simulation study and 3 in the third simulation study.

In all simulation studies, the model parameters were estimated using the Metropolis–Hastings Robbins–Monro algorithm (Cai, 2010) using the default settings for this algorithm in mirt.^1^ This algorithm is computationally more efficient for parameter estimation in high‐dimensional IRT models, such as IRTree models, than classical methods such as marginal maximum likelihood estimation based on an expectation‐maximization algorithm (Bock & Aitkin, 1981). For scaling the item and person parameters, this algorithm uses a normal prior with mean 0 for the person parameters of the multidimensional IRT model. The variance covariance matrix of the latent variables was freely estimated in all simulations.

## 5 RESULTS

We first discuss results on the power and the Type I error rate, and then we discuss the accuracy of the split point detection. Additional plots are presented in the files provided as supporting information for this article.

### 5.1 Type I error and power

**FIGURE 2:**
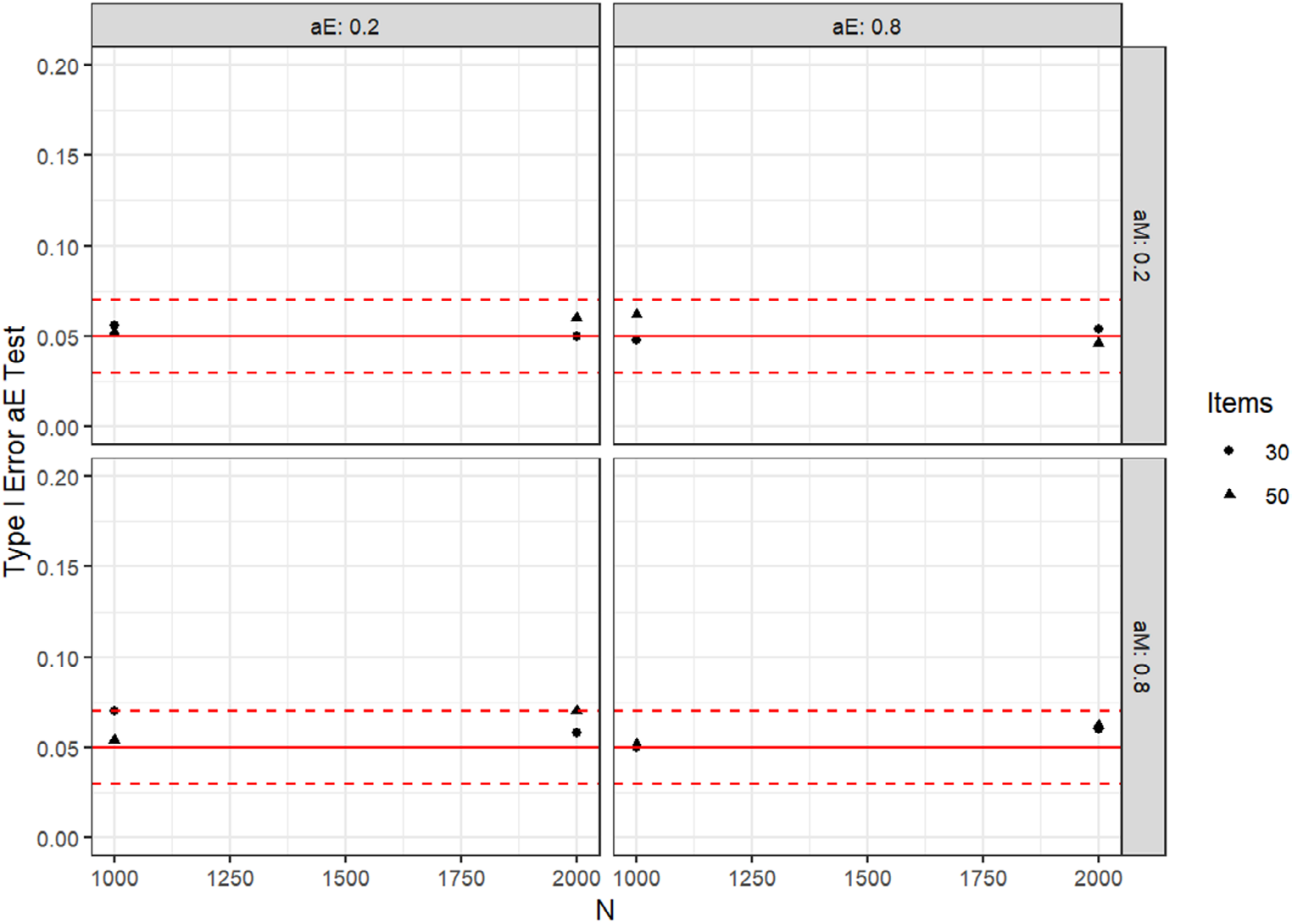
Type I error when testing for parameter changes in $\alpha^{e}$ when all parameters are invariant. Red lines indicate nominal alpha level of .05 and approximate 95% confidence interval for the hit rates.

If parameter heterogeneity was absent in the factor loading tested for invariance, the Type I error was slightly increased, but usually close to the nominal alpha level of .05. This overall result was found independently of which factor loading was tested for changes and also independently of whether heterogeneity was present in the other factor loading. As an illustration, we present the results for conditions with a metric covariate in the following Figure 2 when testing for parameter changes in $\alpha^{e}$ under conditions with no parameter changes in both factor loadings. For conditions investigating categorical covariates with the DM statistic, the Type I error was usually close to the nominal alpha level. Under conditions with an ordinal person covariate, the score‐based tests based on the test statistics WDMo and maxLMo usually had a slightly increased Type I error, in particular when the distribution of the covariate categories was skewed.

**FIGURE 3:**
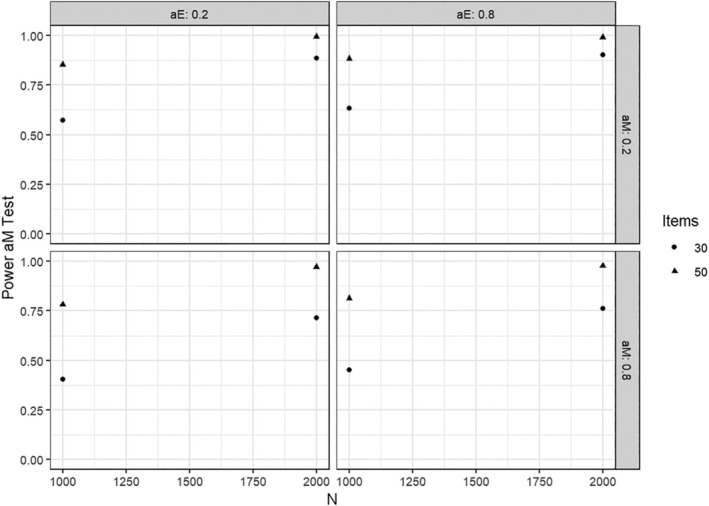
Power when testing for parameter changes in $\alpha^{\mathrm{nm}}$ when this parameter changed by +.2.

**FIGURE 4:**
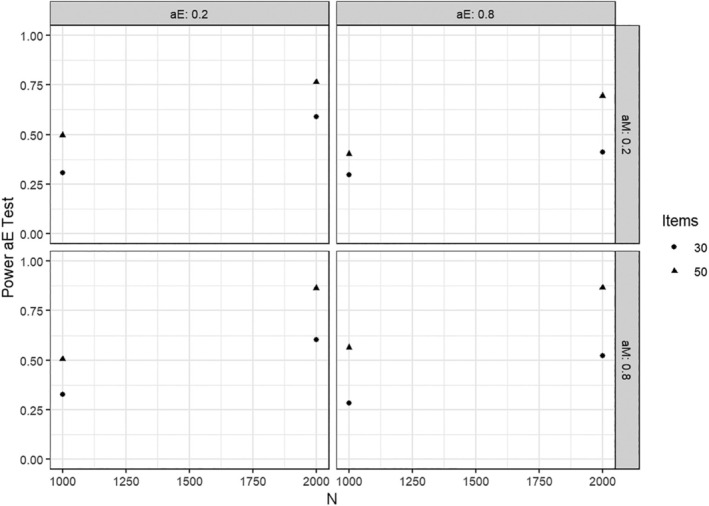
Power when testing for parameter changes in $\alpha^{e}$ when this parameter changed by +.2.

When a parameter change of .5 was present in a factor loading tested for heterogeneity, the power of the corresponding test was usually close to 1. We therefore focus on the results of tests in conditions with a parameter change of .2, which are summarized in the following Figures 3 and 4. As can be seen, the tests had considerable power to detect this type of effect. The power to detect parameter changes in $\alpha^{\mathrm{nm}}$ was slightly larger than that to detect parameter changes in $\alpha^{e}$. This difference was due to the hierarchical nature of the IRTree model, where all rating responses provide information for the pseudo‐items of nonmoderate responding, but only a subset of responses provides information for the pseudo‐items of extreme responding (Figure 1). Under conditions with a larger sample size and test length, the power was slightly increased overall. Again, very similar results were found in the simulation studies with categorical and ordinal covariates, and we omit details for brevity.

### 5.2 Cutpoint selection

**FIGURE 5:**
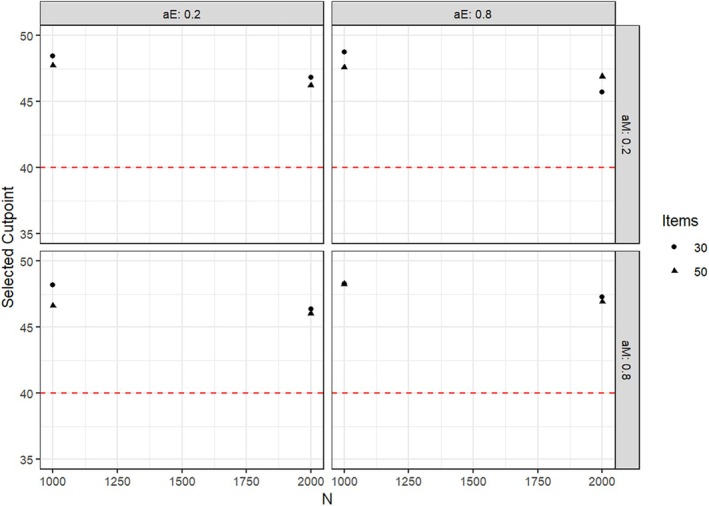
Mean proposed cutpoint under different conditions of sample size, test length, and factor loadings when $\alpha^{e}$ was affected by a parameter change of +.2. The red line denotes the true cutpoint of 40.

**FIGURE 6:**
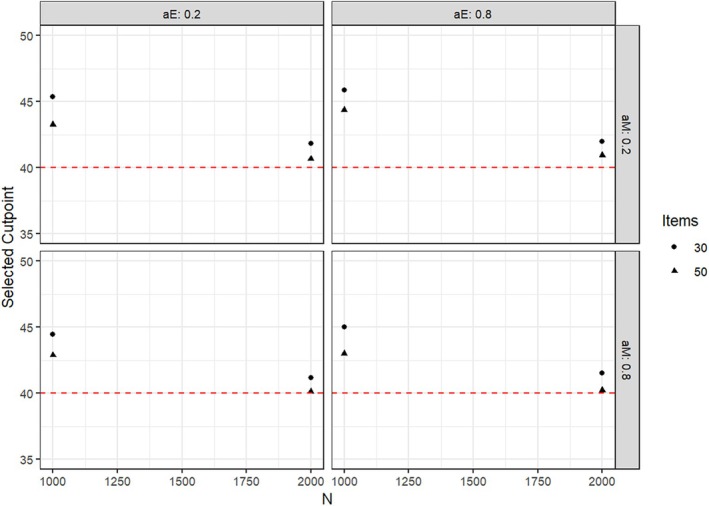
Mean proposed cutpoint under different conditions of sample size, test length, and factor loadings when $\alpha^{e}$ was affected by a parameter change of +.5. The red line denotes the true cutpoint of 40.

Under all conditions, the mean cutpoint proposed for splitting the groups affected by parameter heterogeneity was larger than the true value of 40, but lower than the value of 50, which would split the sample into two groups of the same size. This bias decreased with the sample size and with the size of the actual parameter change. As an illustration, Figures 5 and 6 present detailed results for the cutpoint selection for parameter heterogeneity tests for $\alpha^{e}$ when this parameter was affected by a parameter change of +.2 and +.5, respectively. Analogous results were obtained for changes in $\alpha^{\mathrm{nm}}$ and in the simulation study with an ordinal covariate.

## 6 EMPIRICAL EXAMPLE

Eid and Rauber (2000) analyzed data from an organizational survey with a six‐item questionnaire on employees' satisfaction with their superiors. Responses were observed on a six‐point rating scale, and the authors analyzed the data with a mixture distribution partial credit model (Rost, 1991). Model selection via the Bayesian information criterion revealed a two‐class solution, where the class‐specific threshold parameters indicated a response tendency to use nonextreme response categories for the larger class and a tendency toward extreme categories for the smaller class. Moreover, the authors found relationships of class assignment with demographic and job‐related person covariates, including the covariate “length of service on the same position” with five ordinal categories (≤6, 7–12 months, 1–5, 5–10, >10 years).

Here we reanalyze the data from Eid and Rauber (2000) with the IRTree model in Figure 1 and test for parameter heterogeneity using the new score‐based partitioning method.^2^ Unlike the mixed partial credit model in the original analysis, the IRTree model accommodates nonmoderate and extreme response styles as continuous dimensions, so that parameter heterogeneity is disentangled from individual response styles. A sample of N=3964 respondents was available with observed values in the six items and in the covariate “length of service on the same position.” Parameter estimates for the IRTree model in Figure 1 were obtained with the Metropolis–Hastings Robbins–Monro algorithm of the R package mirt using default convergence settings (Chalmers et al., 2023), that is, a convergence threshold of .001. The joint distrbution of the person parameter vector was specified as ${\left(\theta ,\eta^{\text{nm}},\eta^{\text{e}}\right)}^{\prime }∼N(\text{0},\sum )$ with freely estimated variance covariance matrix ∑. Parameter estimation of the IRTree model in Figure 1 showed loadings for the trait θ in the pseudo‐items of nonmoderate responding (Equations 2 and 3) and extreme responding (Equations 4 and 5) of ${\hat{\alpha }}^{\text{nm}}=.761$ and ${\hat{\alpha }}^{\text{e}}=.613$. These loadings refer to the common trait θ in the model equations for the agreement, nonmoderate, and extreme nodes of the IRTree and can be interpreted in relation to the loading of 1.0 in the agreement node (Equation 1). The results therefore replicate earlier findings that the trait has a smaller weight for gradual choices among (dis)agreement categories relative to the pseudo‐item of general agreement versus disagreement (Meiser et al., 2019).

Score‐based tests for parameter heterogeneity in $\alpha^{\text{nm}}$ and $\alpha^{\text{e}}$ with the person covariate “length of service on the same position” and the test statistic $LM_{uo}$ in Equation (14) indicated significant heterogeneity for $\alpha^{\text{nm}}$, f(efp)=9.80, p=.007, but not for $\alpha^{\text{e}}$, f(efp)=.29, p=.961. Analogously to the tests evaluated in the simulation studies, the score‐based tests used for the empirical data examined parameter heterogeneity in individual loading parameters. As a next step, we applied the partitioning algorithm to find respondent groups for which the item parameter vector of the IRTree model is invariant. The partitioning algorithm located the cutoff point at length of service up to 5 years as opposed to longer than 5 years. Subsequent score‐based tests within the two subgroups did not reveal further significant heterogeneity in $\alpha^{\text{nm}}$ for respondents with length of service up to 5 years, f(efp)=1.01, p=.482, or for respondents with length of service longer than 5 years, f(efp)=3.23, p=.071.

The resulting subgroups showed estimated trait loadings of ${\hat{\alpha }}^{\text{nm}}=.777$ and ${\hat{\alpha }}^{\text{nm}}=.754$ for respondents up to 5 years and more than 5 years of service on the same position, respectively. The estimates suggest that respondents with a shorter period on the same position engage in a stronger trait‐based response process in their choices of nonmoderate versus moderate (dis)agreement categories relative to their general (dis)agreement decision compared to respondents with a longer period on the same position. Although the score‐based test was only significant for the loading parameter $\alpha^{\text{nm}}$, descriptively we found the same pattern for $\alpha^{\text{e}}$ in the choices between extreme versus nonextreme (dis)agreement categories, with ${\hat{\alpha }}^{\text{e}}=.653$ and ${\hat{\alpha }}^{\text{e}}=.587$ for respondents up to 5 years or more than 5 years on the same position.

In addition to heterogeneity in the factor loadings, the partition of the sample also captures heterogeneity in the intercept parameters of the pseudo‐items. In contrast to differences in the loading parameters, which reflect the impact of individual differences in θ on the observed responses, differences in the intercept parameters indicate general tendencies to select or avoid certain kinds of categories that characterize subgroups of respondents. For the agreement node, the mean estimated intercept parameter $\delta_{1i}$ across the six rating items was 2.100 for the first subgroup and 1.800 for the second subgroup, indicating that respondents with up to 5 years of service had a stronger tendency to endorse the positive statements about their superiors compared to respondents with longer periods of service. The mean intercepts of the nonmoderate disagreement and agreement nodes $\delta_{2i}$ and $\delta_{3i}$ were −2.473 and .335 for the first subgroup and −2.376 and .160 for the second group, suggesting that respondents with shorter service on the same position had stronger overall tendencies to avoid clear‐cut disagreement and to show clear‐cut agreement than respondents who had served longer on the same position. Finally, the mean intercepts for extreme disagreement and agreement decisions $\delta_{4i}$ and $\delta_{5i}$ were −3.782 and −1.788 for the first subgroup and −2.959 and −2.283 for the second subgroup, which signals a stricter tendency to avoid extreme disagreement and a less strict tendency to avoid extreme agreement categories for respondents with shorter service on the same position. Together the differences in the various intercept parameters reveal that respondents with up to 5 years of service were more inclined to avoid negative ratings and to give favorable ratings than respondents with more than 5 years of service.

It has recently been demonstrated that differences in structural item parameters along the branches of sequential IRTree decision nodes may reflect changes in the underlying person distribution (Lyo et al., 2023), which, among other factors, could be due to sample selection effects (Meiser & Reiber, 2023). Likewise, in the present context of model‐based partitioning, differences in the item parameters between subgroups can mirror variations in the person distribution, as is obvious for the reported differences in the intercept parameters: Inasmuch as the differences consistently indicated a tendency to less negative and more positive ratings for the first subgroup relative to the second subgroup, the differences in the intercept parameters may be driven by higher average values for the trait θ in the first group. That is, because the expectation of θ is fixed to zero in either group for technical reasons of identifiability, group differences in θ may manifest themselves in shifts of threshold parameters. To demonstrate this point, and to safeguard our interpretation of the differences in loading parameters against alternative interpretations as mere reflections of alterations in the person distribution, we conducted an auxiliary multigroup analysis. In this analysis, we fixed the expectation vector of the person parameters to zero only for one subgroup and constrained the intercept parameters to be equal across subgroups while leaving the variance covariance matrix free between groups. This specification allowed us to guarantee parameter identification while at the same time accounting for potential differences in the person distribution. In particular, the expectation of θ was estimated as a free parameter for the subgroup of respondents with up to 5 years of service on the same position. The estimated expectation of θ for this subgroup increased to .324 in the multigroup analysis, thereby showing the greater satisfaction of respondents on the trait level. The higher trait level may indicate a change effect with greater satisfaction at a more recently acquired position that deteriorates over time, or it may be due to a selection effect such that employees with higher satisfaction are more likely to be promoted to the next position within a period of 5 years. At the same time, the expectations of nonmoderate and extreme response styles $\eta^{\text{nm}}$ and $\eta^{\text{e}}$ were estimated at −.240 and −.438 in the subgroup of respondents with up to 5 years of service, indicating more moderate and less extreme response behavior presumably due to less judgment confidence given the shorter experience with superiors. Importantly, however, the direction of the differences in the loading parameters $\alpha^{\text{nm}}$ and $\alpha^{\text{e}}$ on the trait θ was replicated in the modified model specification, with ${\hat{\alpha }}^{\text{nm}}=.810$ and ${\hat{\alpha }}^{\text{nm}}=.737$ and ${\hat{\alpha }}^{\text{e}}=.705$ and ${\hat{\alpha }}^{\text{e}}=.565$ in the subgroups with up to 5 years versus more than 5 years of service.

Finally, we used a model with item‐specific loading parameters on the substantive trait θ to demonstrate the score‐based testing and partitioning approach in a generalized framework. For this purpose, the variance of the trait dimension θ was fixed at 1.0, and item‐specific loading parameters on this dimension were specified for the pseudo‐items in Equations (1), (2), (3), (4), (5). Score‐based tests were applied to the subsets of loading parameters referring to the pseudo‐items of nonmoderate responding and extreme responding, respectively. Replicating the previous findings, the tests revealed significant heterogeneity along the person covariate for the loading parameters in the pseudo‐items for nonmoderate responding, f(efp)=17.26, p=.031, but not for extreme responding, f(efp)=13.68, p=.118. The partitioning algorithm identified the cutoff at the same point as before, that is, between up to 5 years as opposed to more than 5 years of service on the same position. Mean estimated loading parameters for the trait dimension θ across the six rating items were 3.001, 2.216, and 1.776 for the agree, nonmoderate, and extreme pseudo‐items in the first subgroup and 3.369, 2.340, and 1.800 in the second subgroup. These results indicate a larger differentiatedness of general agreement responses in the second subgroup with a longer period of service, presumably due to more experience with their superiors and, thus, higher judgment certainty. In line with the previous results, however, the trait loadings showed a steeper decrease from the superordinate agreement node to the nonmoderate and extreme decision nodes in the second subgroup with more than 5 years of service compared to the first subgroup with up to 5 years of service on the same position.

## 7 DISCUSSION

IRTree models can accommodate quantitative individual differences in response styles, such as nonmoderate or extreme response tendencies, and thereby allow researchers to disentangle the measured traits of interest from additional response processes (Böckenholt, 2017; Böckenholt & Meiser, 2017; Khorramdel & von Davier, 2014; Meiser et al., 2019; Plieninger & Meiser, 2014). Like other IRT model families, however, IRTree models are based on the premise that the structural item parameters are homogeneous across the entire population of respondents, so that the loading weights of the latent dimensions are assumed invariant over subgroups. As a consequence, standard IRTree models cannot account for heterogeneity in the composition or weighting of response processes across respondents. Such heterogeneity can be essential, however, for theoretical and practical reasons. First, existing sources of heterogeneity are of interest for analyzing the nature of response processes that underlie observed rating responses in psychological assessment and surveys. Second, accounting for heterogeneity in the parameters affords a tailored measurement model for the target traits that optimally fits the individual response sequence.

In this research, we introduced and evaluated a partitioning approach for IRTree models that captures parameter heterogeneity in pseudo‐items as a function of observed person covariates. The proposed partitioning method builds on score‐based tests that have been used for tests of parameter invariance in unidimensional IRT models with maximum likelihood estimation (Debelak & Strobl, 2019; Komboz et al., 2018; Schneider et al., 2022; Strobl et al., 2015). Here we extended this method to IRTree models with a multidimensional latent space across and within pseudo‐items and to parameter estimation with a Metropolis–Hastings Robbins–Monro algorithm that is recommended for complex models of high dimensionality (Cai, 2010) and that can be used in the R package mirt (Chalmers, 2012; Chalmers et al., 2023). In contrast to earlier studies on score‐based tests and model‐based recursive partitioning (e.g., Debelak & Strobl, 2019), our study also focused on tests that were sensitive to heterogeneity in individual item parameters. This possibility was previously mentioned by Schneider et al. (2022) but has not yet been systematically evaluated.

The simulation studies generally demonstrated accurate Type I error rates and sufficient power of the score‐based tests for invariance of the factor loadings, which formed the focus of the present research. We only found a handful of conditions where the Type I error was slightly increased, for instance, when parameter heterogeneity with respect to an ordinal person covariate was investigated. This finding might be related to the use of the Metropolis–Hastings Robbins–Monro algorithm that might cause numerical inaccuracies in the resulting score‐based tests. An anonymous reviewer pointed us to the possibility that additional fine‐tuning of this algorithm (Ju & Falk, 2019; Monroe & Cai, 2014) and other options for estimating the covariance matrix of the individual score contributions (Falk & Monroe, 2018; Guastadisegni et al., 2022; Paek & Cai, 2014) might also help to obtain accurate Type I error rates under these conditions. Importantly, our simulation studies demonstrated that the score‐based tests were able to distinguish between parameter heterogeneity in $\alpha^{\text{nm}}$ and $\alpha^{\text{e}}$. The empirical example illustrated that the partitioning algorithm could reveal heterogeneity between subgroups in the factor loadings for real data, such that variation was detected in the weighting of the substantive trait for gradual category choices of disagreement and agreement categories. In addition, general tendencies to prefer or avoid specific categories were reflected in the estimated intercept parameters of the subgroups and may indicate differences in the latent person distributions. Unlike the mixture distribution models proposed to capture heterogeneity in IRTree models (e.g., Alagöz & Meiser, 2023; Kim & Bolt, 2021), the model‐based partitioning approach allows that both substantive traits and response styles contribute to the decisions in the pseudo‐items for all subgroups. Moreover, because the partitioning algorithm exploits extraneous person covariates, it facilitates the analysis of sources or concomitants of parameter heterogeneity, and it circumvents common problems of mixture distribution models like local maxima and extensive sample size requirements.

In this paper, we introduced score‐based tests to analyze heterogeneity in the factor loadings of substantive traits by means of an IRTree model that accounts for nonmoderate and extreme response styles in six‐point rating responses (Böckenholt, 2017; Meiser et al., 2019). The proposed score‐based testing approach and corresponding partitioning algorithm can easily be adapted to other IRTree model structures and parameters, however, like IRTree models for four‐, five‐, or seven‐point rating scales and models with estimated loadings for both trait and response style dimensions (Khorramdel & von Davier, 2014; Merhof & Meiser, 2023; Plieninger & Meiser, 2014). Over and above generalizing the score‐based partitioning algorithm to different IRTree model structures and model specifications, future research could extend the systematic investigation of its statistical properties like Type I error rate and power for tests of invariance of other structural parameters in IRTree modeling. While our focus was on heterogeneity in the factor loadings of the substantive trait, future analysis might focus on heterogeneity in the intercept parameters or in the loading parameters for other response processes.

To conclude, this research introduced a new approach to testing for parameter heterogeneity in multidimensional IRTree models. The new method has proven to be a promising tool that can be used to investigate the nature and sources of differences in response processes and to account for such differences in the measurement of psychological traits.

## AUTHOR CONTRIBUTIONS

**Rudolf Debelak:** methodology; visualization; writing – review and editing; writing – original draft; formal analysis; resources; software; investigation; conceptualization; supervision. **Thorsten Meiser:** conceptualization; investigation; funding acquisition; writing – original draft; methodology; visualization; writing – review and editing; software; formal analysis; project administration; resources; data curation. **Alicia Gernand:** investigation; writing – original draft; writing – review and editing; formal analysis.

## CONFLICT OF INTEREST STATEMENT

The authors declare that they have no conflict of interest.

## COMPUTATIONAL DETAILS

All results were obtained using the R system for statistical computing version 4.4.0 (R Core Team, 2024), employing the add‐on packages (in alphabetical order) mirt version 1.41 (Chalmers, 2012), SimDesign version 2.15 (Chalmers & Adkins, 2020), and strucchange version 1.5‐3 (Zeileis et al., 2002) in the simulation study. The figures were created using ggplot2 (Wickham, 2016) version 3.5.1.

## Supporting information


Data S1.

Data S2.

## Data Availability

The simulated data and the code that support the findings of this study are openly available in OSF at https://osf.io/fpt7e/?view_only=e47933453ded45b0b5434ceb37409b75. The data of the empirical example can be obtained from https://www.hogrefe.com/us/ejpa/special‐features.

## Footnotes

^1^
At the time of writing this paper, these default settings use 150 burn‐in cycles in Stage 1 of the algorithm, 100 SEM cycles in Stage 2, and 5 Metropolis–Hastings draws at each iteration. The estimation algorithm uses a maximum of 2000 iterations and a convergence threshold of .001.

^2^
The original data can be retrieved from: https://www.hogrefe.com/us/ejpa/special‐features.
