# Supplementary material for: Investigating heterogeneity in IRTree models for multiple response processes with score‐based partitioning
Source: Br J Math Stat Psychol. 2024 Nov 4;78(2):420–39. doi: 10.1111/bmsp.12367 (PMC11971602; doi:10.1111/bmsp.12367)

Supplementary Material: Investigating Heterogeneity in IRTree Models for Response Styles with Score-Based Partitioning

This document provides the complete results on the Type I error and power for all conditions described in the main text. We sort the results by type of covariate and test statistic as well as the specific condition.

Numerical Covariate, Test Statistic DM

Parameter Invariance

Figure 1

Type I Error when testing for parameter changes in α^e when all parameters are invariant. Red lines indicate the nominal alpha level of 0.05 and an approximate 95% confidence interval for the hit rates.

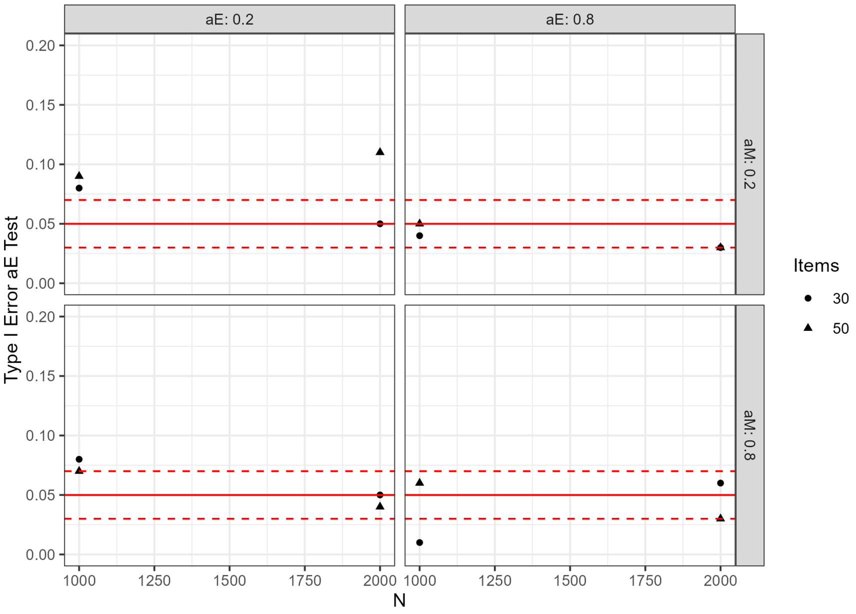

Figure 2

Type I Error when testing for parameter changes in α^{nm} when all parameters are invariant. Red lines indicate the nominal alpha level of 0.05 and an approximate 95% confidence interval for the hit rates.

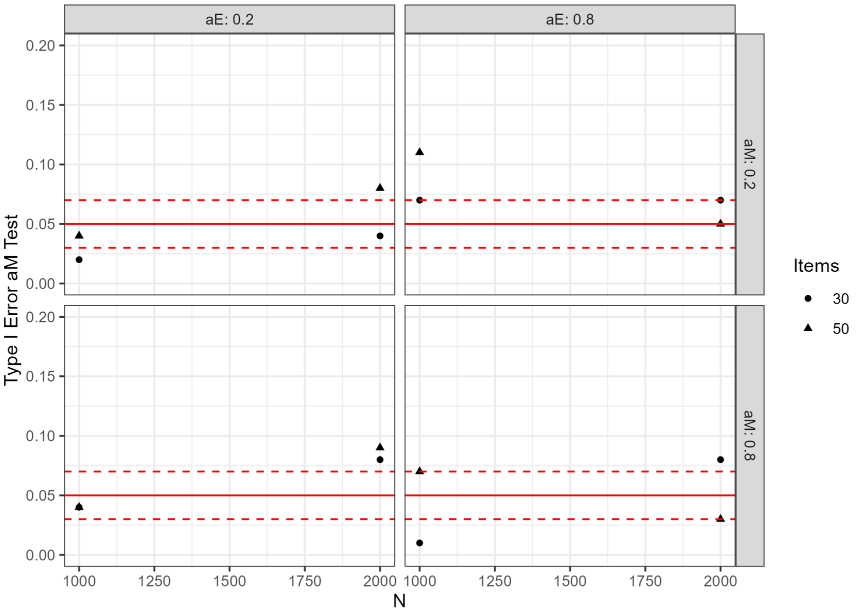

Change of Non-Moderate Response Parameter by +0.5

Figure 3

Type I Error when testing for parameter changes in α^e when α^{nm} changed by +0.5.

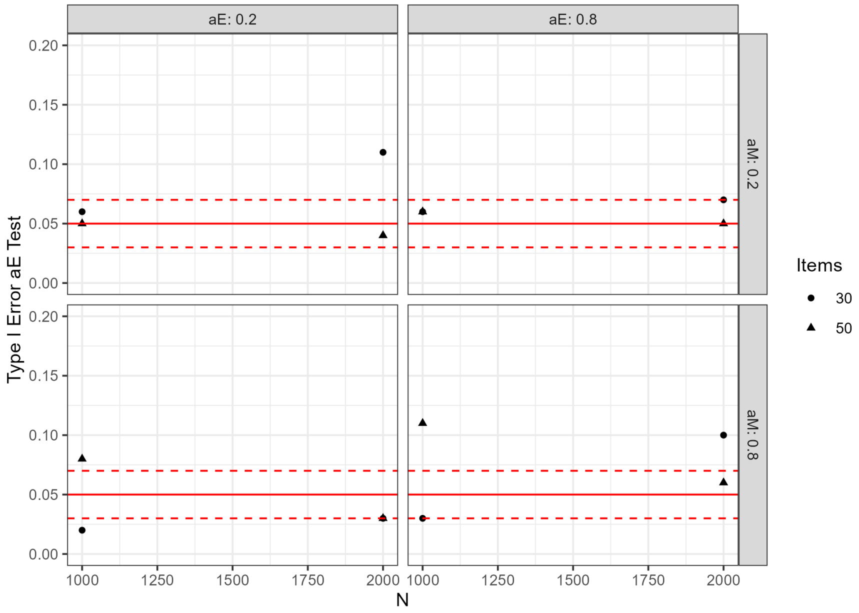

Figure 4

Power when testing for parameter changes in α^{nm} when α^{nm} changed by +0.5.

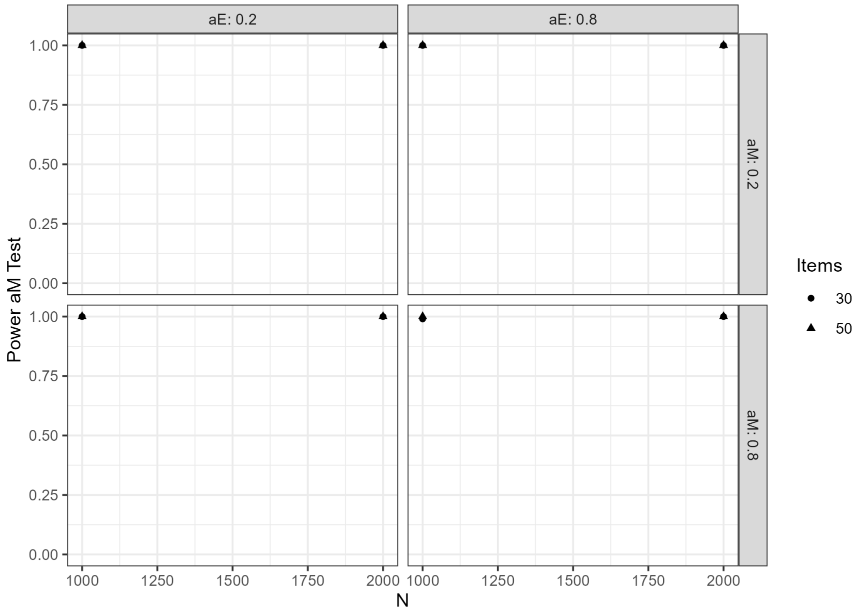

Change of Non-Moderate Response Parameter by +0.2

Figure 5

Type I Error when testing for parameter changes in α^e when α^{nm} changed by +0.2.

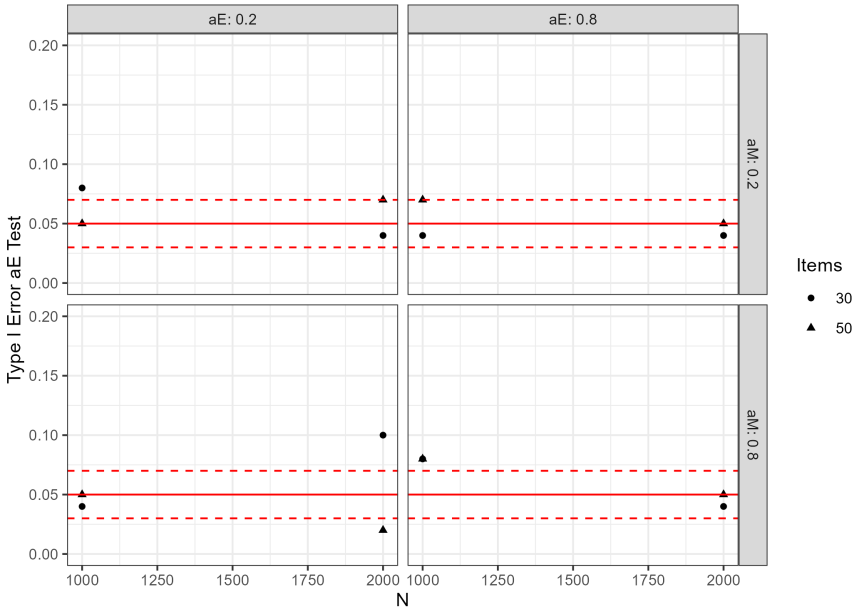

Figure 6

Power when testing for parameter changes in α^{nm} when α^{nm} changed by +0.2.

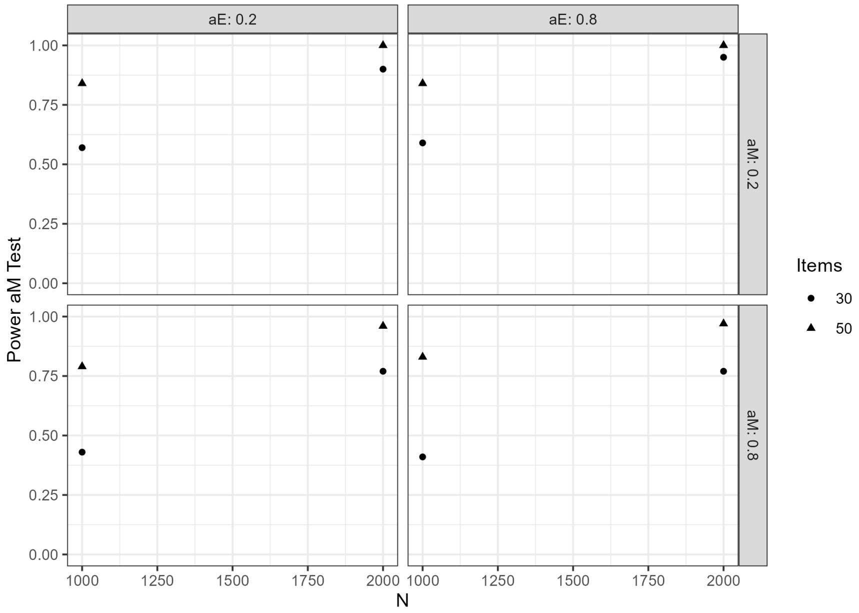

Change of Non-Moderate Response Parameter by -0.5

Figure 7

Type I Error when testing for parameter changes in α^e when α^{nm} changed by -0.5.

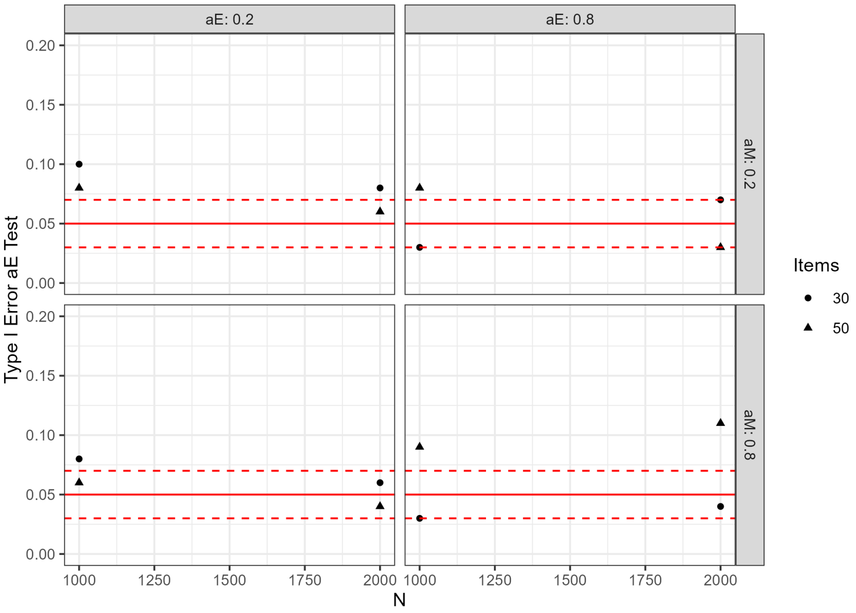

Figure 8

Power when testing for parameter changes in α^{nm} when α^{nm} changed by -0.5.

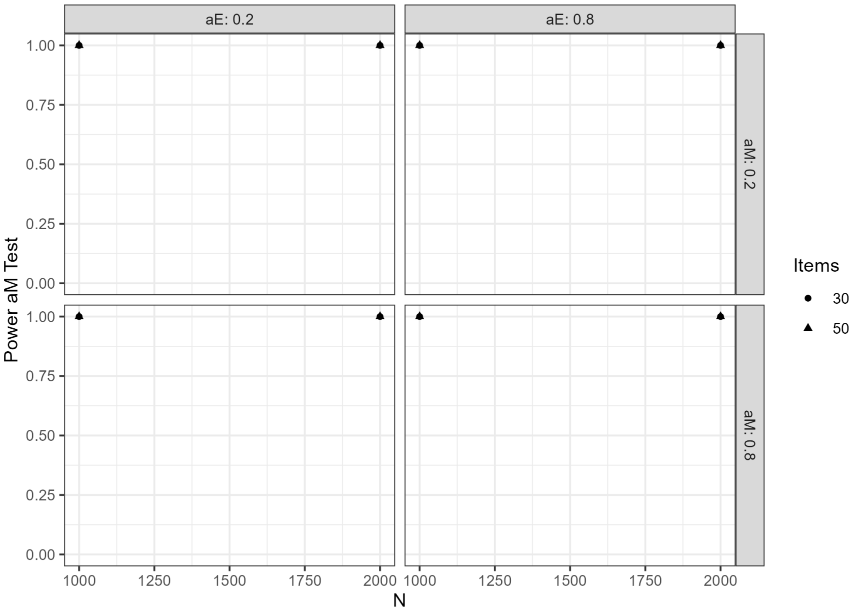

Change of Non-Moderate Response Parameter by -0.2

Figure 9

Type I Error when testing for parameter changes in α^{e} when α^{nm} changed by -0.2.

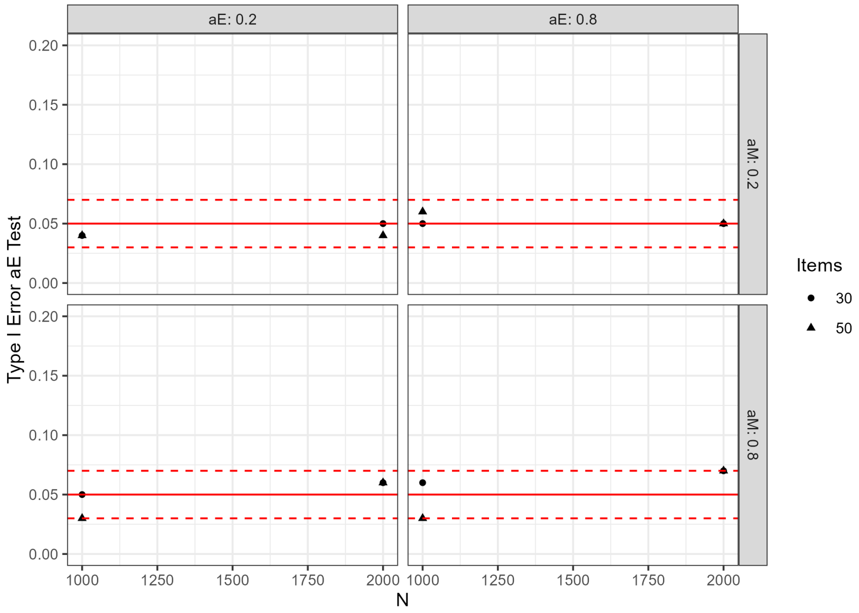

Figure 10

Power when testing for parameter changes in α^{nm} when α^{nm} changed by -0.2.

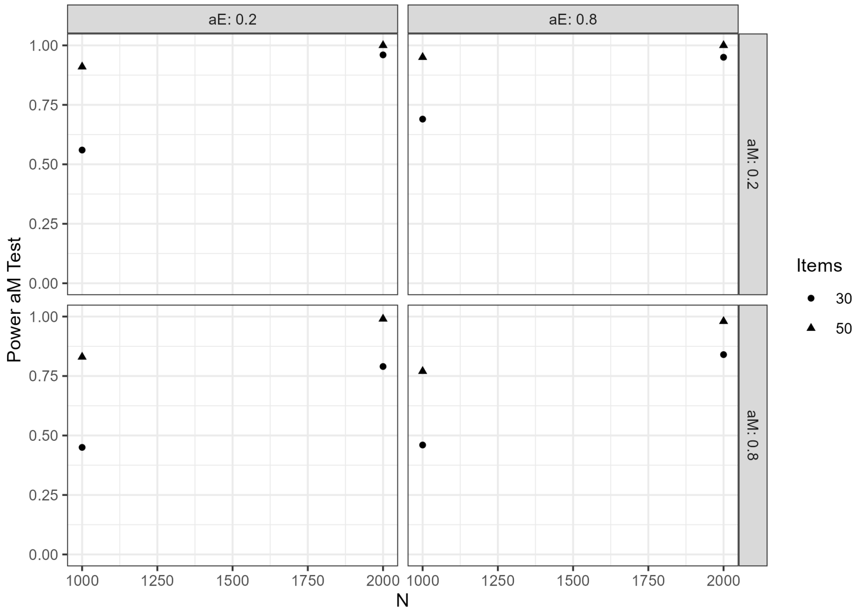

Change of Extreme Response Parameter by +0.5

Figure 11

Power when testing for parameter changes in α^e when α^e changed by +0.5.

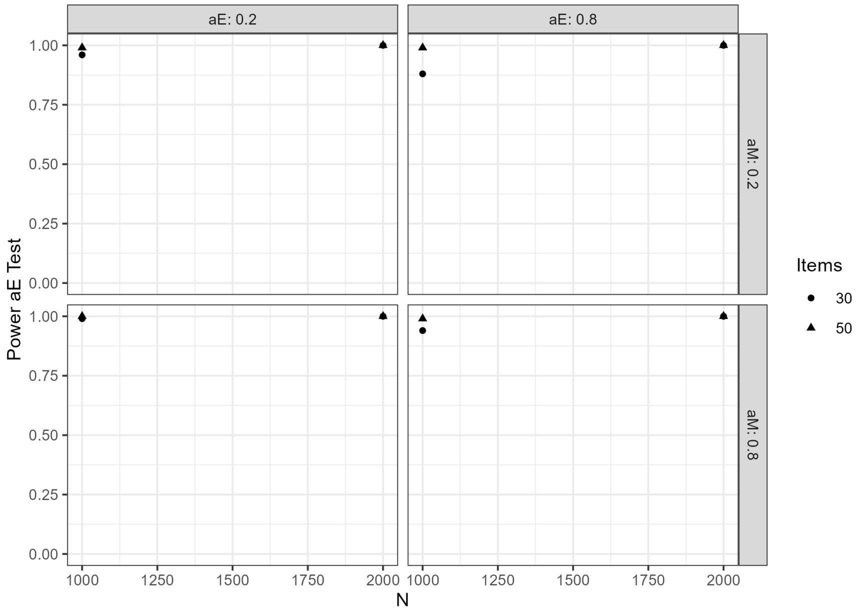

Figure 12

Type I Error when testing for parameter changes in α^{nm} when α^e changed by +0.5.

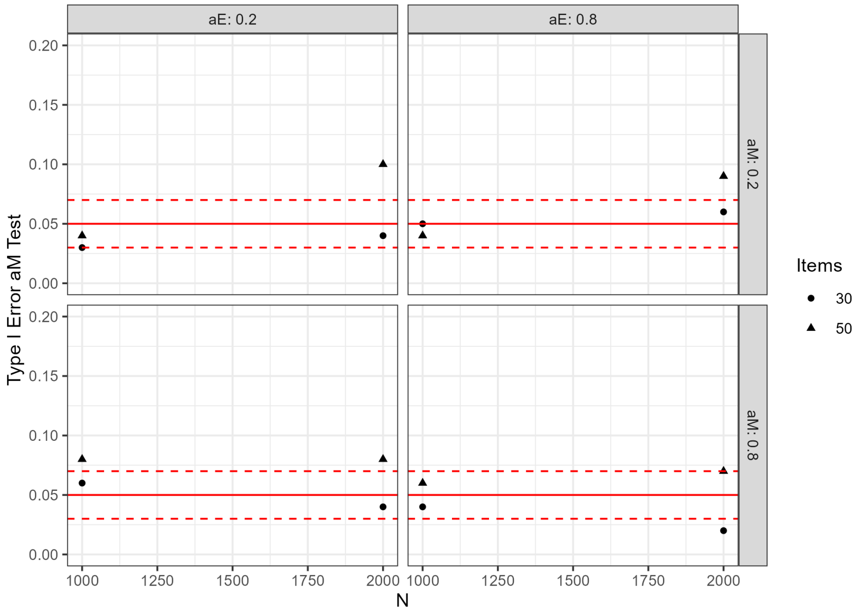

Change of Extreme Response Parameter by +0.2

Figure 13

Power when testing for parameter changes in α^e when α^e changed by +0.2.

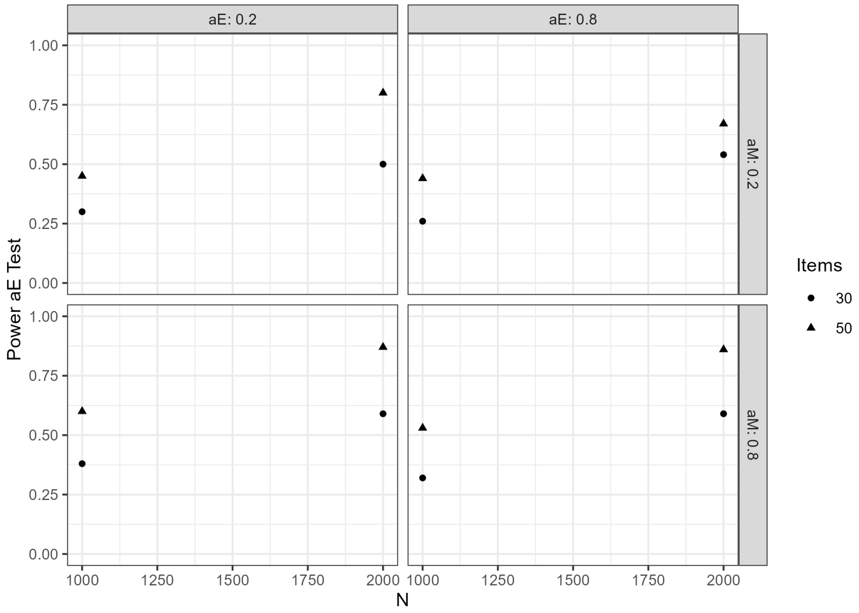

Figure 14

Type I Error when testing for parameter changes in α^{nm} when α^e changed by +0.2.

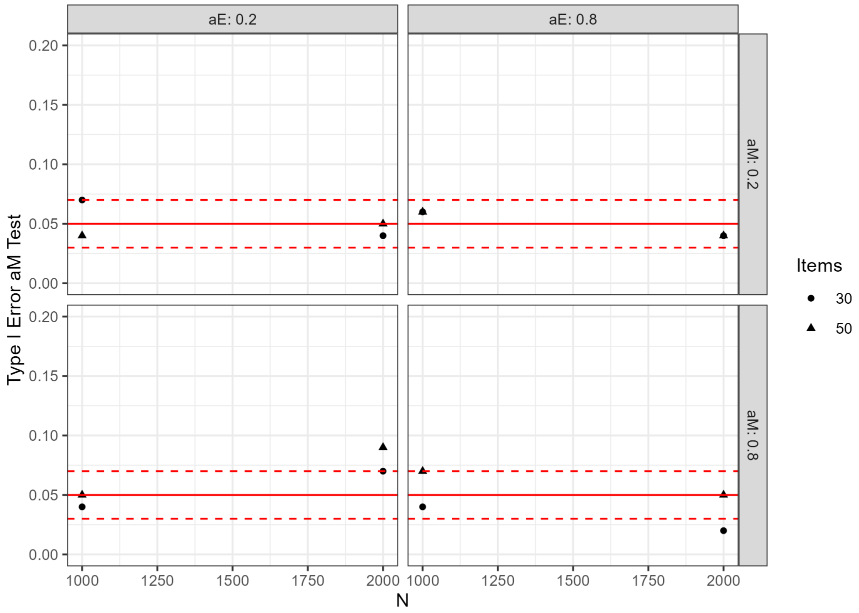

Change of Extreme Response Parameter by -0.5

Figure 15

Power when testing for parameter changes in α^e when α^e changed by -0.5.

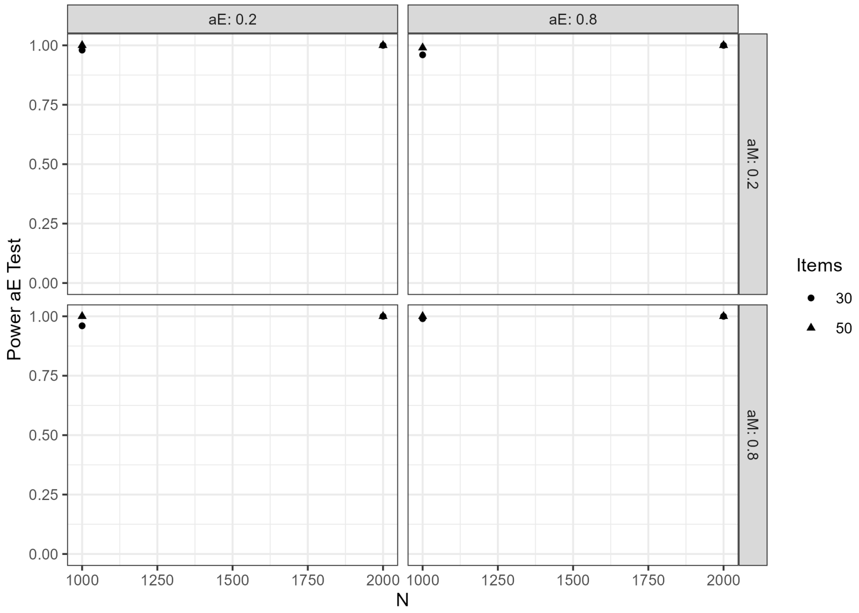

Figure 16

Type I Error when testing for parameter changes in α^{nm} when α^e changed by -0.5.

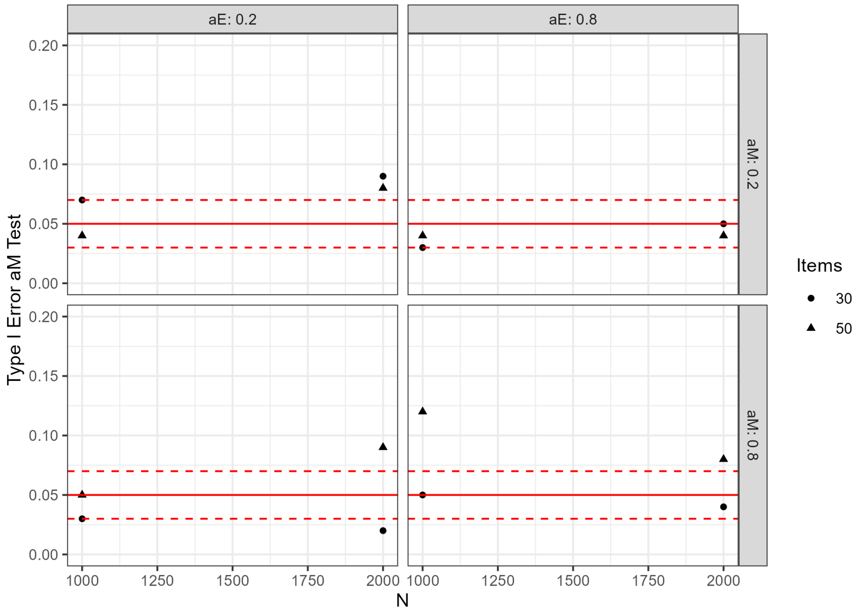

Change of Extreme Response Parameter by -0.2

Figure 17

Power when testing for parameter changes in α^e when α^e changed by -0.2.

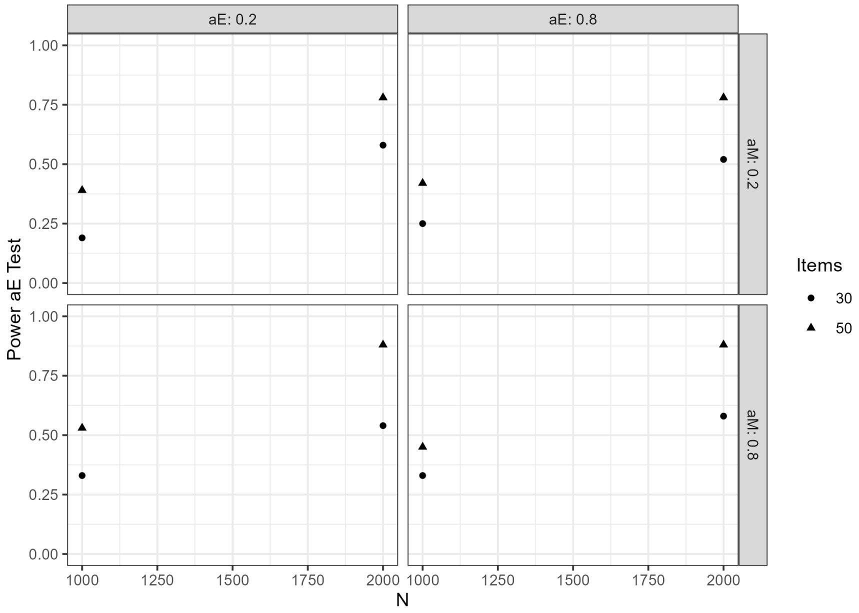

Figure 18

Type I Error when testing for parameter changes in α^{nm} when α^e changed by -0.2.

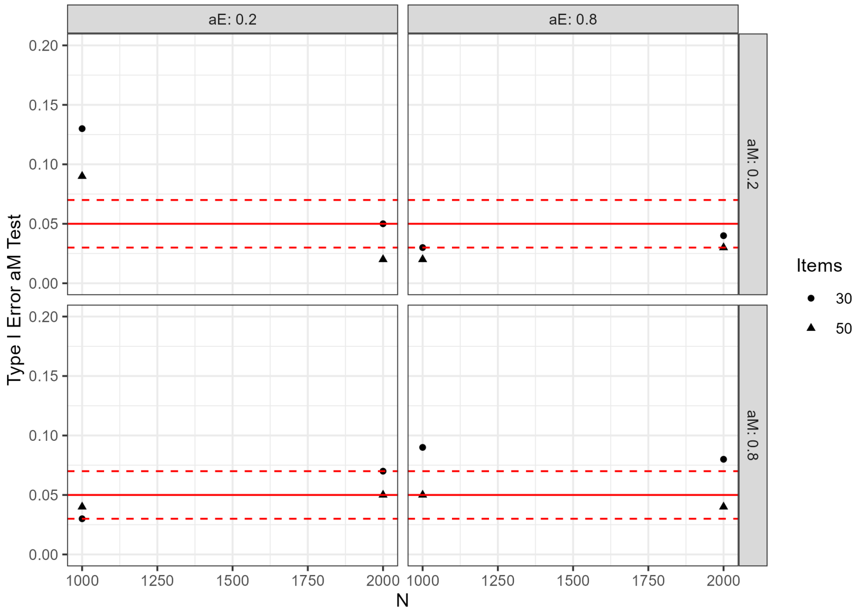

Ordinal Covariate, Test Statistic `WDMo`, Symmetrical Distribution of Covariate

Parameter Invariance

Figure 19

Type I Error when testing for parameter changes in α^e when all parameters are invariant. Red lines indicate the nominal alpha level of 0.05 and an approximate 95% confidence interval for the hit rates.

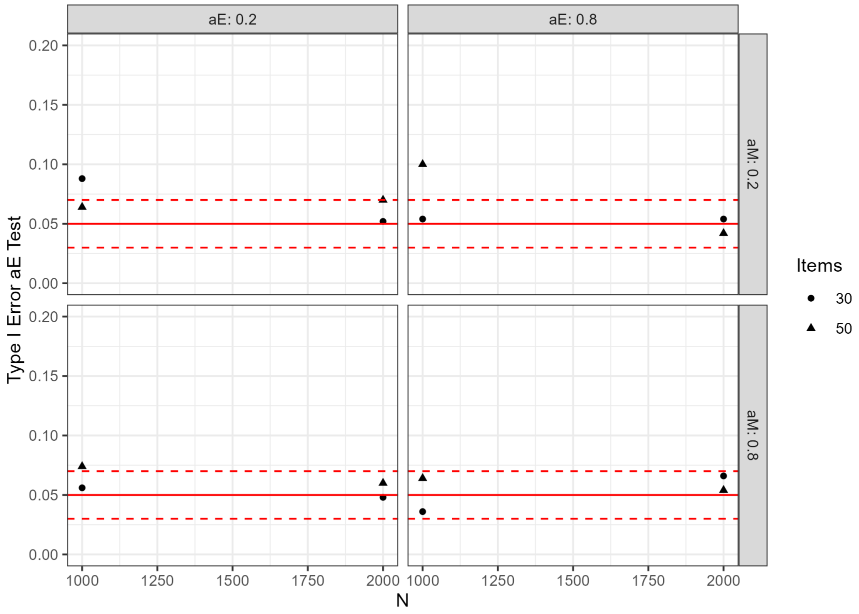

Figure 20

Type I Error when testing for parameter changes in α^{nm} when all parameters are invariant. Red lines indicate the nominal alpha level of 0.05 and an approximate 95% confidence interval for the hit rates.

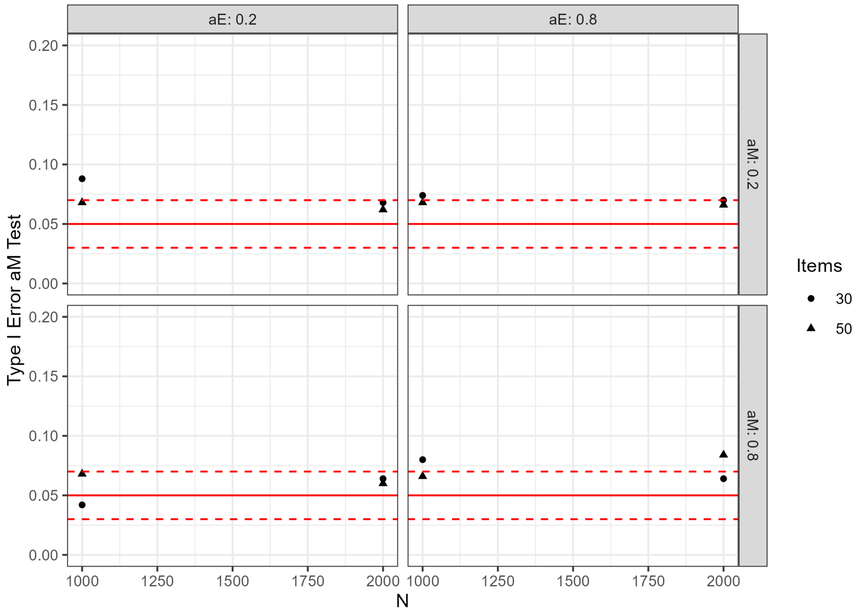

Change of Non-Moderate Response Parameter by +0.5

Figure 21

Type I Error when testing for parameter changes in α^e when α^{nm} changed by +0.5. Red lines indicate the nominal alpha level of 0.05 and an approximate 95% confidence interval for the hit rates.

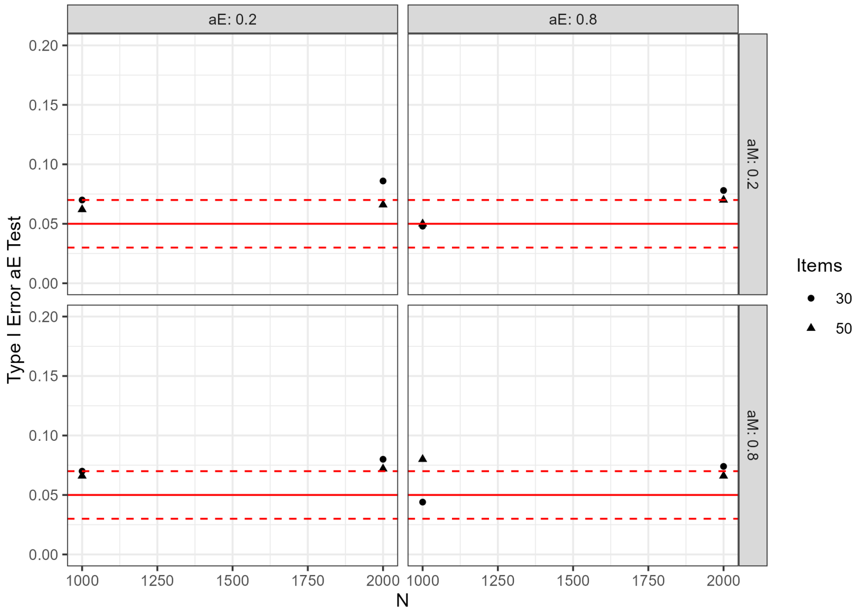

Figure 22

Power when testing for parameter changes in α^{nm} when α^{nm} changed by +0.5.

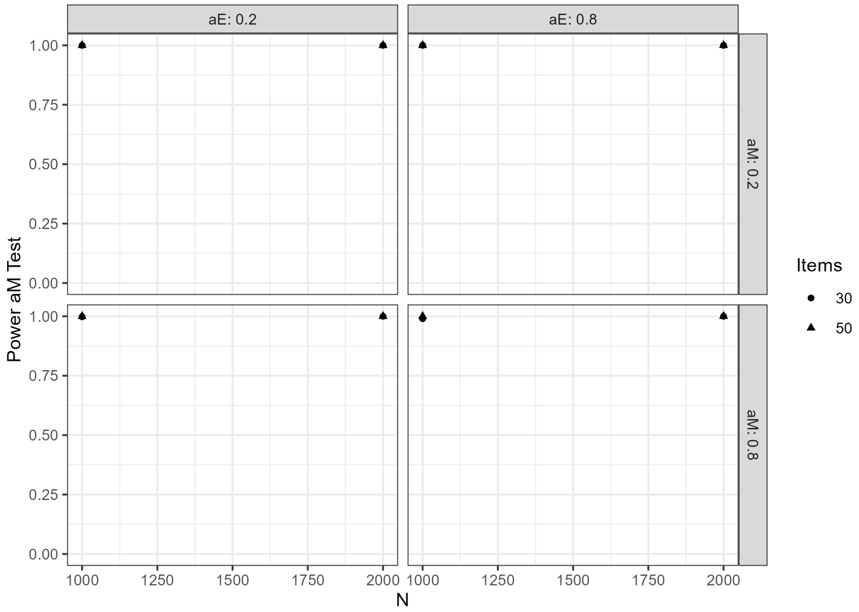

Change of Non-Moderate Response Parameter by +0.2

Figure 23

Type I Error when testing for parameter changes in α^e when α^{nm} changed by +0.2. Red lines indicate the nominal alpha level of 0.05 and an approximate 95% confidence interval for the hit rates.

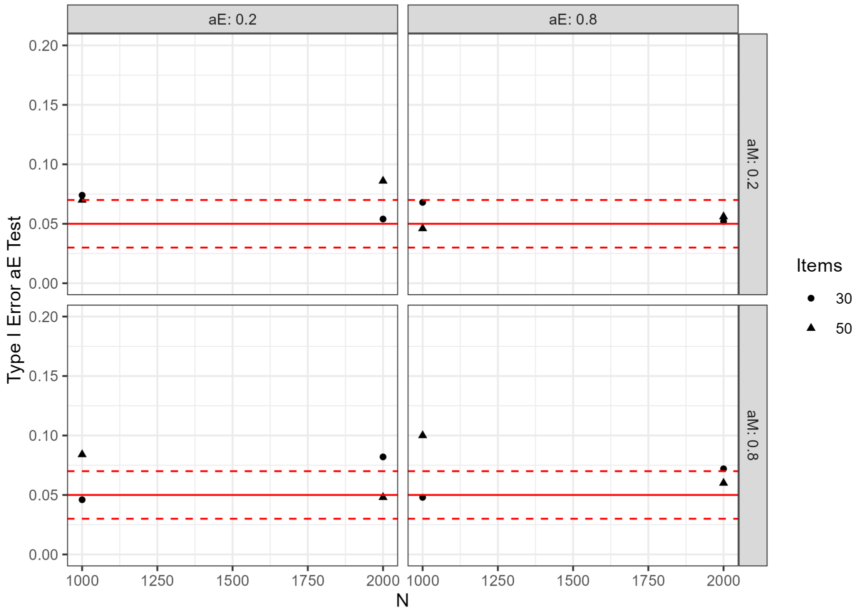

Figure 24

Power when testing for parameter changes in α^{nm} when α^{nm} changed by +0.2.

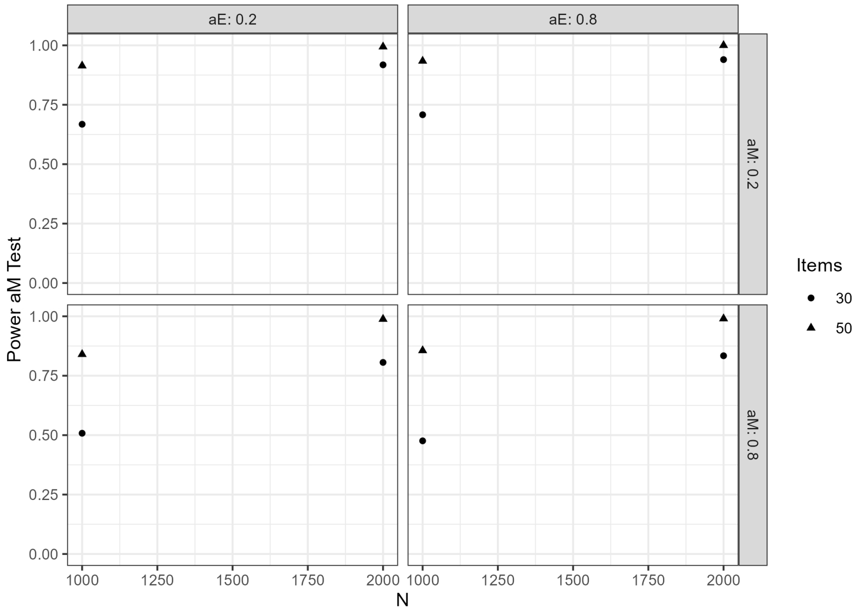

Change of Non-Moderate Response Parameter by -0.5

Figure 25

Type I Error when testing for parameter changes in α^e when α^{nm} changed by -0.5. Red lines indicate the nominal alpha level of 0.05 and an approximate 95% confidence interval for the hit rates.

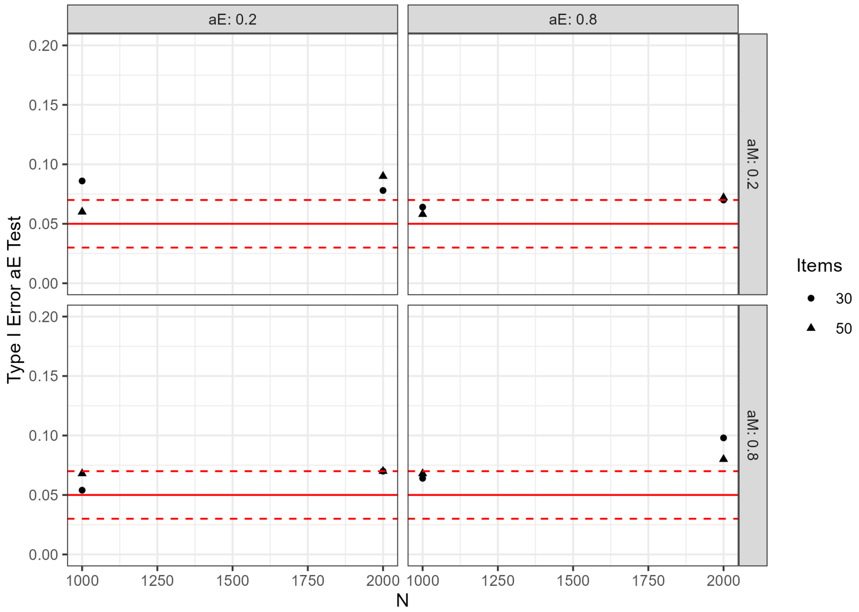

Figure 26

Power when testing for parameter changes in α^{nm} when α^{nm} changed by -0.5.

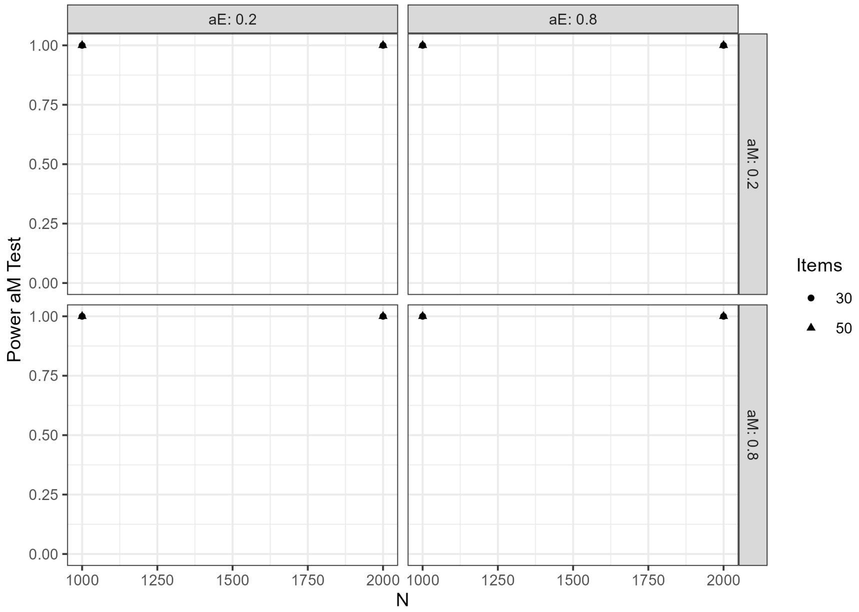

Change of Non-Moderate Response Parameter by -0.2

Figure 27

Type I Error when testing for parameter changes in α^e when α^{nm} changed by -0.2. Red lines indicate the nominal alpha level of 0.05 and an approximate 95% confidence interval for the hit rates.

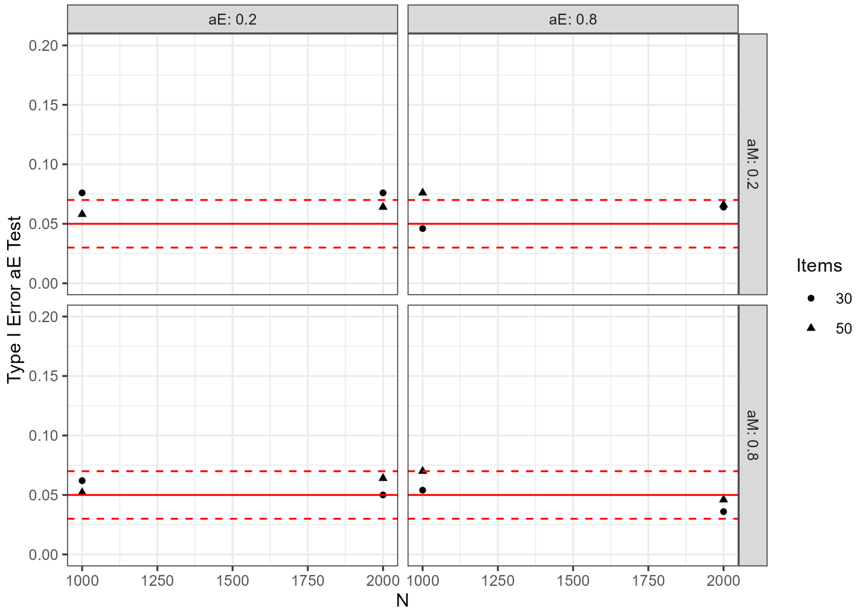

Figure 28

Power when testing for parameter changes in α^{nm} when α^{nm} changed by -0.2.

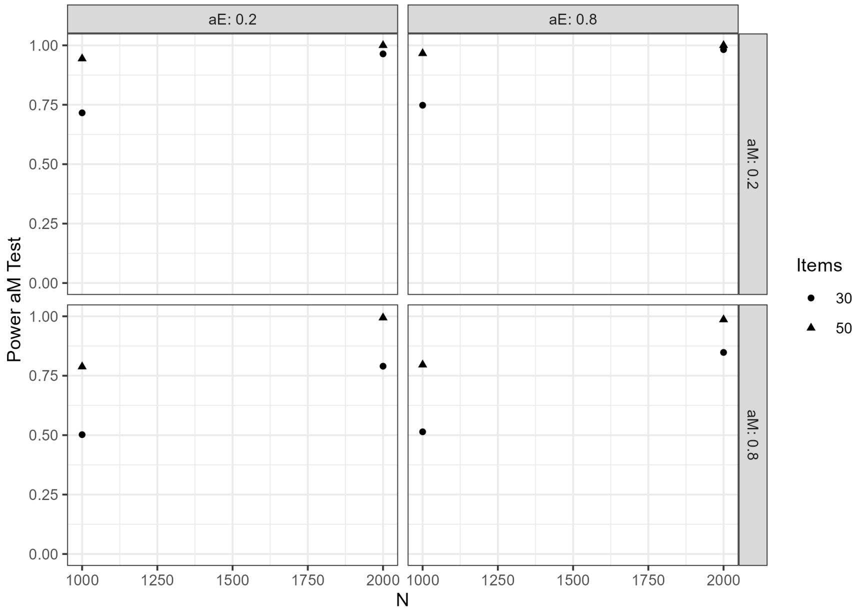

Change of Extreme Response Parameter by +0.5

Figure 29

Power when testing for parameter changes in α^e when α^e changed by +0.5.

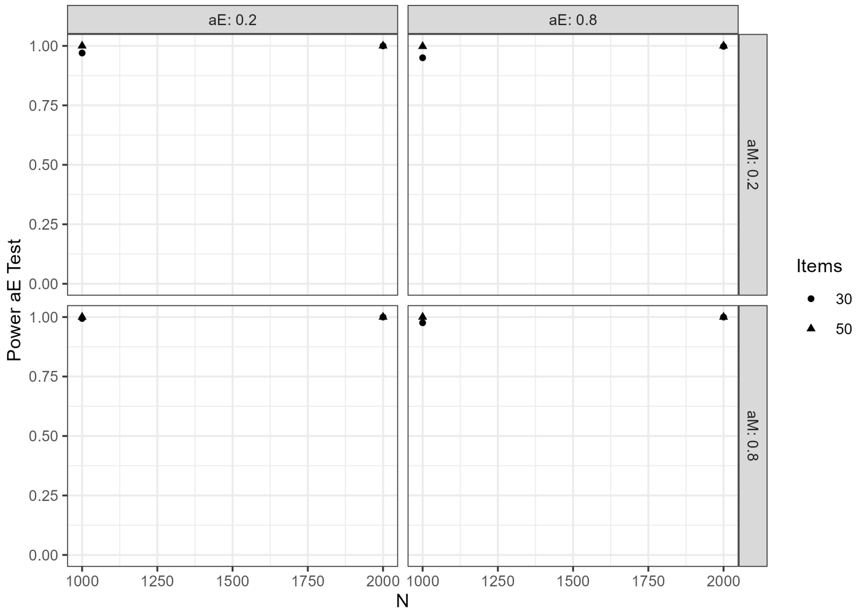

Figure 30

Type I Error when testing for parameter changes in α^{nm} when α^e changed by +0.5. Red lines indicate the nominal alpha level of 0.05 and an approximate 95% confidence interval for the hit rates.

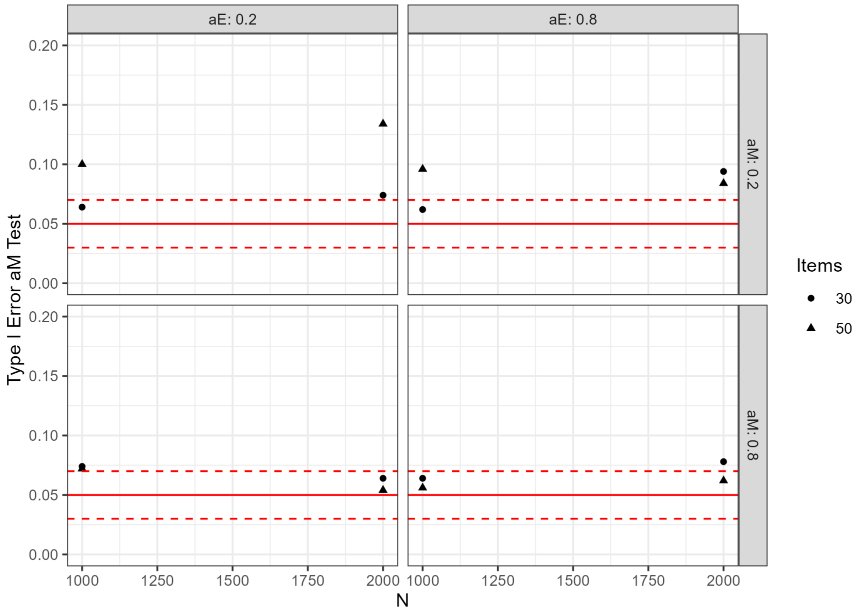

Change of Extreme Response Parameter by +0.2

Figure 31

Power when testing for parameter changes in α^e when α^e changed by +0.2.

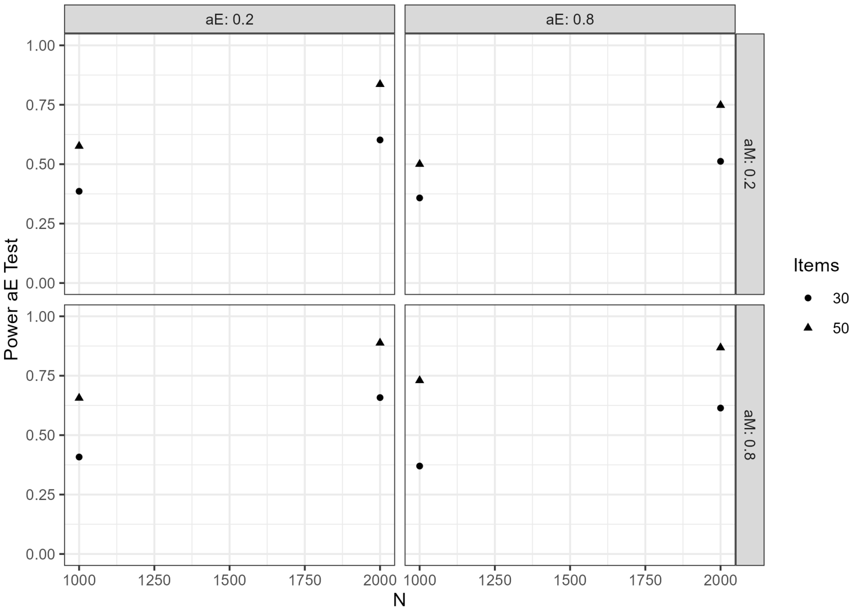

Figure 32

Type I Error when testing for parameter changes in α^{nm} when α^e changed by +0.2. Red lines indicate the nominal alpha level of 0.05 and an approximate 95% confidence interval for the hit rates.

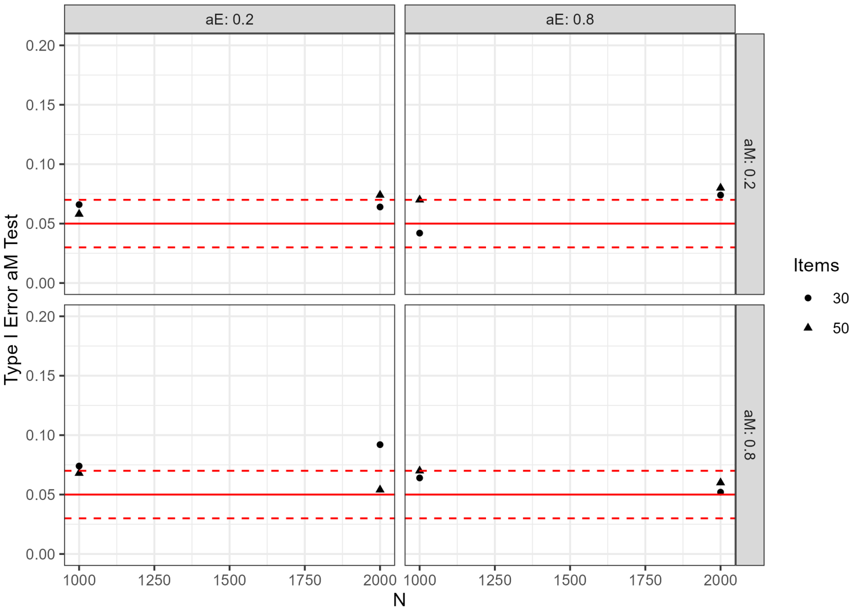

Change of Extreme Response Parameter by -0.5

Figure 33

Power when testing for parameter changes in α^e when α^e changed by -0.5.

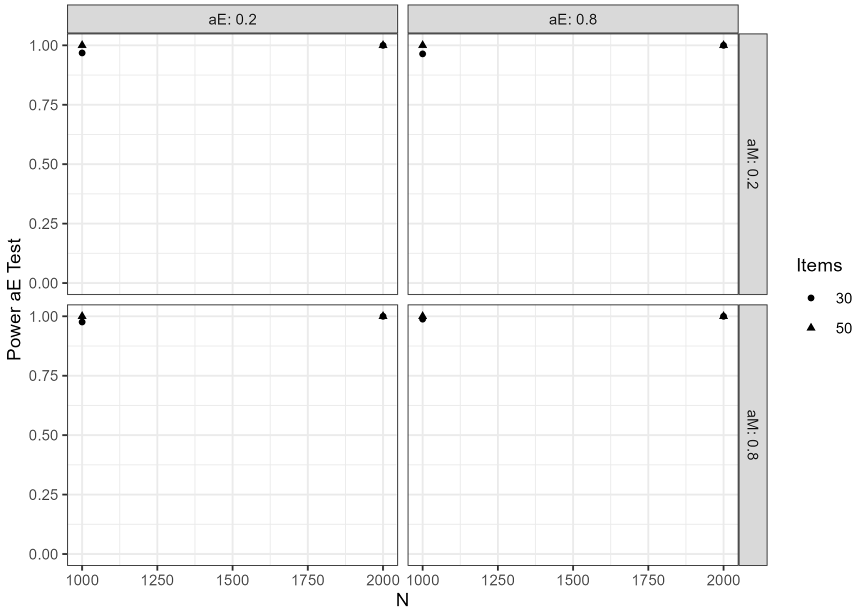

Figure 34

Type I Error when testing for parameter changes in α^{nm} when α^e changed by -0.5. Red lines indicate the nominal alpha level of 0.05 and an approximate 95% confidence interval for the hit rates.

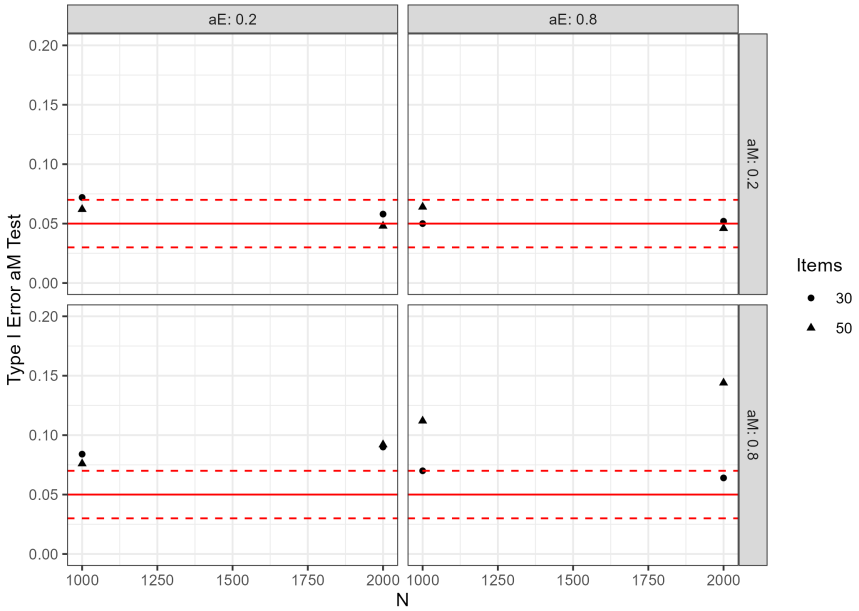

Change of Extreme Response Parameter by -0.2

Figure 35

Power when testing for parameter changes in α^e when α^e changed by -0.2.

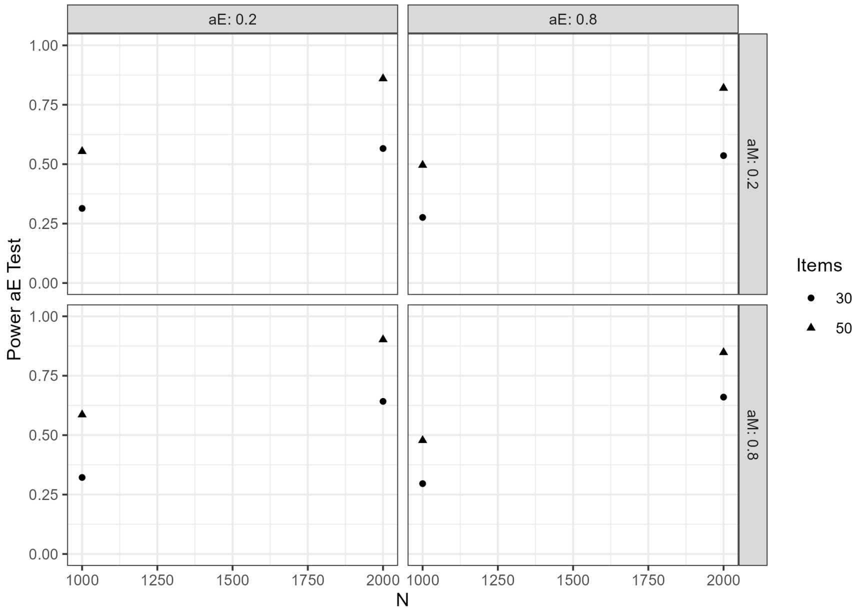

Figure 36

Type I Error when testing for parameter changes in α^{nm} when α^e changed by -0.2. Red lines indicate the nominal alpha level of 0.05 and an approximate 95% confidence interval for the hit rates.

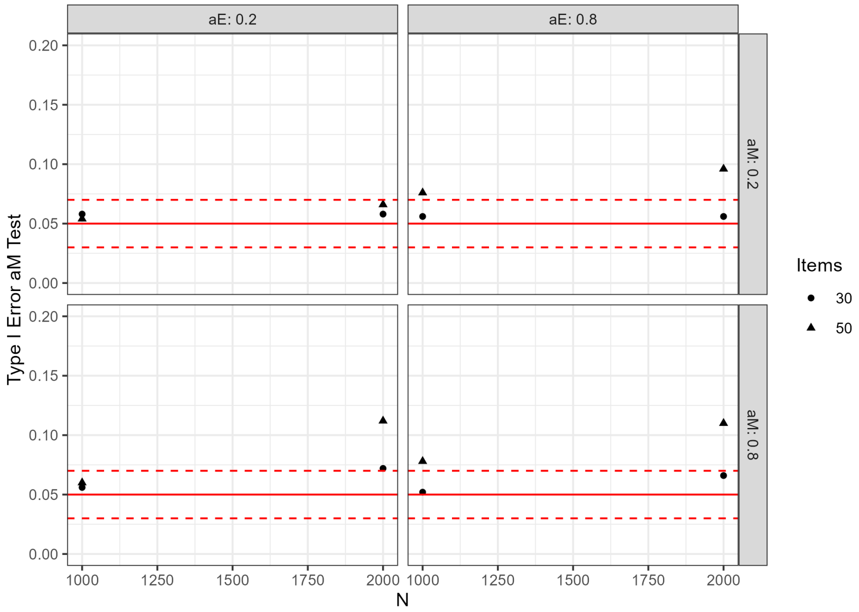

Ordinal Covariate, Test Statistic WDMo, Skewed Distribution of Covariate

Parameter Invariance

Figure 37

Type I Error when testing for parameter changes in α^e when all parameters are invariant. Red lines indicate the nominal alpha level of 0.05 and an approximate 95% confidence interval for the hit rates.

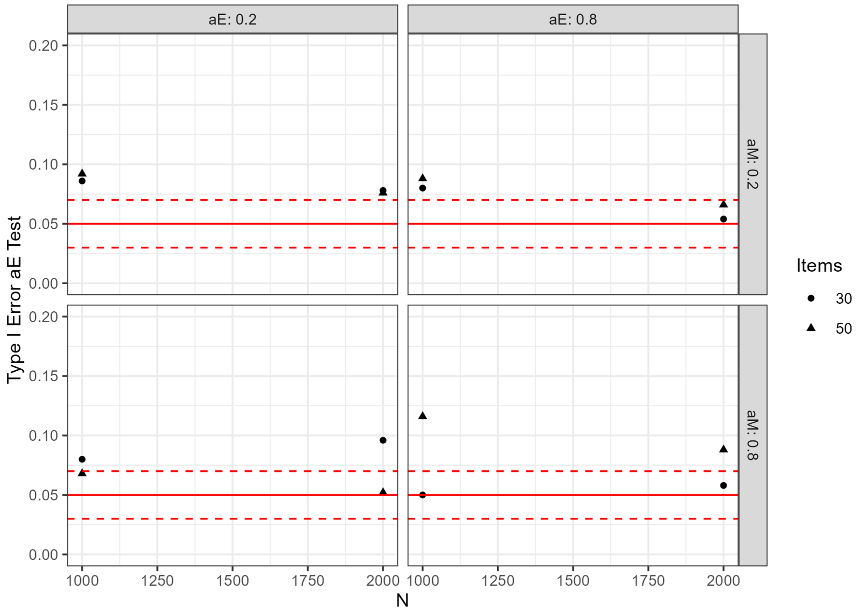

Figure 38

Type I Error when testing for parameter changes in α^{nm} when all parameters are invariant. Red lines indicate the nominal alpha level of 0.05 and an approximate 95% confidence interval for the hit rates.

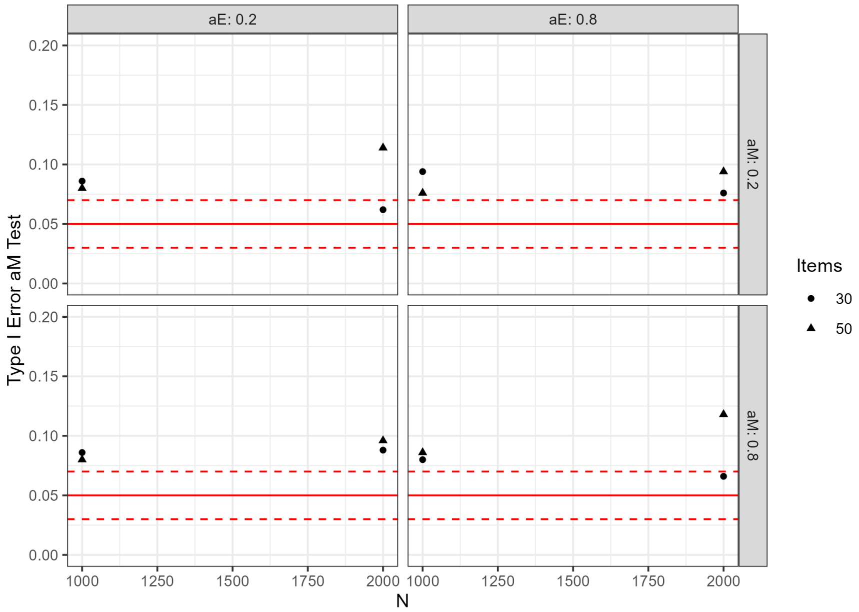

Change of Non-Moderate Response Parameter by +0.5

Figure 39

Type I Error when testing for parameter changes in α^e when α^{nm} changed by +0.5. Red lines indicate the nominal alpha level of 0.05 and an approximate 95% confidence interval for the hit rates.

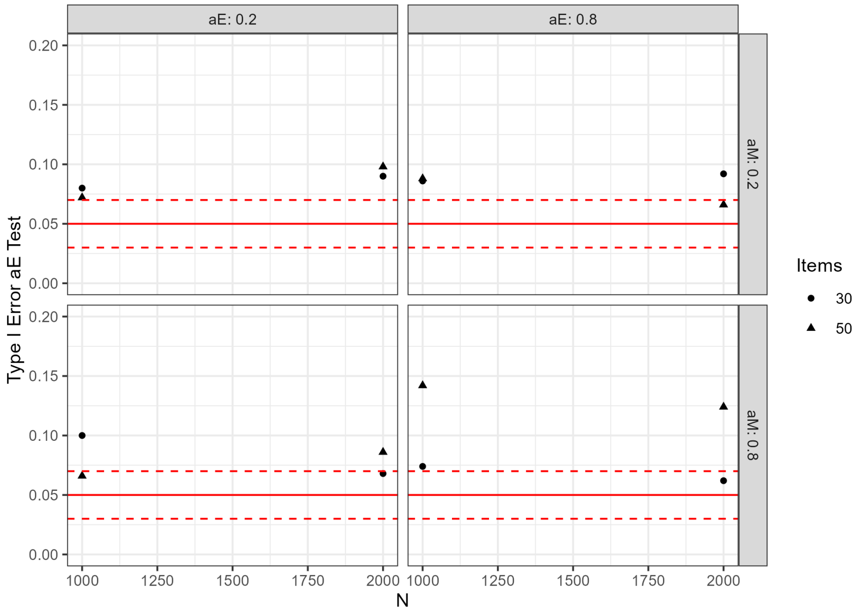

Figure 40

Power when testing for parameter changes in α^{nm} when α^{nm} changed by +0.5.

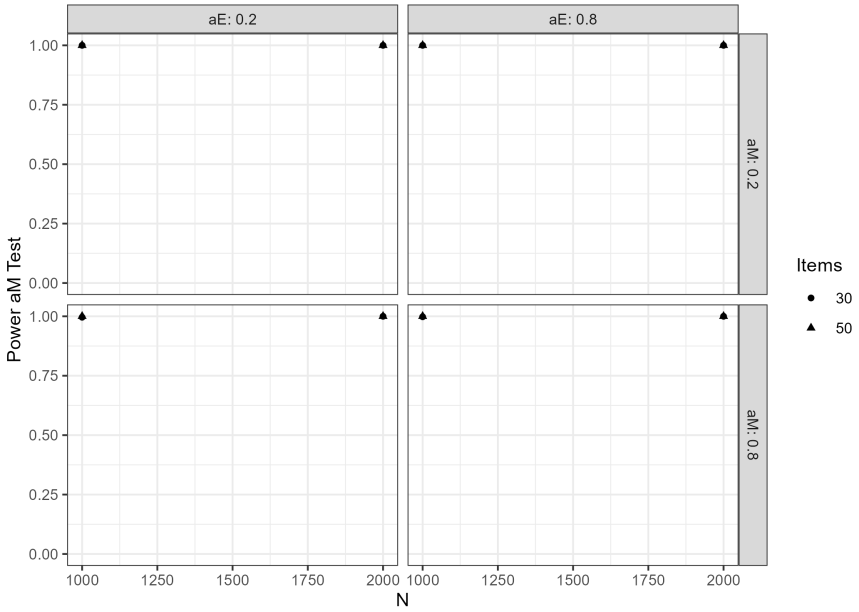

Change of Non-Moderate Response Parameter by +0.2

Figure 41

Type I Error when testing for parameter changes in α^e when α^{nm} changed by +0.2. Red lines indicate the nominal alpha level of 0.05 and an approximate 95% confidence interval for the hit rates.

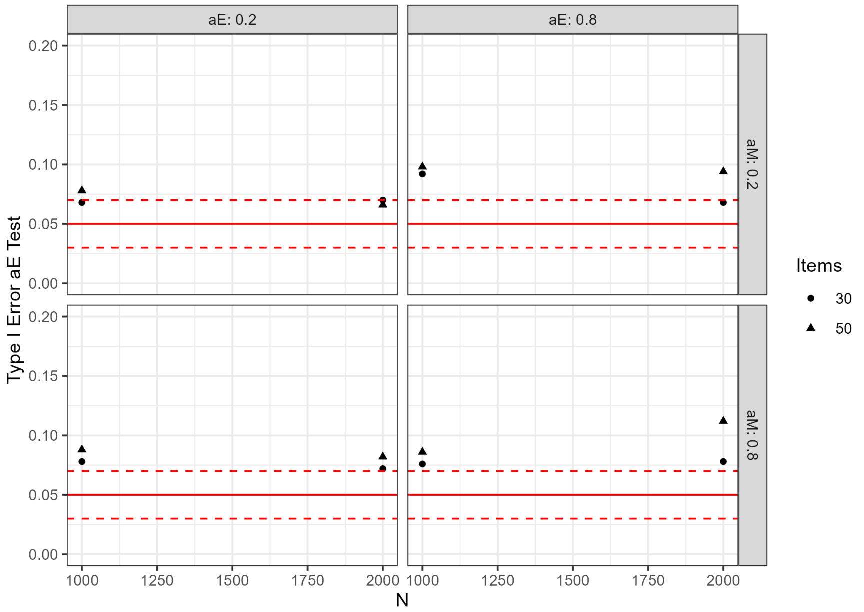

Figure 42

Power when testing for parameter changes in α^{nm} when α^{nm} changed by +0.2.

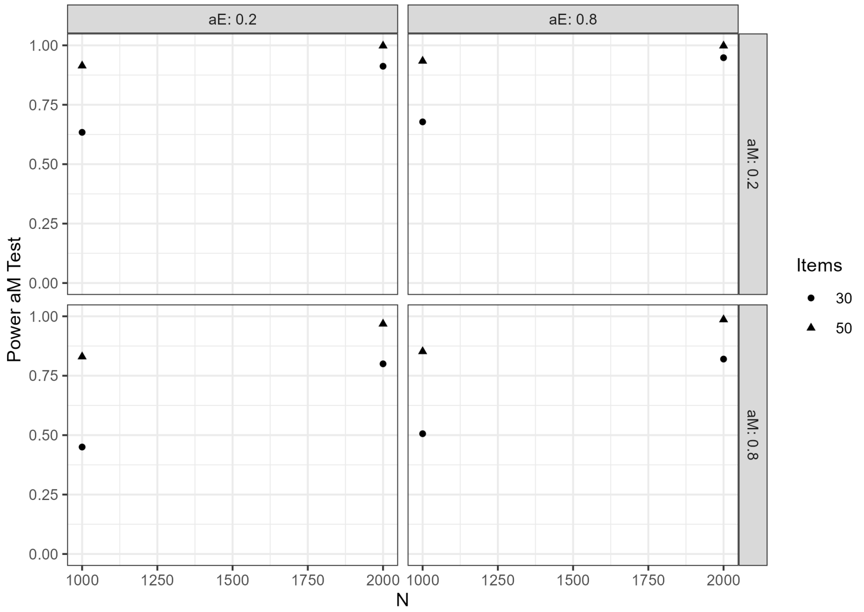

Change of Non-Moderate Response Parameter by -0.5

Figure 43

Type I Error when testing for parameter changes in α^e when α^{nm} changed by -0.5. Red lines indicate the nominal alpha level of 0.05 and an approximate 95% confidence interval for the hit rates.

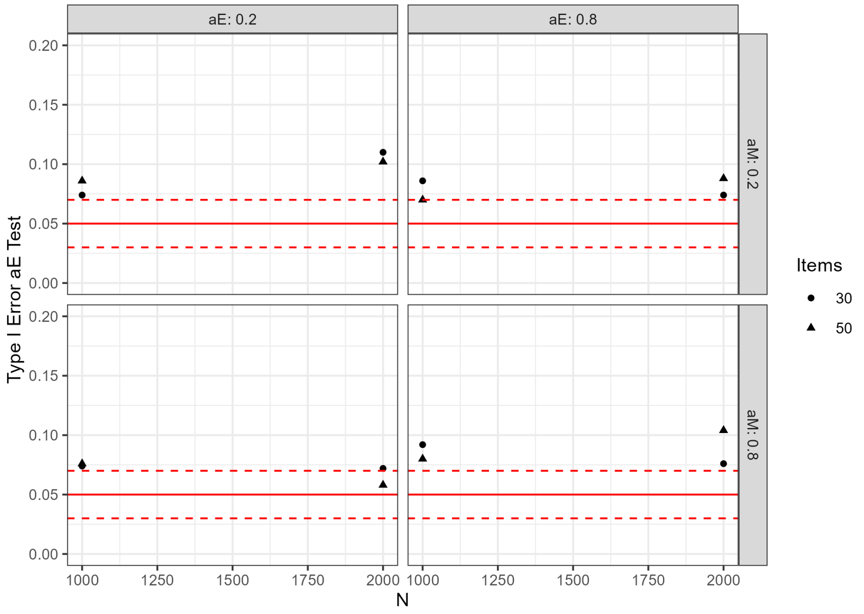

Figure 44

Power when testing for parameter changes in α^{nm} when α^{nm} changed by -0.5.

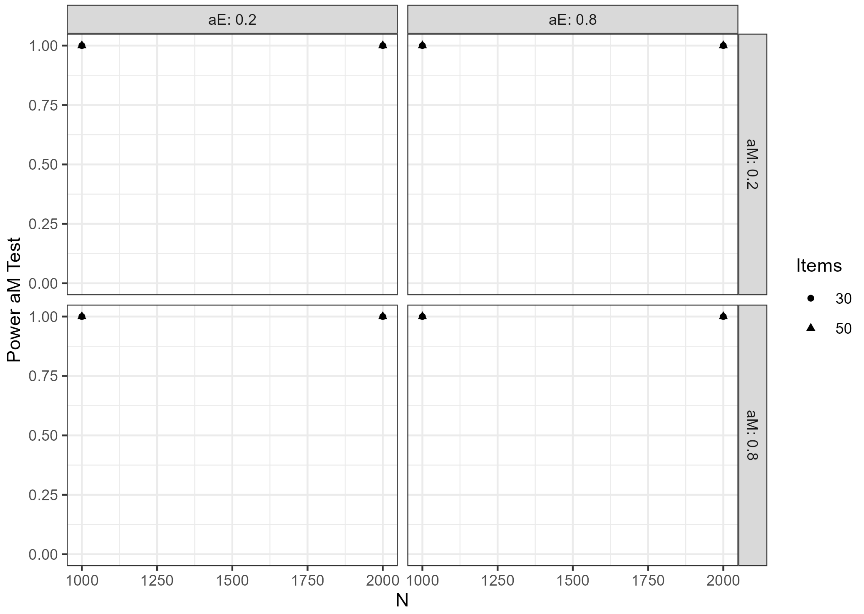

Change of Non-Moderate Response Parameter by -0.2

Figure 45

Type I Error when testing for parameter changes in α^e when α^{nm} changed by -0.2. Red lines indicate the nominal alpha level of 0.05 and an approximate 95% confidence interval for the hit rates.

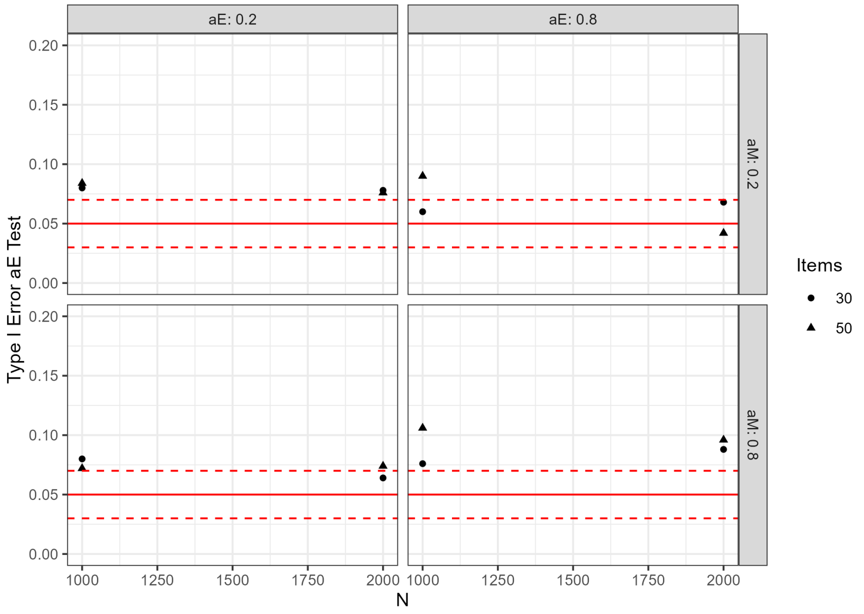

Figure 46

Power when testing for parameter changes in α^{nm} when α^{nm} changed by -0.2.

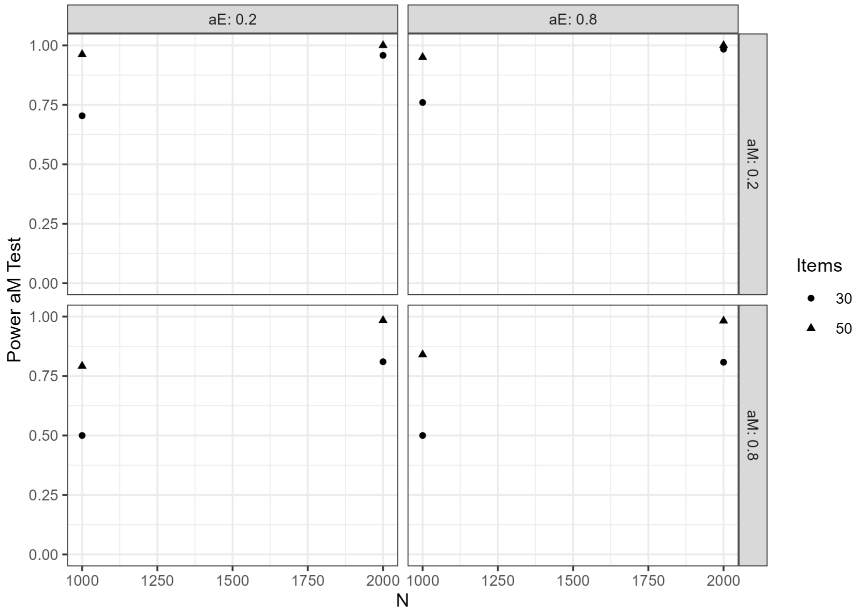

Change of Extreme Response Parameter by +0.5

Figure 47

Power when testing for parameter changes in α^e when α^e changed by +0.5.

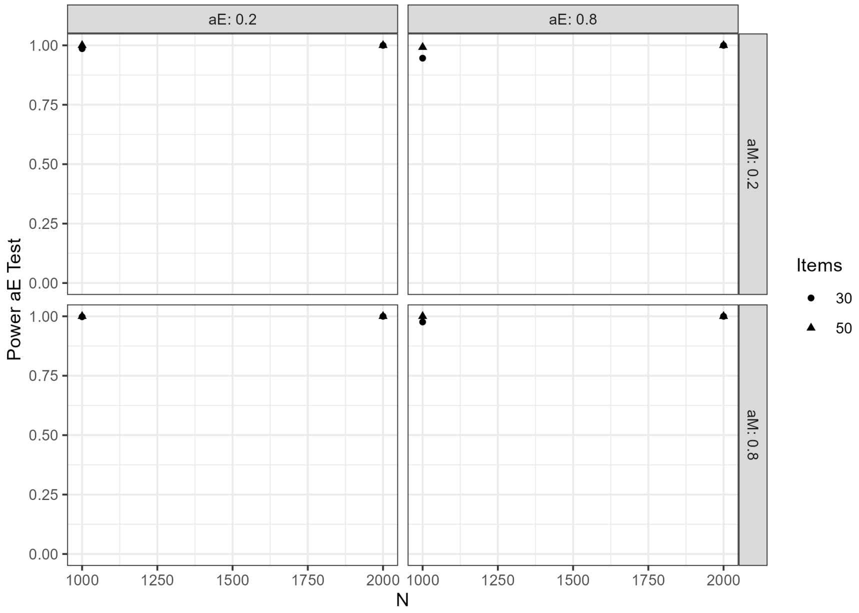

Figure 48

Type I Error when testing for parameter changes in α^{nm} when α^e changed by +0.5. Red lines indicate the nominal alpha level of 0.05 and an approximate 95% confidence interval for the hit rates.

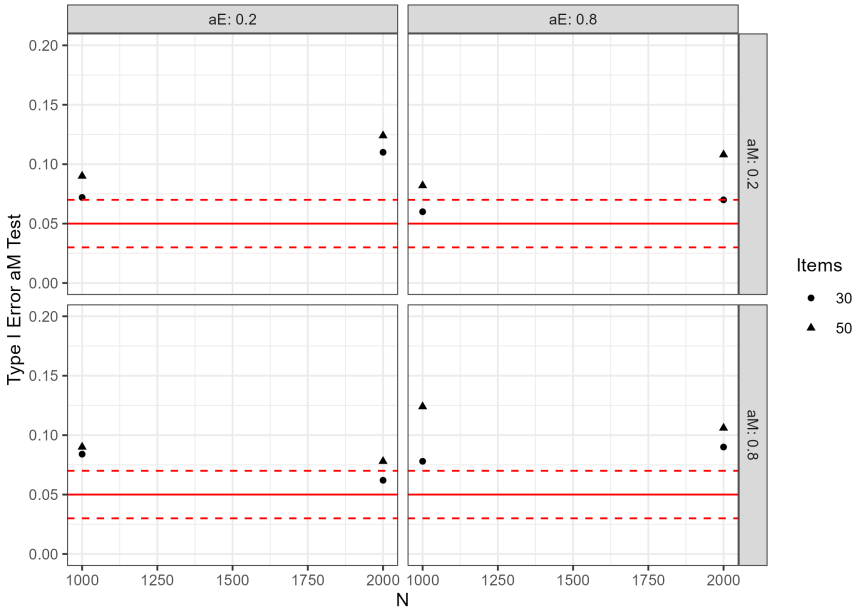

Change of Extreme Response Parameter by +0.2

Figure 49

Power when testing for parameter changes in α^e when α^e changed by +0.2.

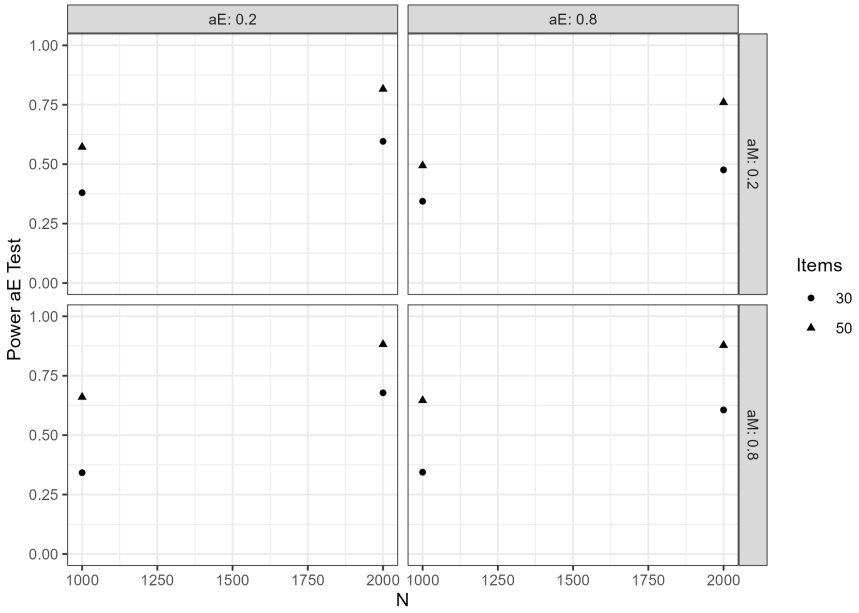

Figure 50

Type I Error when testing for parameter changes in α^{nm} when α^e changed by +0.2. Red lines indicate the nominal alpha level of 0.05 and an approximate 95% confidence interval for the hit rates.

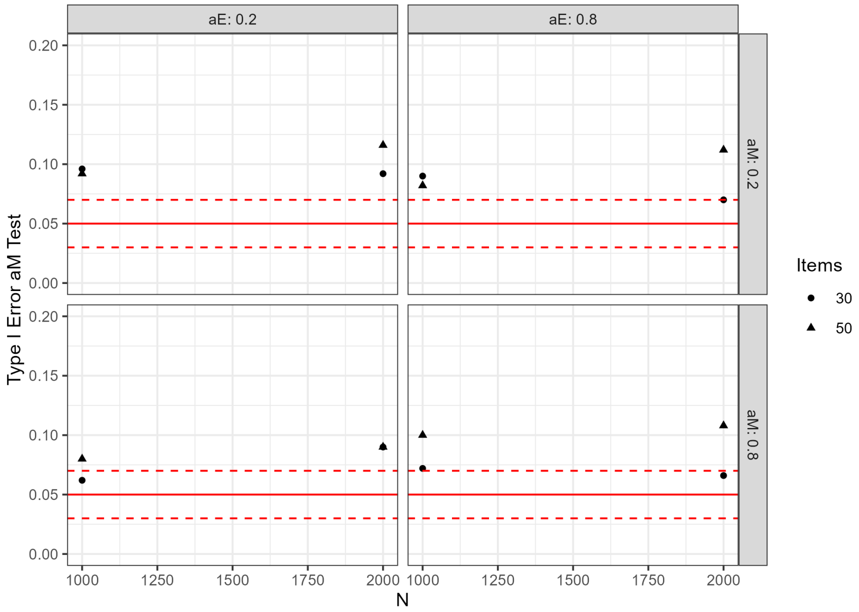

Change of Extreme Response Parameter by -0.5

Figure 51

Power when testing for parameter changes in α^e when α^e changed by -0.5.

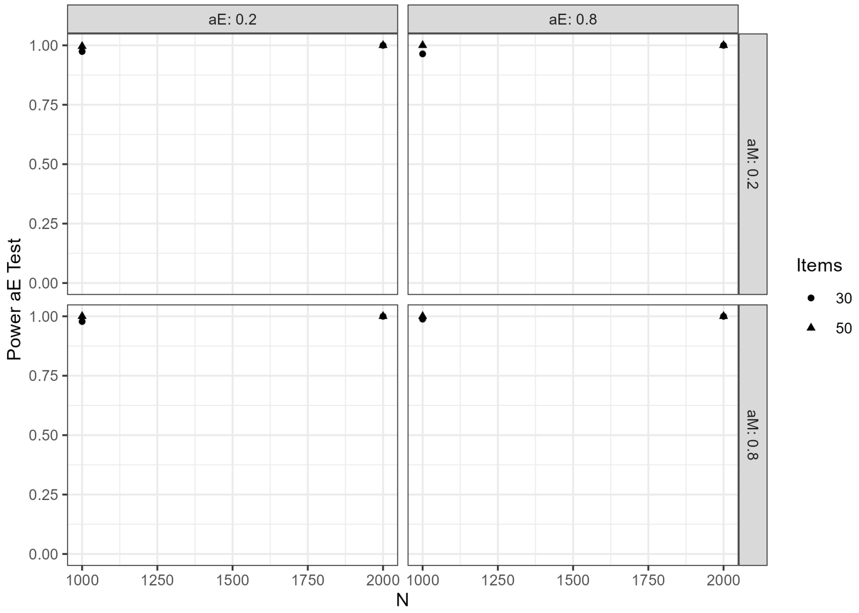

Figure 52

Type I Error when testing for parameter changes in α^{nm} when α^e changed by -0.5. Red lines indicate the nominal alpha level of 0.05 and an approximate 95% confidence interval for the hit rates.

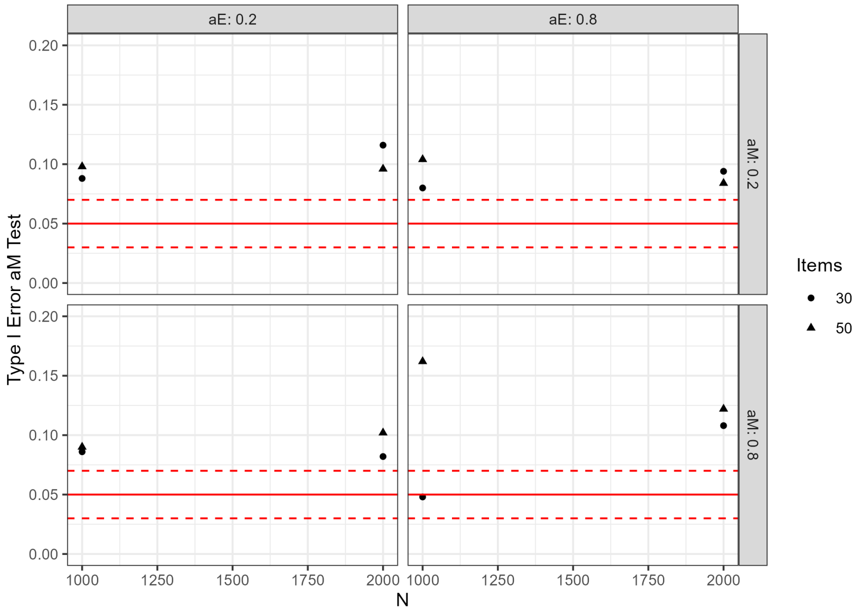

Change of Extreme Response Parameter by -0.2

Figure 53

Power when testing for parameter changes in α^e when α^e changed by -0.2.

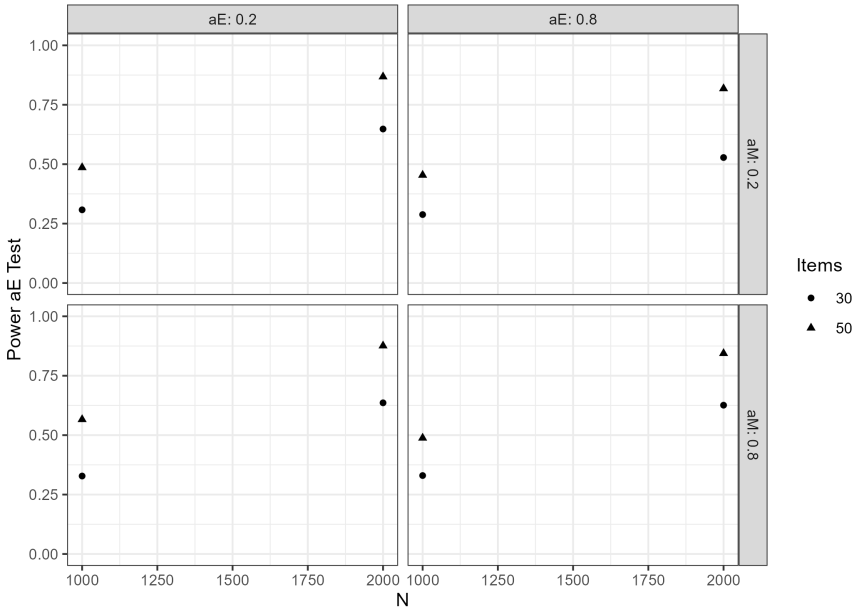

Figure 54

Type I Error when testing for parameter changes in α^{nm} when α^e changed by -0.2. Red lines indicate the nominal alpha level of 0.05 and an approximate 95% confidence interval for the hit rates.

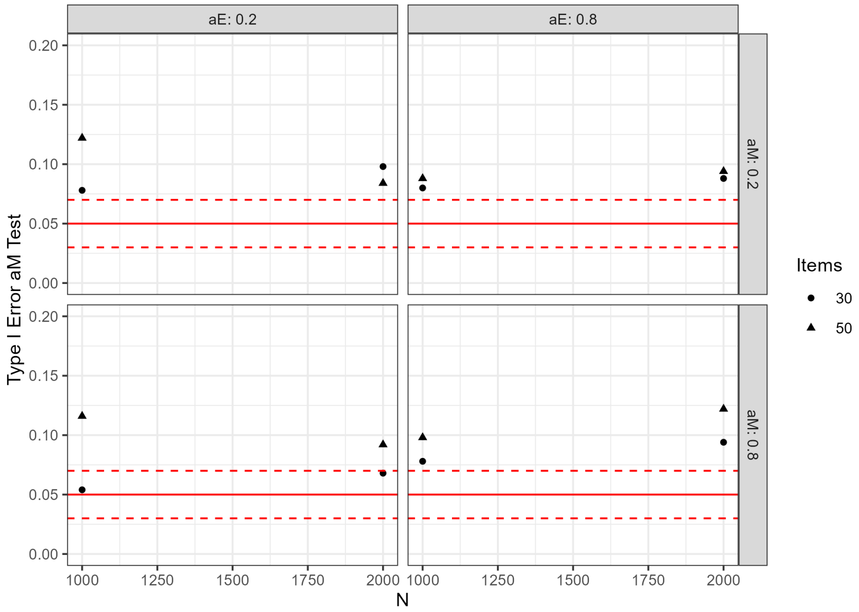

Ordinal Covariate, Test Statistic maxLMo, Symmetrical Distribution of Covariate

Parameter Invariance

Figure 55

Type I Error when testing for parameter changes in α^e when all parameters are invariant. Red lines indicate the nominal alpha level of 0.05 and an approximate 95% confidence interval for the hit rates.

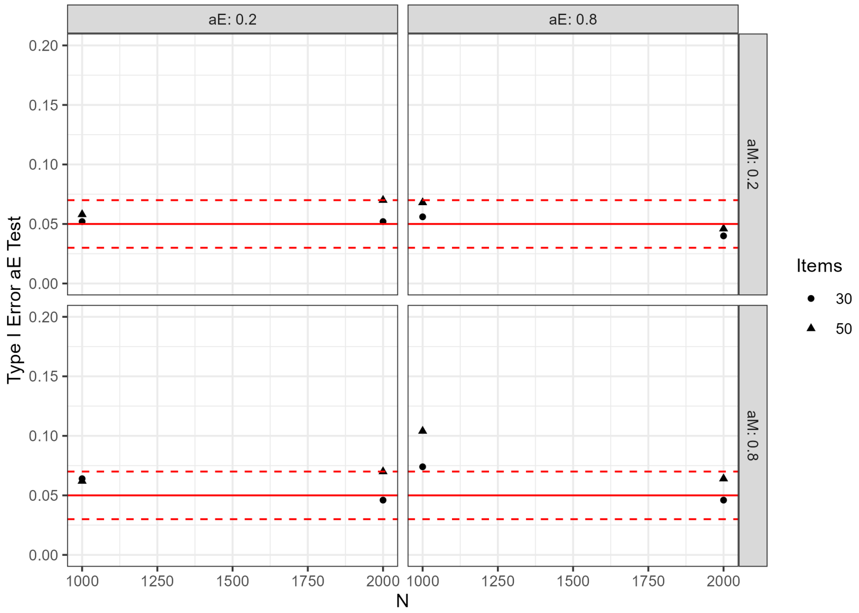

Figure 56

Type I Error when testing for parameter changes in α^{nm} when all parameters are invariant. Red lines indicate the nominal alpha level of 0.05 and an approximate 95% confidence interval for the hit rates.

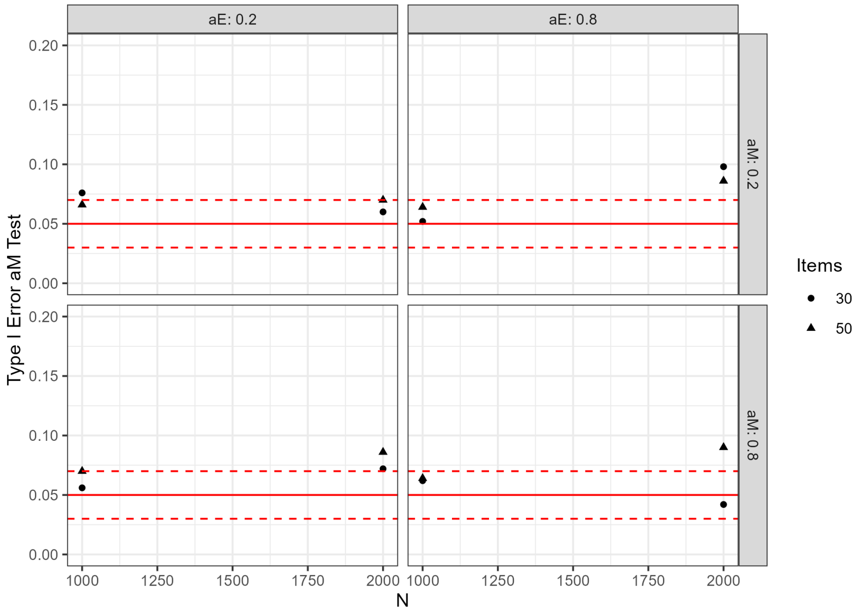

Change of Non-Moderate Response Parameter by +0.5

Figure 57

Type I Error when testing for parameter changes in α^e when α^{nm} changed by +0.5. Red lines indicate the nominal alpha level of 0.05 and an approximate 95% confidence interval for the hit rates.

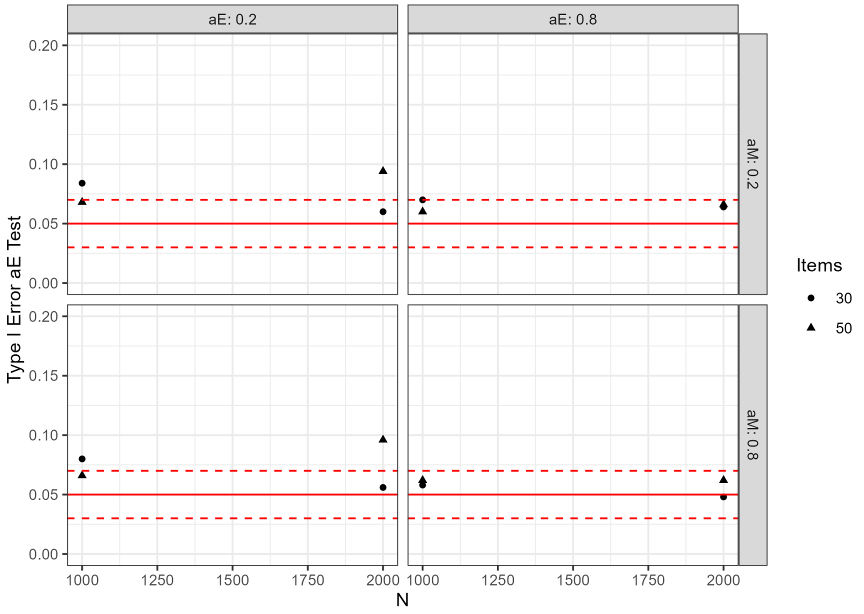

Figure 58

Power when testing for parameter changes in α^{nm} when α^{nm} changed by +0.5.

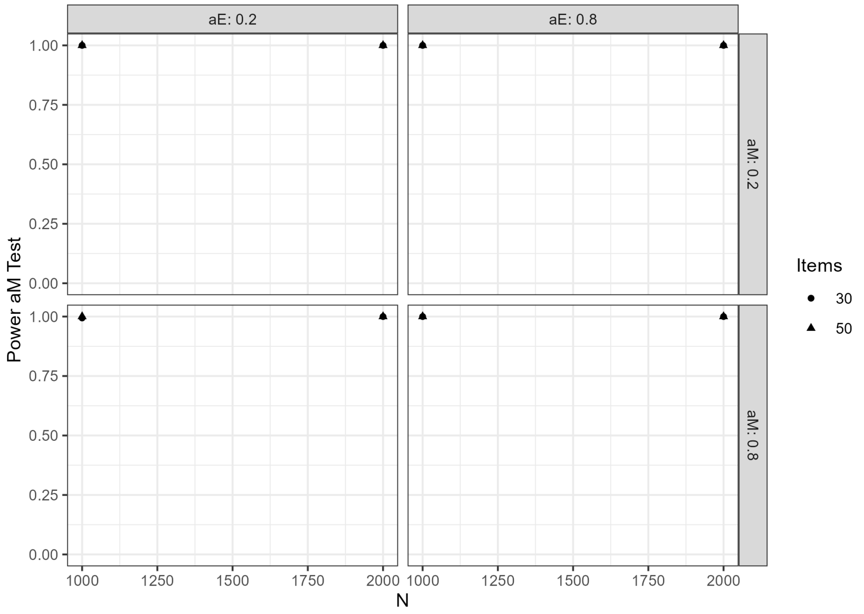

Change of Non-Moderate Response Parameter by +0.2

Figure 59

Type I Error when testing for parameter changes in α^e when α^{nm} changed by +0.2. Red lines indicate the nominal alpha level of 0.05 and an approximate 95% confidence interval for the hit rates.

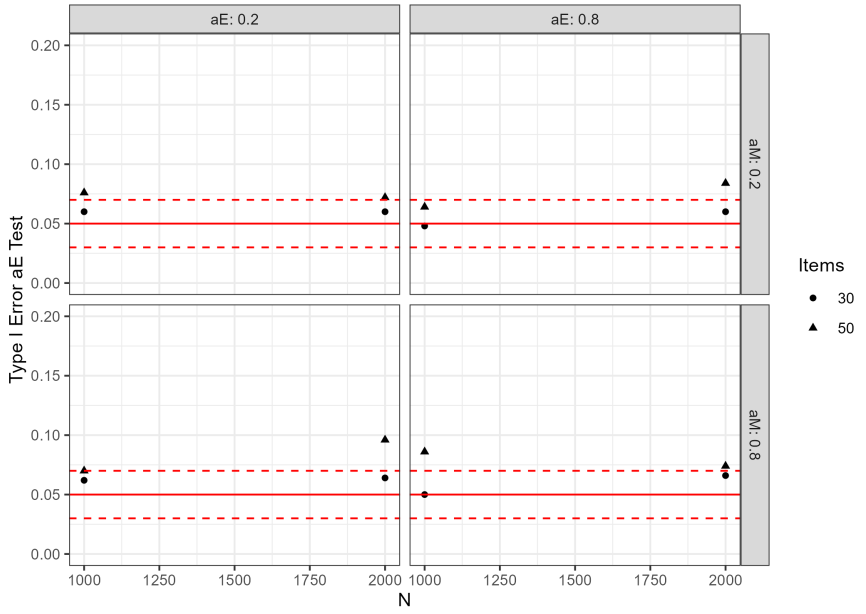

Figure 60

Power when testing for parameter changes in α^{nm} when α^{nm} changed by +0.2.

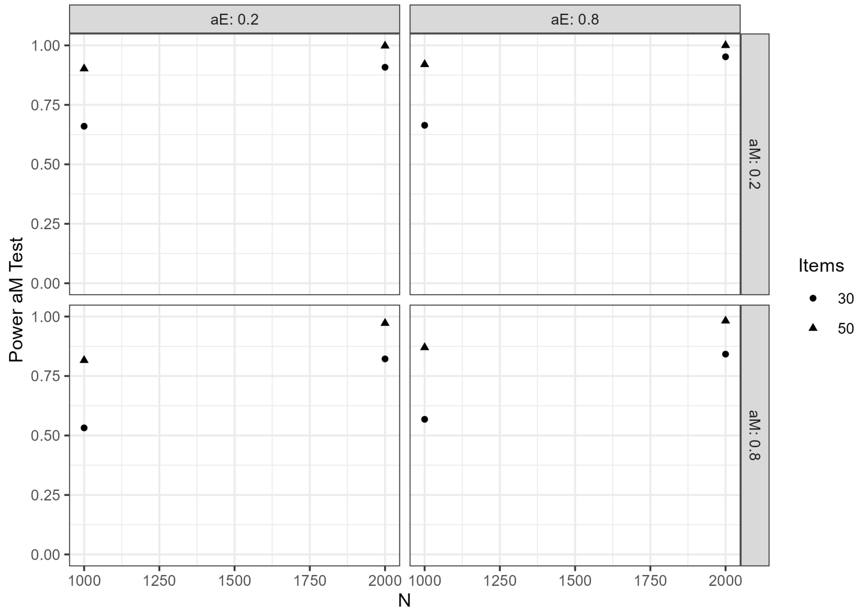

Change of Non-Moderate Response Parameter by -0.5

Figure 61

Type I Error when testing for parameter changes in α^e when α^{nm} changed by -0.5. Red lines indicate the nominal alpha level of 0.05 and an approximate 95% confidence interval for the hit rates.

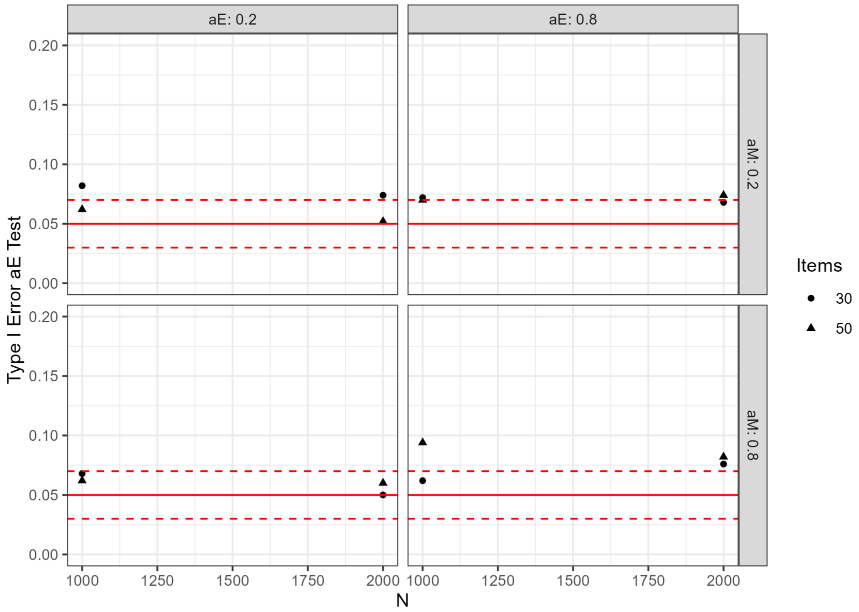

Figure 62

Power when testing for parameter changes in α^{nm} when α^{nm} changed by -0.5.

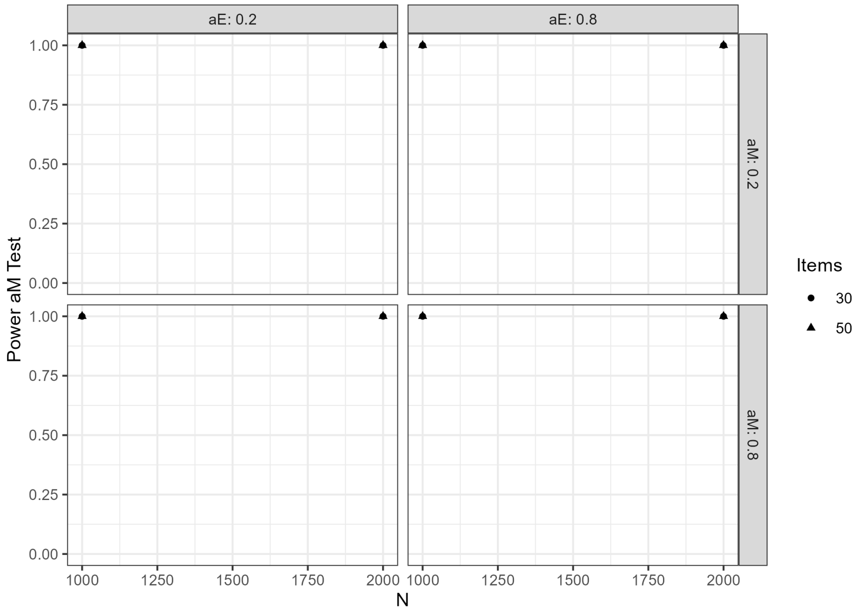

Change of Non-Moderate Response Parameter by -0.2

Figure 63

Type I Error when testing for parameter changes in α^e when α^{nm} changed by -0.2. Red lines indicate the nominal alpha level of 0.05 and an approximate 95% confidence interval for the hit rates.

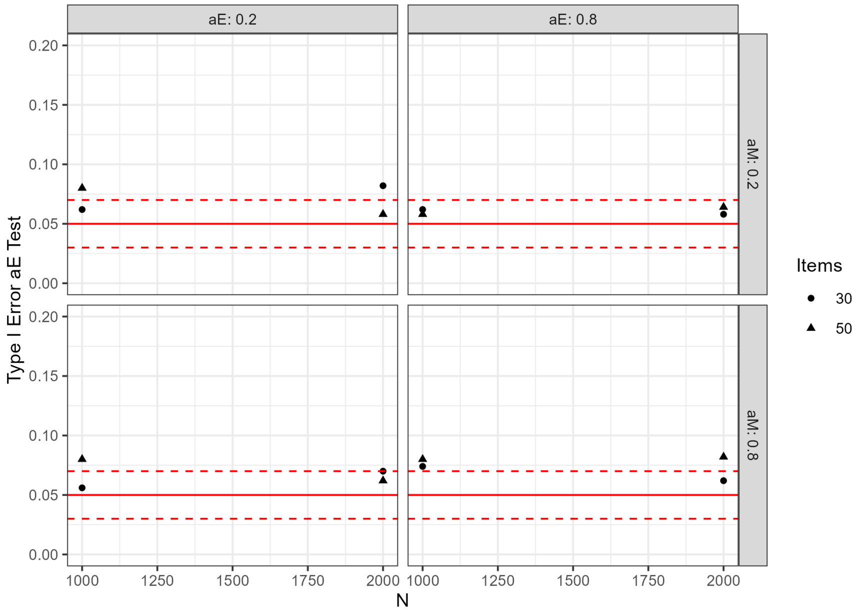

Figure 64

Power when testing for parameter changes in α^{nm} when α^{nm} changed by -0.2.

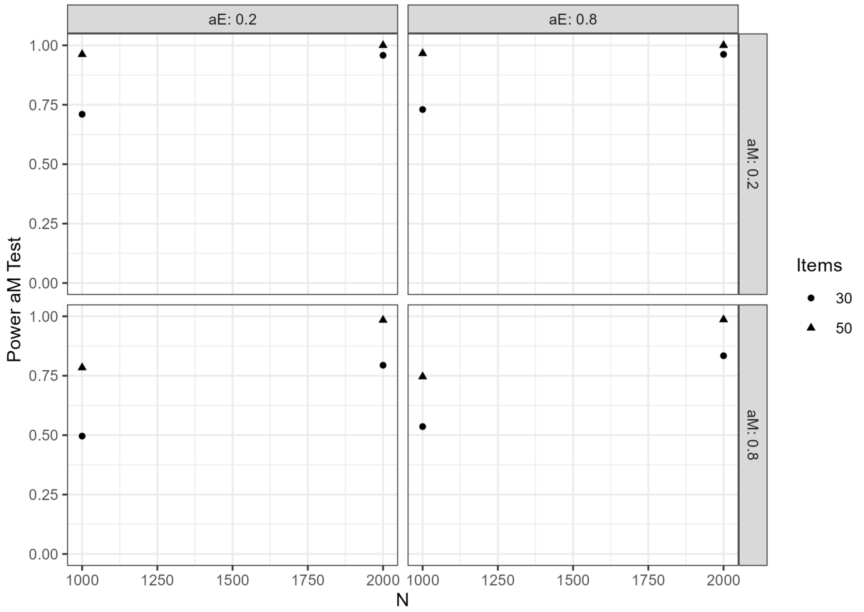

Change of Extreme Response Parameter by +0.5

Figure 65

Power when testing for parameter changes in α^e when α^e changed by +0.5.

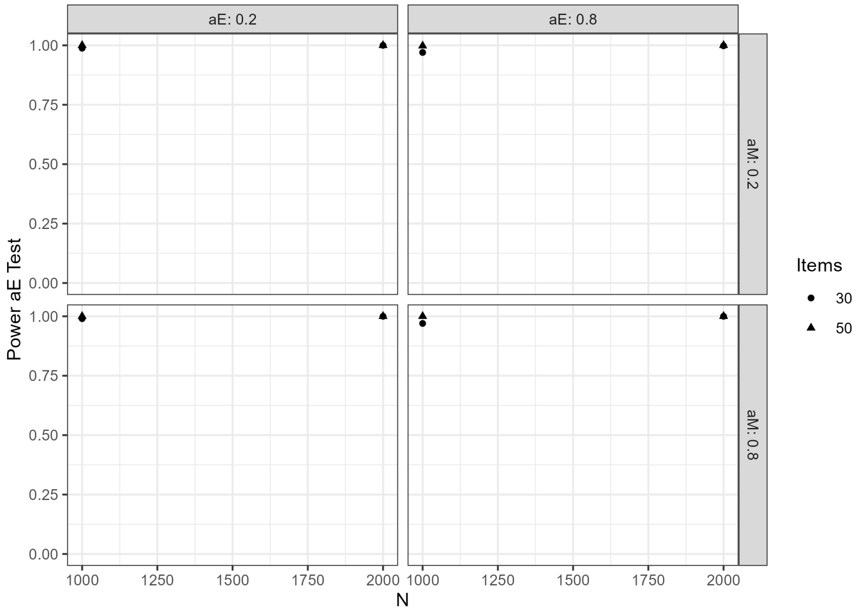

Figure 66

Type I Error when testing for parameter changes in α^{nm} when α^e changed by +0.5. Red lines indicate the nominal alpha level of 0.05 and an approximate 95% confidence interval for the hit rates.

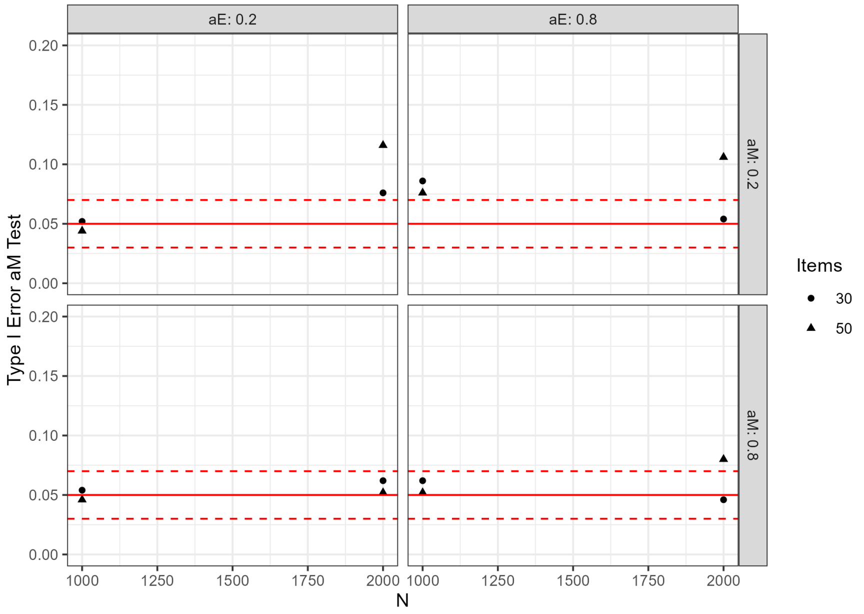

Change of Extreme Response Parameter by +0.2

Figure 67

Power when testing for parameter changes in α^e when α^e changed by +0.2.

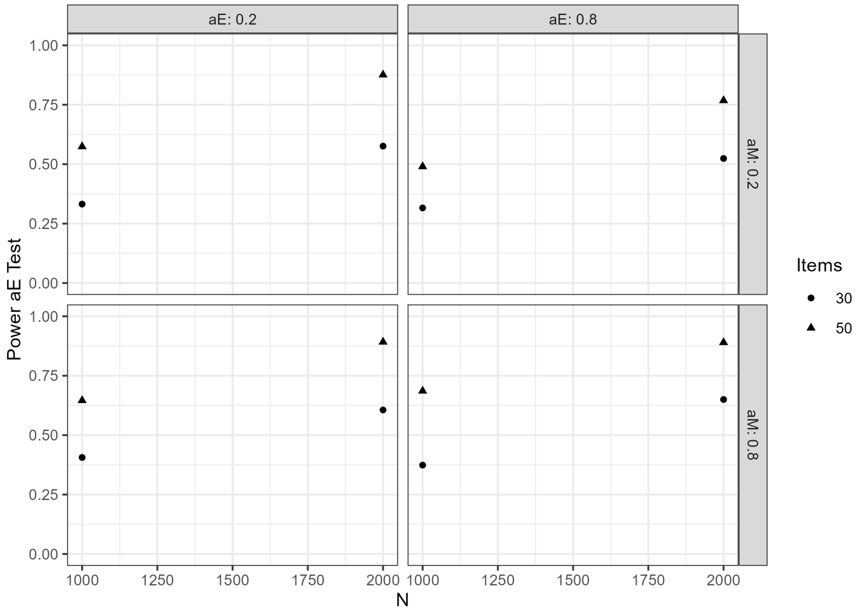

Figure 68

Type I Error when testing for parameter changes in α^{nm} when α^e changed by +0.2. Red lines indicate the nominal alpha level of 0.05 and an approximate 95% confidence interval for the hit rates.

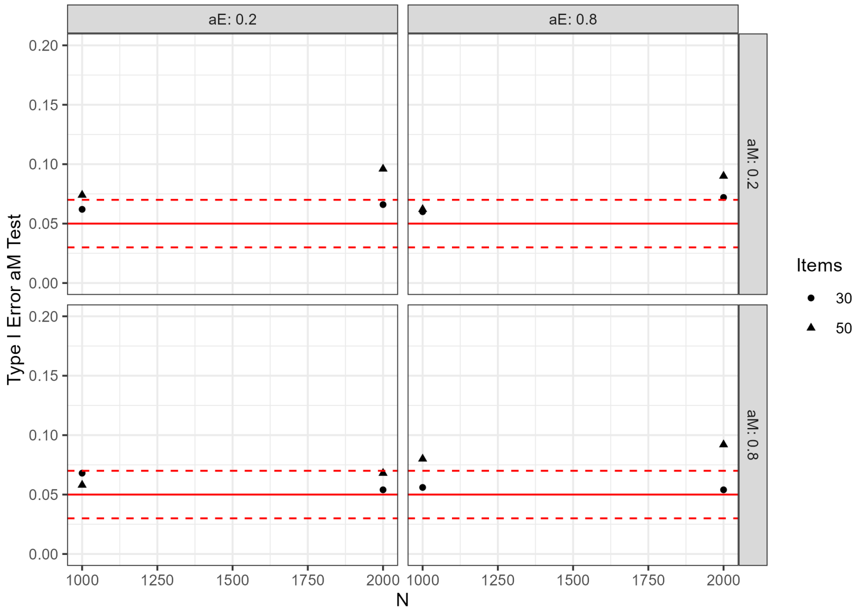

Change of Extreme Response Parameter by -0.5

Figure 69

Power when testing for parameter changes in α^e when α^e changed by -0.5.

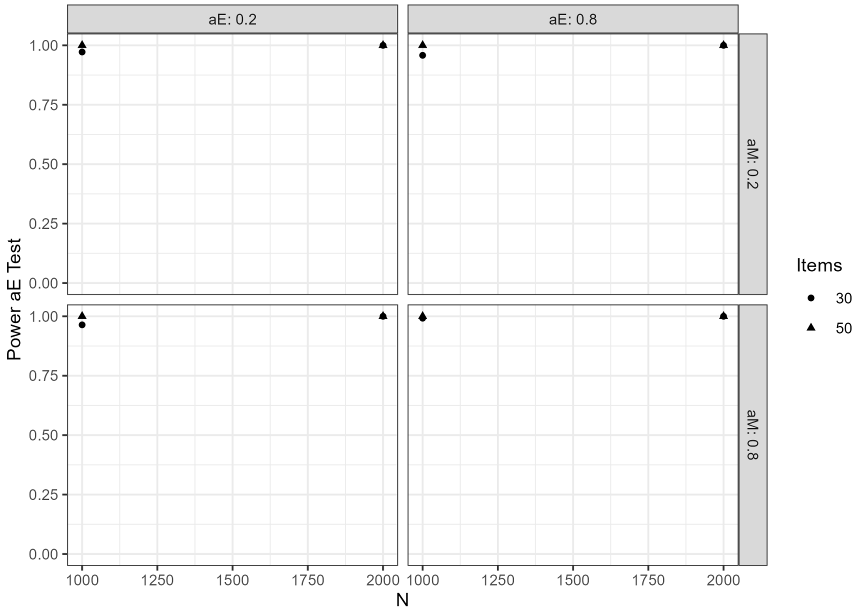

Figure 70

Type I Error when testing for parameter changes in α^{nm} when α^e changed by -0.5. Red lines indicate the nominal alpha level of 0.05 and an approximate 95% confidence interval for the hit rates.

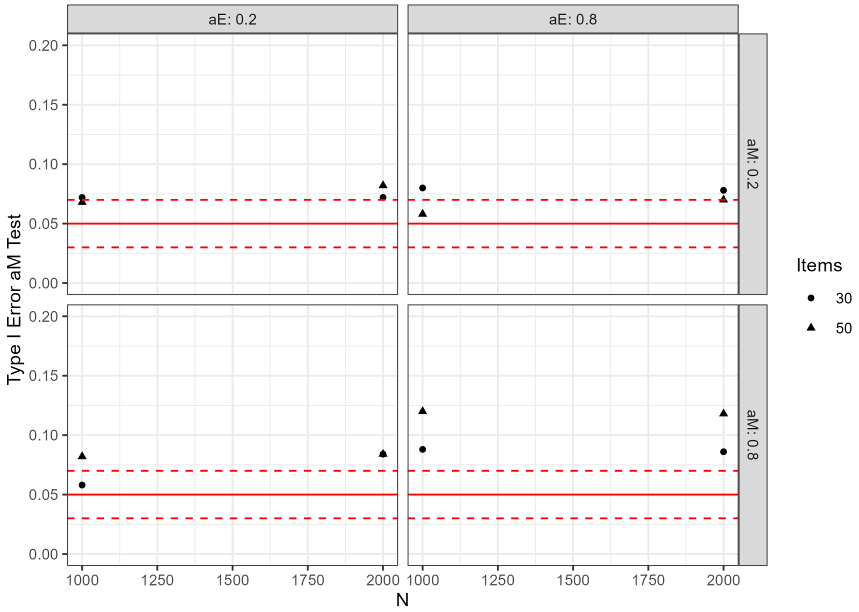

Change of Extreme Response Parameter by -0.2

Figure 71

Power when testing for parameter changes in α^e when α^e changed by -0.2.

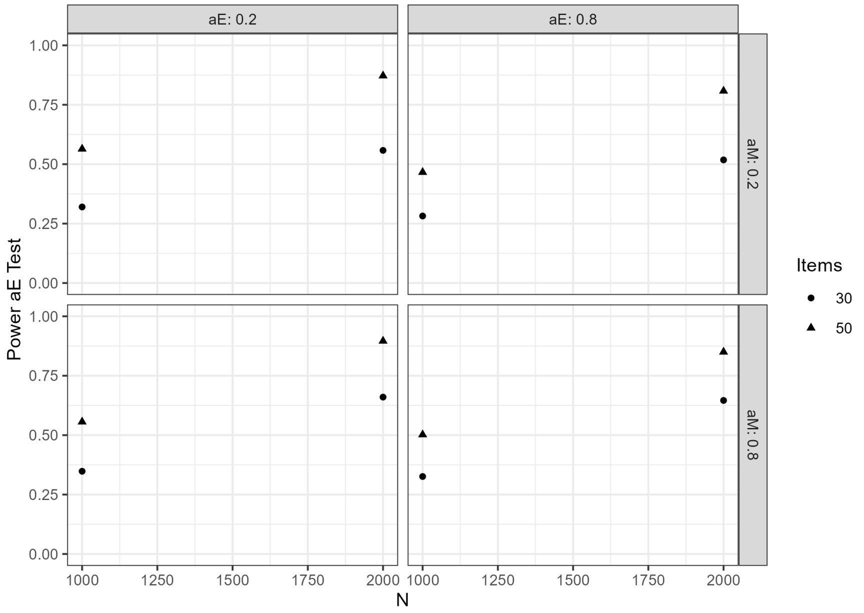

Figure 72

Type I Error when testing for parameter changes in α^{nm} when α^e changed by -0.2. Red lines indicate the nominal alpha level of 0.05 and an approximate 95% confidence interval for the hit rates.

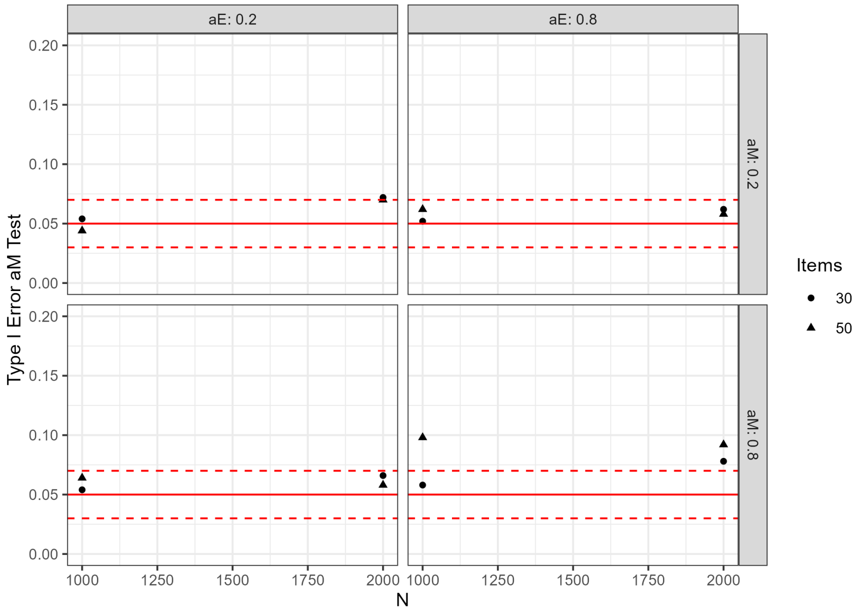

Ordinal Covariate, Test Statistic maxLMo, Skewed Distribution of Covariate

Parameter Invariance

Figure 73

Type I Error when testing for parameter changes in α^e when all parameters are invariant. Red lines indicate the nominal alpha level of 0.05 and an approximate 95% confidence interval for the hit rates.

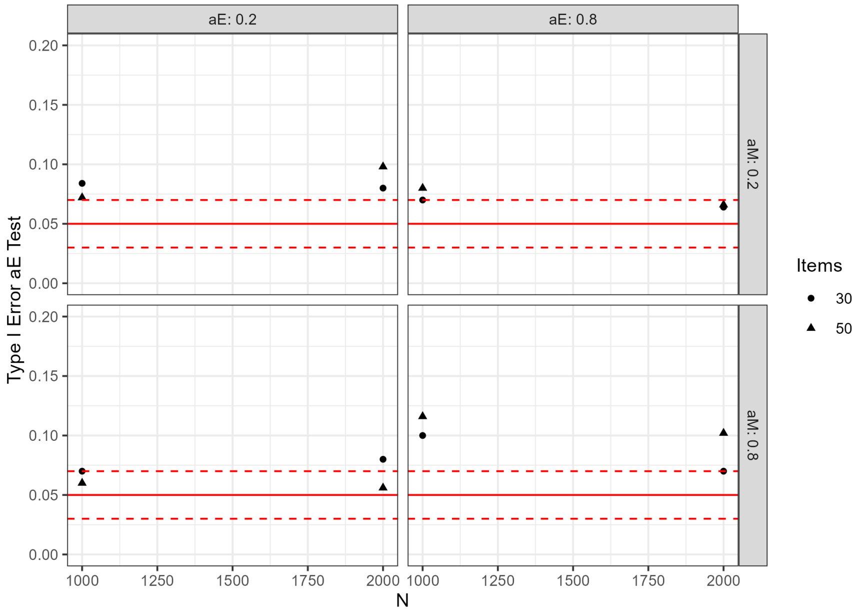

Figure 74

Type I Error when testing for parameter changes in α^{nm} when all parameters are invariant. Red lines indicate the nominal alpha level of 0.05 and an approximate 95% confidence interval for the hit rates.

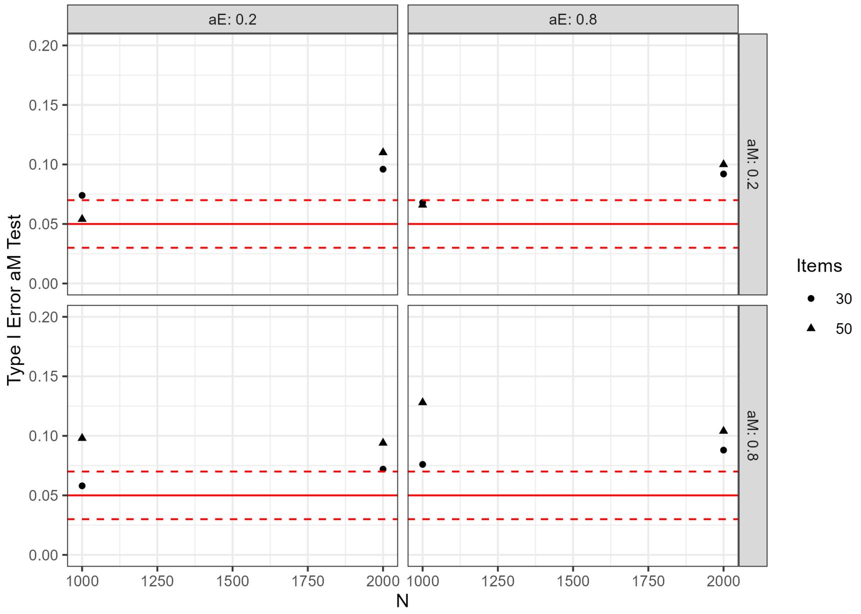

Change of Non-Moderate Response Parameter by +0.5

Figure 75

Type I Error when testing for parameter changes in α^e when α^{nm} changed by +0.5. Red lines indicate the nominal alpha level of 0.05 and an approximate 95% confidence interval for the hit rates.

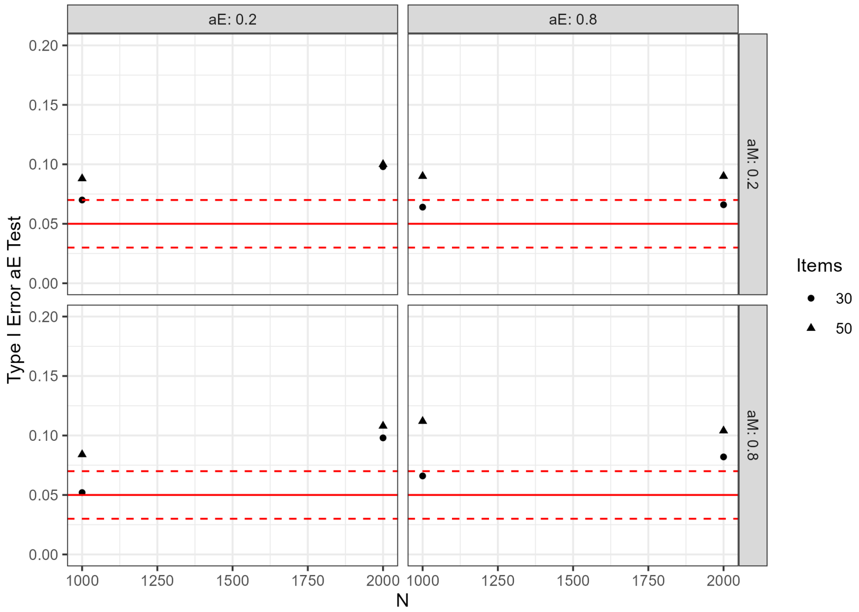

Figure 76

Power when testing for parameter changes in α^{nm} when α^{nm} changed by +0.5.

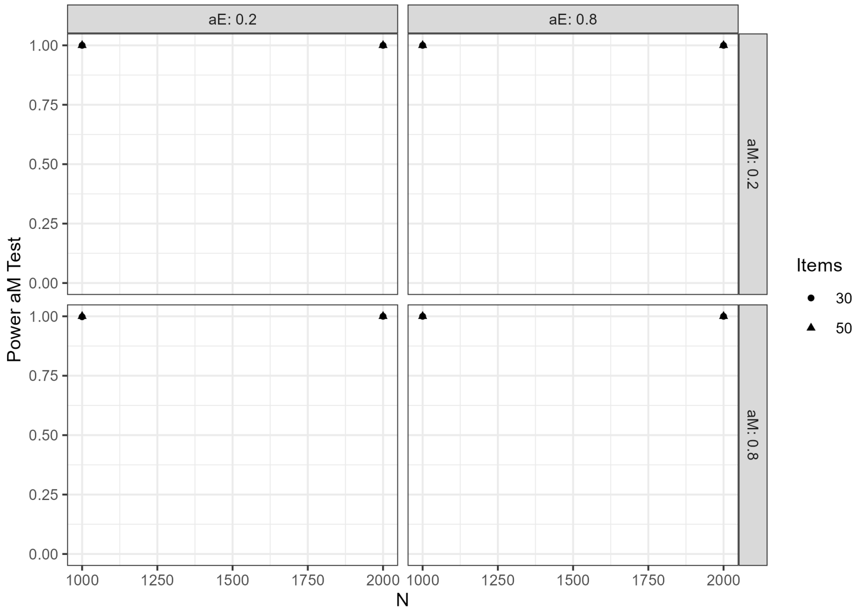

Change of Non-Moderate Response Parameter by +0.2

Figure 77

Type I Error when testing for parameter changes in α^e when α^{nm} changed by +0.2. Red lines indicate the nominal alpha level of 0.05 and an approximate 95% confidence interval for the hit rates.

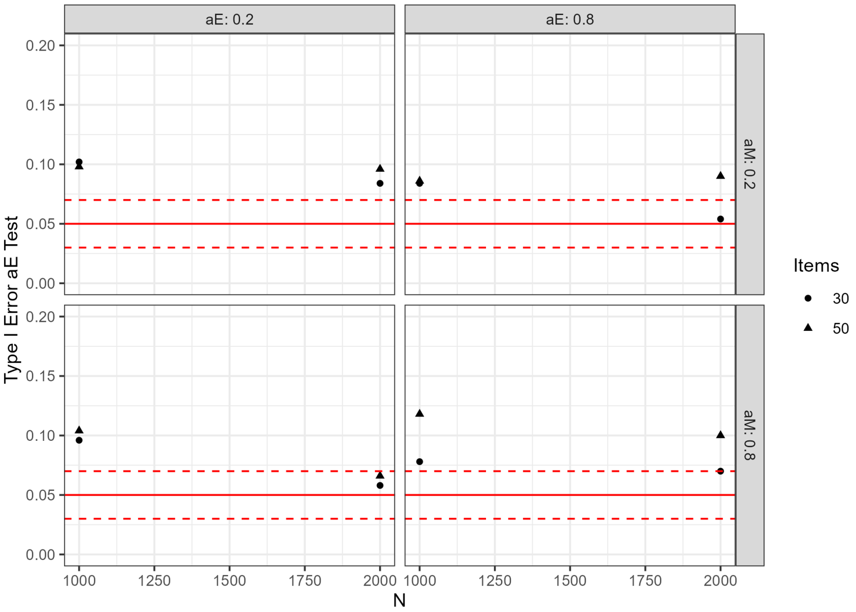

Figure 78

Power when testing for parameter changes in α^{nm} when α^{nm} changed by +0.2.

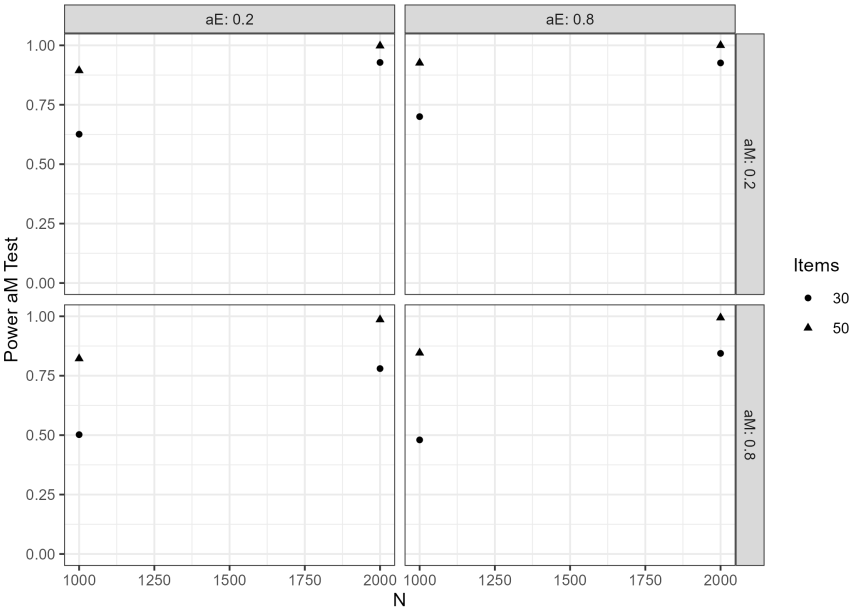

Change of Non-Moderate Response Parameter by -0.5

Figure 79

Type I Error when testing for parameter changes in α^e when α^{nm} changed by -0.5. Red lines indicate the nominal alpha level of 0.05 and an approximate 95% confidence interval for the hit rates.

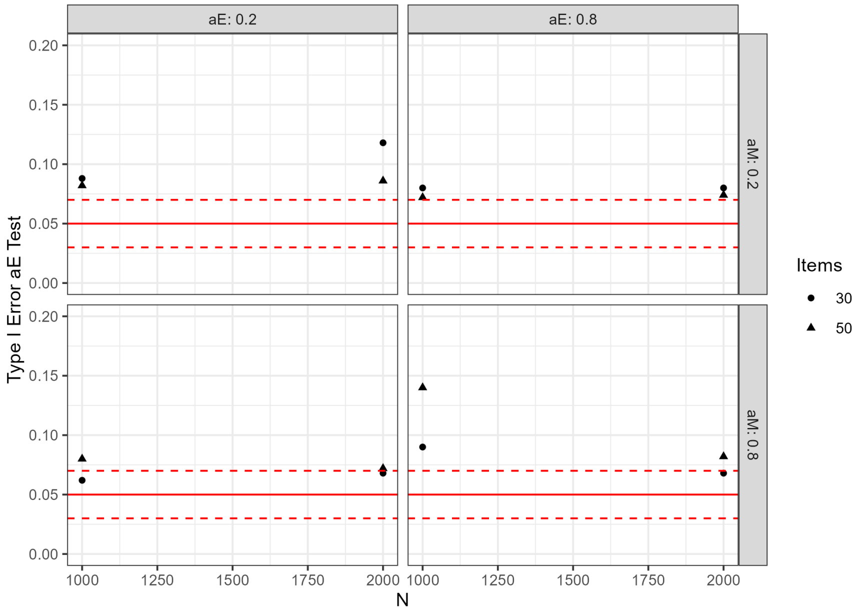

Figure 80

Power when testing for parameter changes in α^{nm} when α^{nm} changed by -0.5.

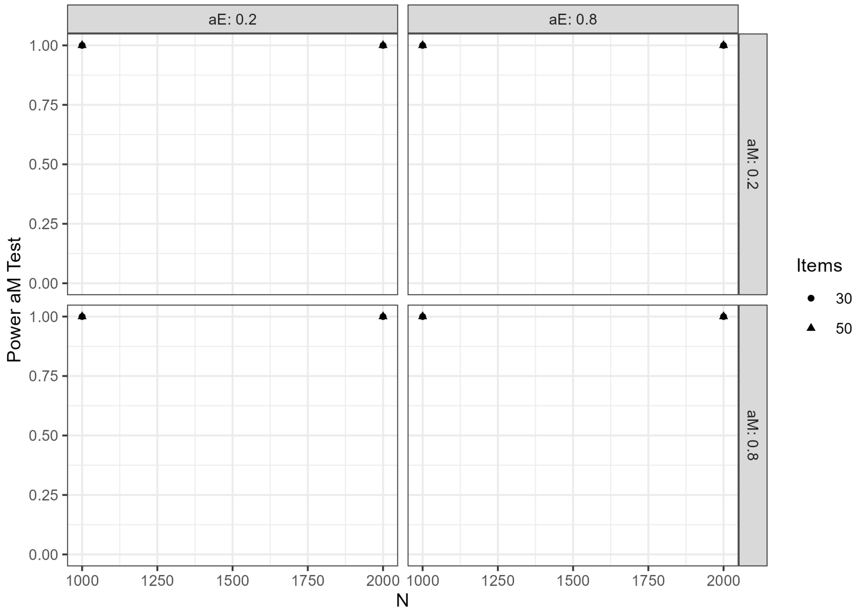

Change of Non-Moderate Response Parameter by -0.2

Figure 81

Type I Error when testing for parameter changes in α^e when α^{nm} changed by -0.2. Red lines indicate the nominal alpha level of 0.05 and an approximate 95% confidence interval for the hit rates.

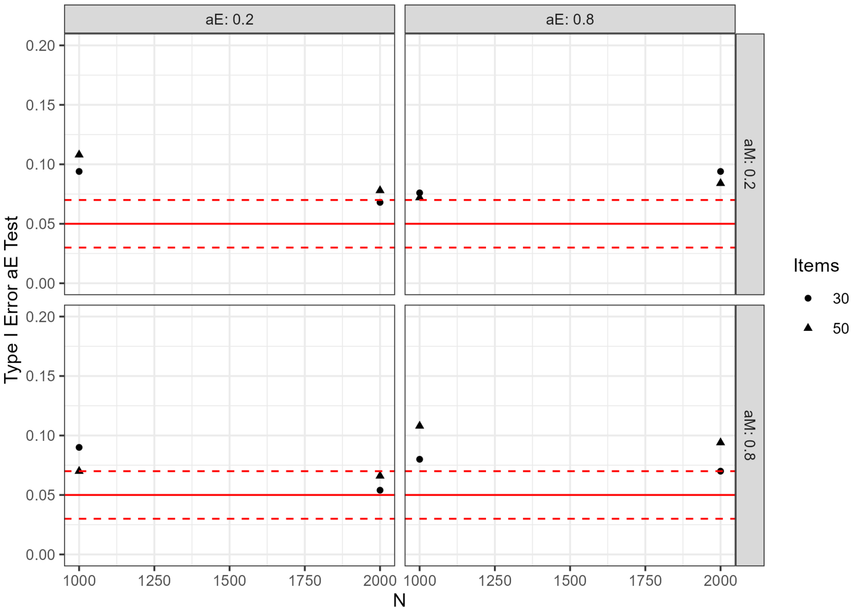

Figure 82

Power when testing for parameter changes in α^{nm} when α^{nm} changed by -0.2.

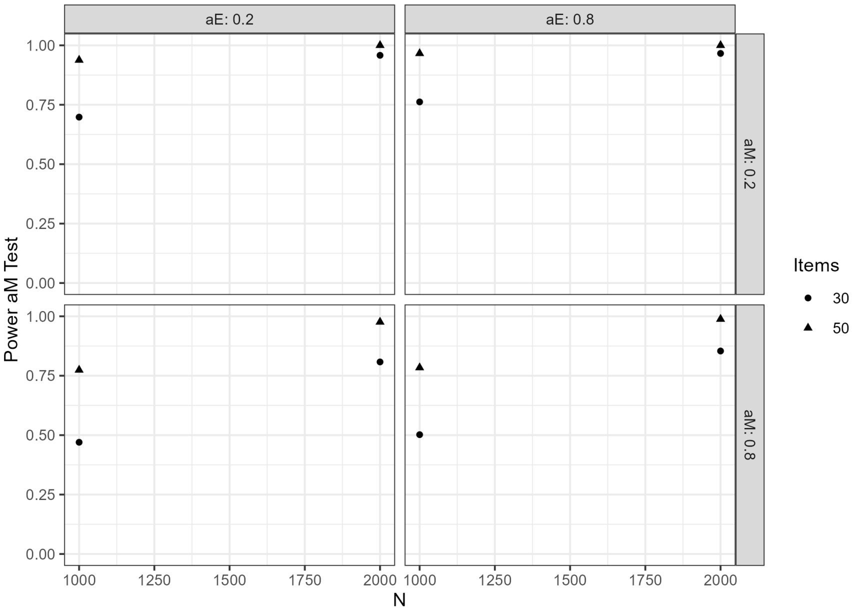

Change of Extreme Response Parameter by +0.5

Figure 83

Power when testing for parameter changes in α^e when α^e changed by +0.5.

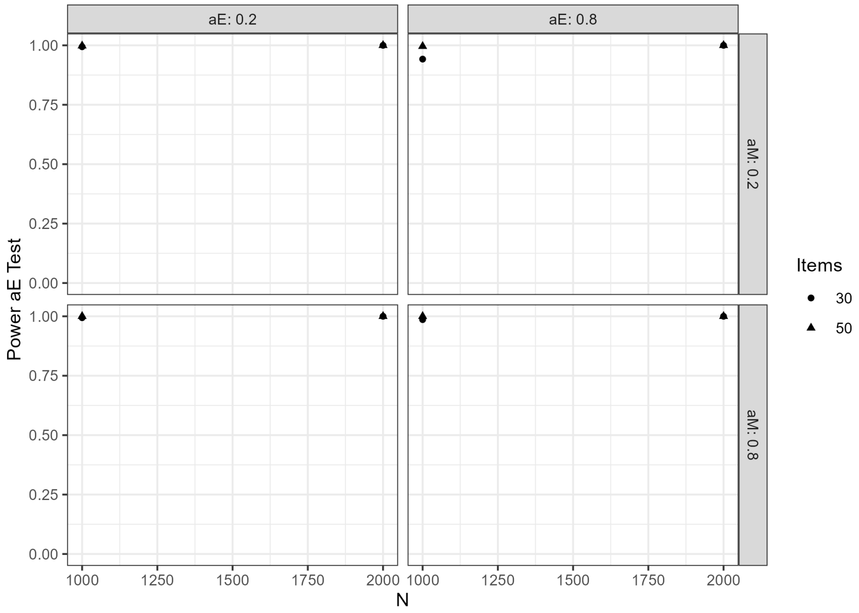

Figure 84

Type I Error when testing for parameter changes in α^{nm} when α^e changed by +0.5. Red lines indicate the nominal alpha level of 0.05 and an approximate 95% confidence interval for the hit rates.

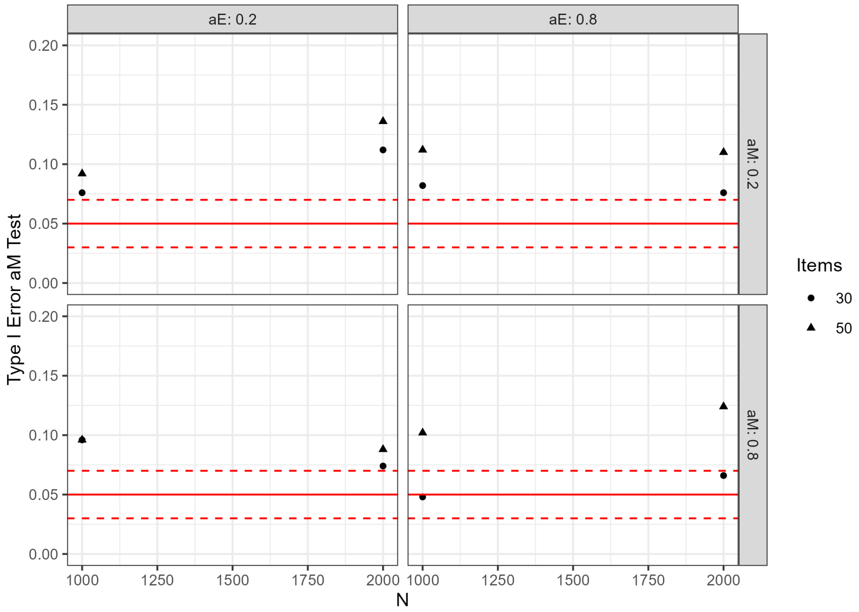

Change of Extreme Response Parameter by +0.2

Figure 85

Power when testing for parameter changes in α^e when α^e changed by +0.2.

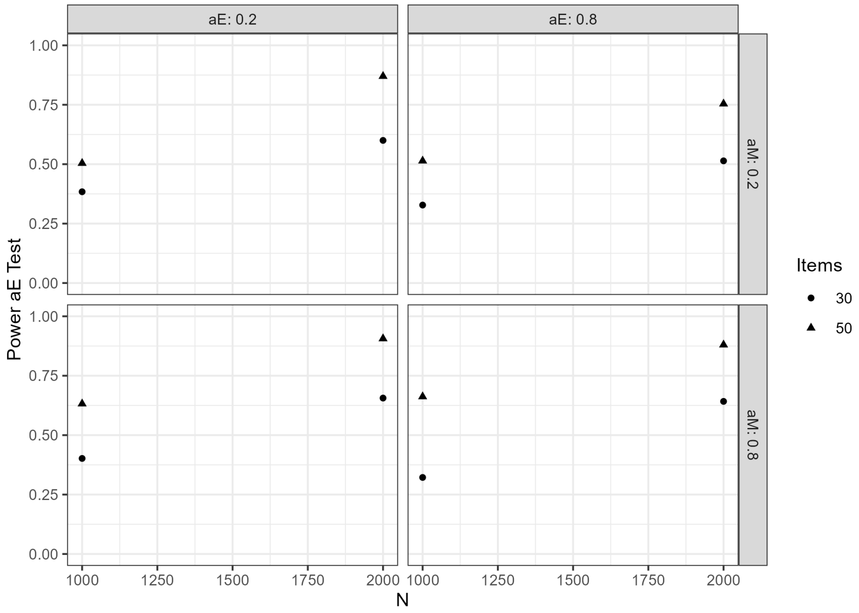

Figure 86

Type I Error when testing for parameter changes in α^{nm} when α^e changed by +0.2. Red lines indicate the nominal alpha level of 0.05 and an approximate 95% confidence interval for the hit rates.

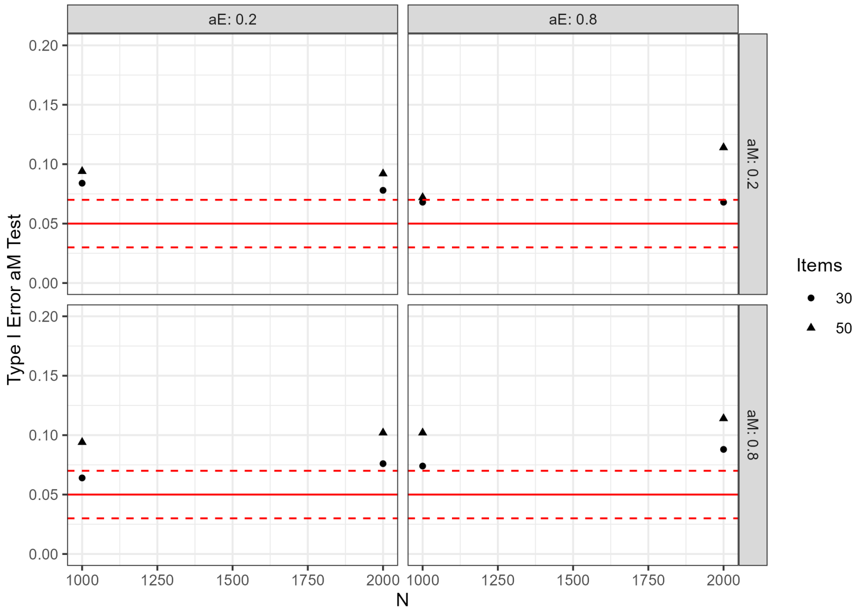

Change of Extreme Response Parameter by -0.5

Figure 87

Power when testing for parameter changes in α^e when α^e changed by -0.5.

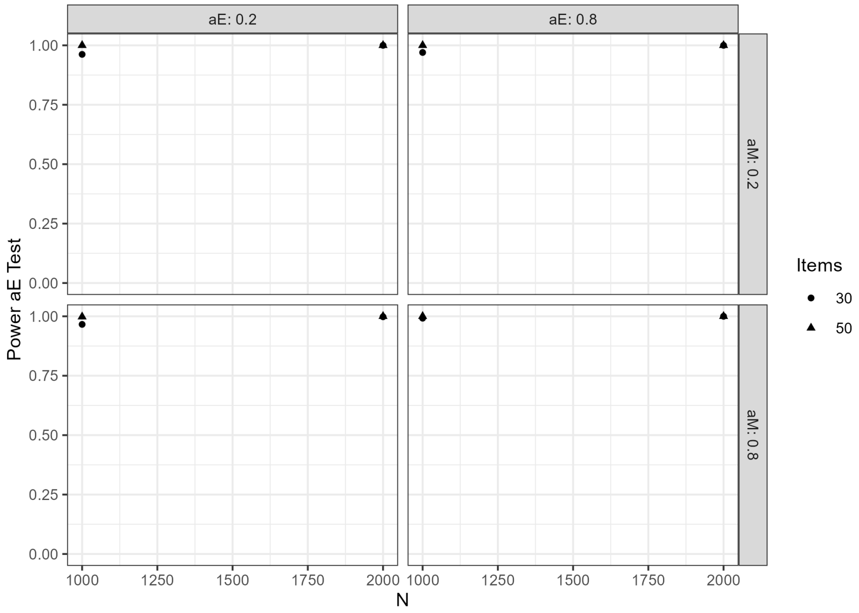

Figure 88

Type I Error when testing for parameter changes in α^{nm} when α^e changed by -0.5. Red lines indicate the nominal alpha level of 0.05 and an approximate 95% confidence interval for the hit rates.

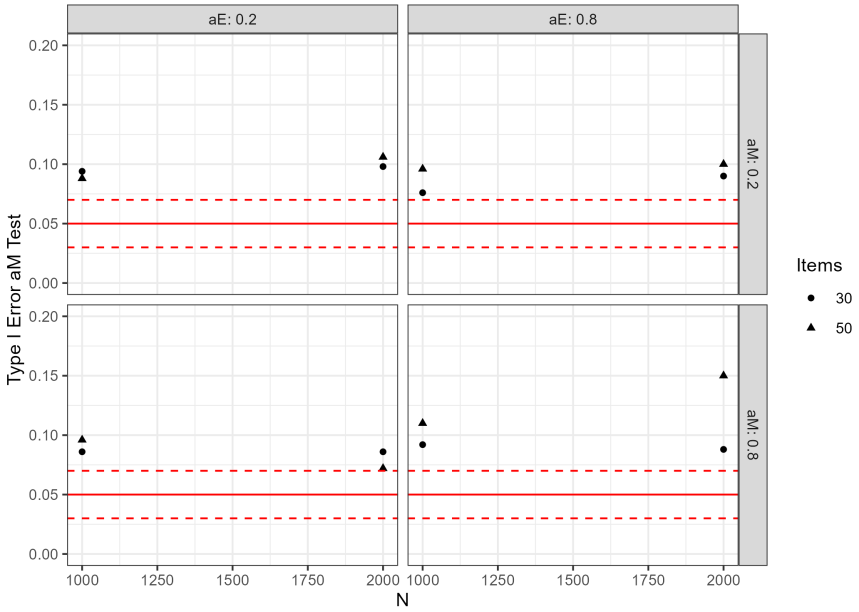

Change of Extreme Response Parameter by -0.2

Figure 89

Power when testing for parameter changes in α^e when α^e changed by -0.2.

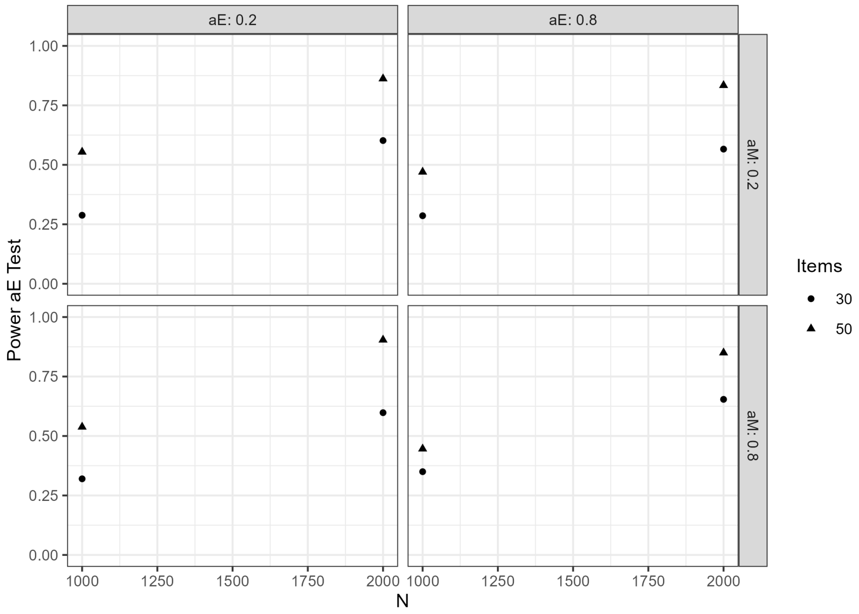

Figure 90

Type I Error when testing for parameter changes in α^{nm} when α^e changed by -0.2. Red lines indicate the nominal alpha level of 0.05 and an approximate 95% confidence interval for the hit rates.

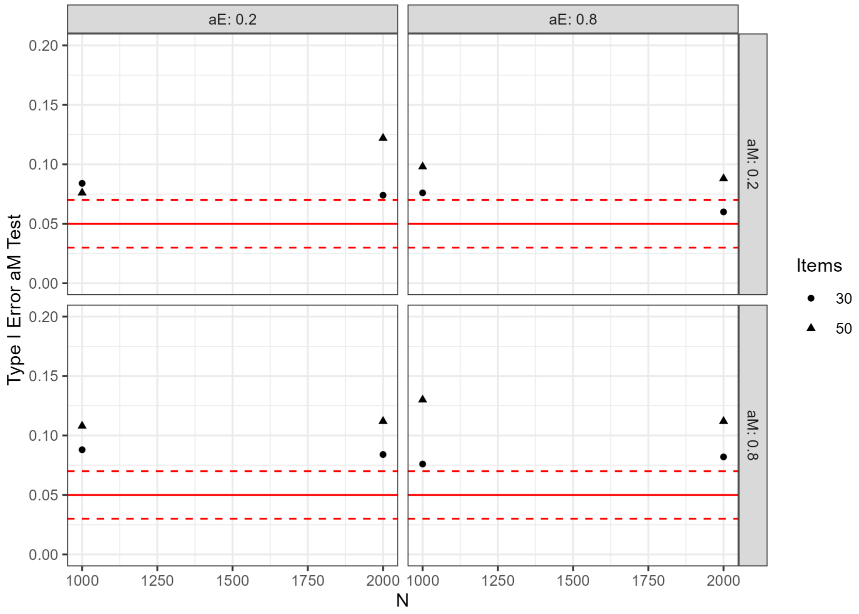

Categorical Covariate, Test Statistic LMuo

Parameter Invariance

Figure 91

Type I Error when testing for parameter changes in α^e when all parameters are invariant. Red lines indicate the nominal alpha level of 0.05 and an approximate 95% confidence interval for the hit rates.

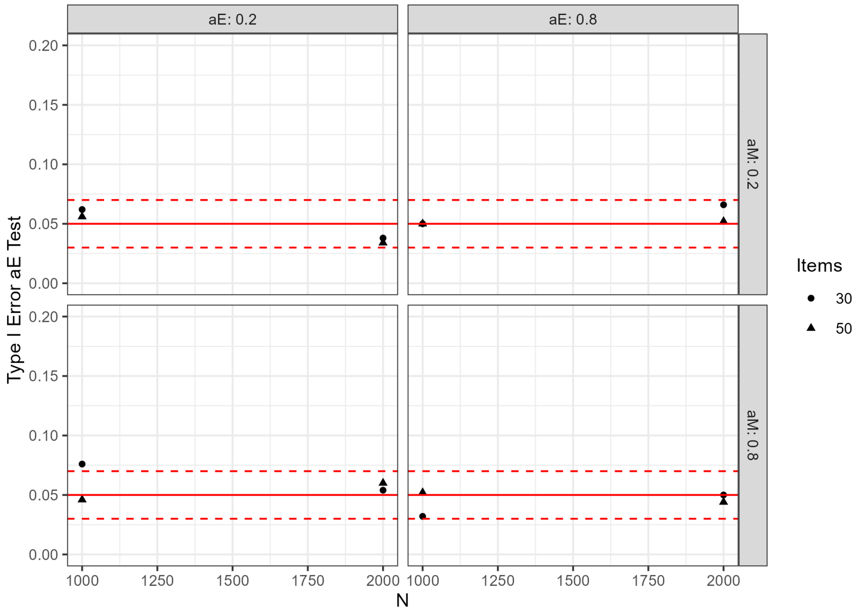

Figure 92

Type I Error when testing for parameter changes in α^{nm} when all parameters are invariant. Red lines indicate the nominal alpha level of 0.05 and an approximate 95% confidence interval for the hit rates.

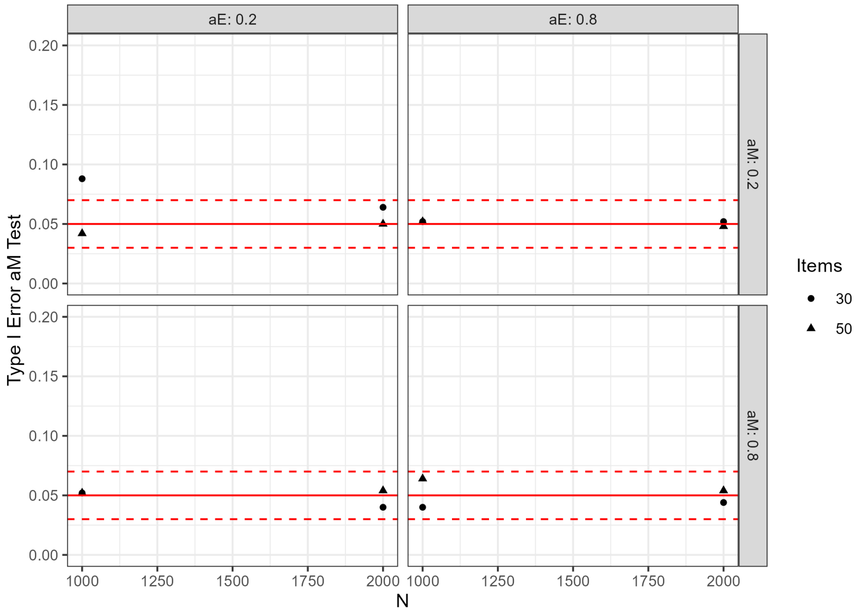

Change of Non-Moderate Response Parameter by +0.5

Figure 93

Type I Error when testing for parameter changes in α^e when α^{nm} changed by +0.5.

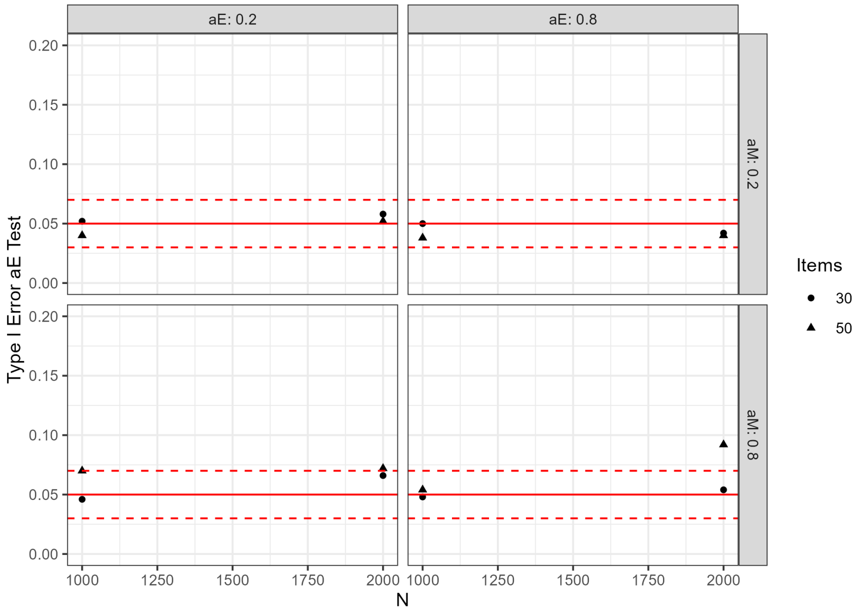

Figure 94

Power when testing for parameter changes in α^{nm} when α^{nm} changed by +0.5.

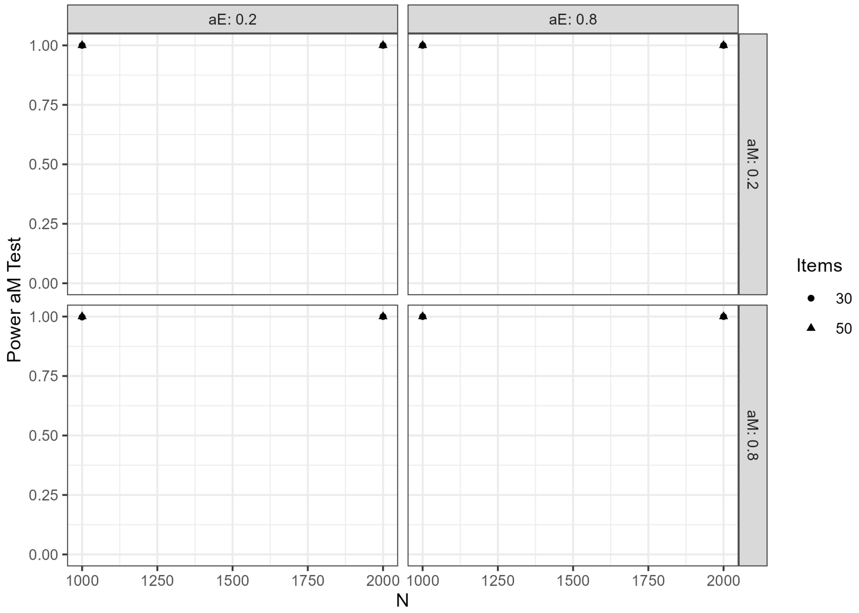

Change of Non-Moderate Response Parameter by +0.2

Figure 95

Type I Error when testing for parameter changes in α^e when α^{nm} changed by +0.2.

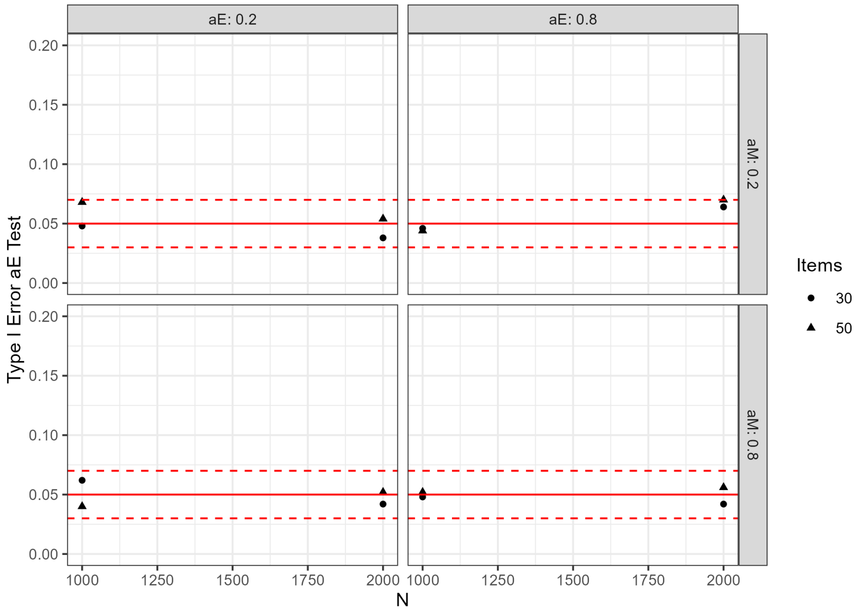

Figure 96

Power when testing for parameter changes in α^{nm} when α^{nm} changed by +0.2.

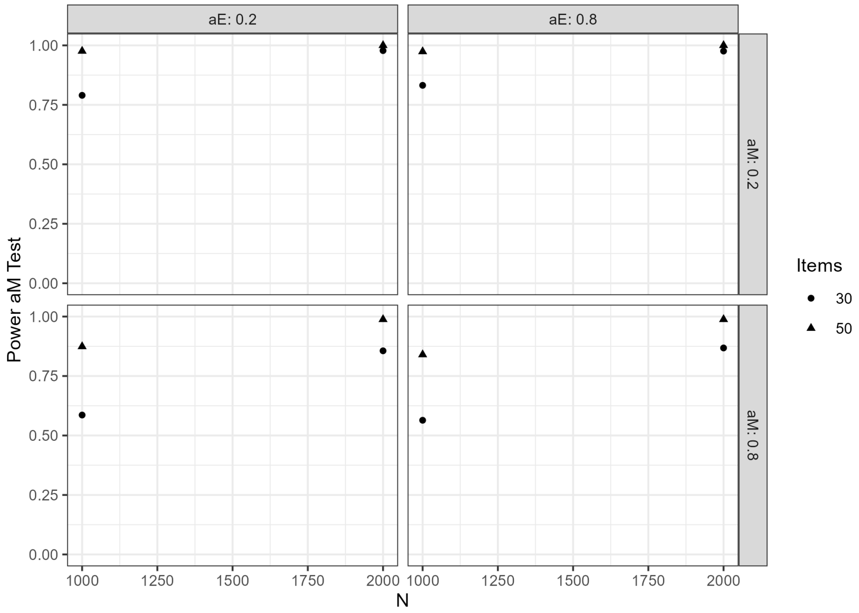

Change of Non-Moderate Response Parameter by -0.5

Figure 97

Type I Error when testing for parameter changes in α^e when α^{nm} changed by -0.5.

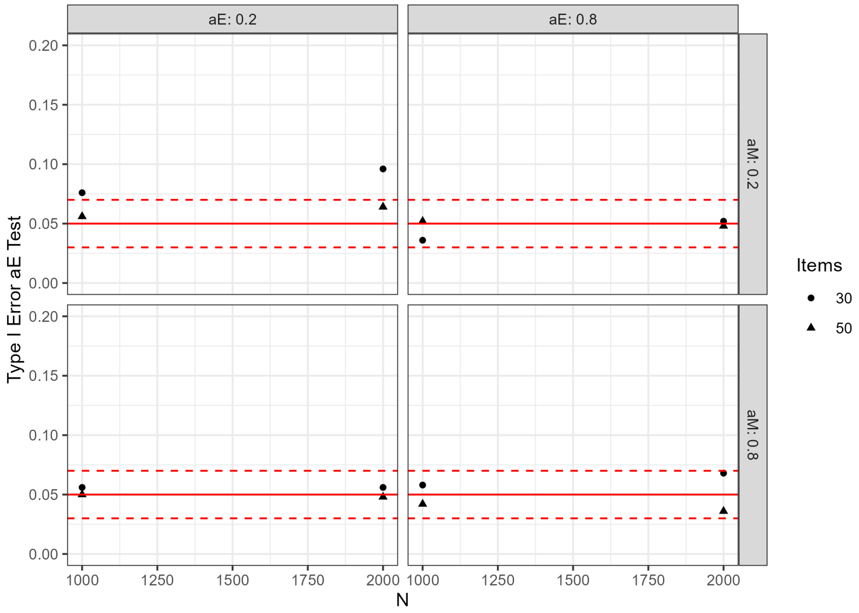

Figure 98

Power when testing for parameter changes in α^{nm} when α^{nm} changed by -0.5.

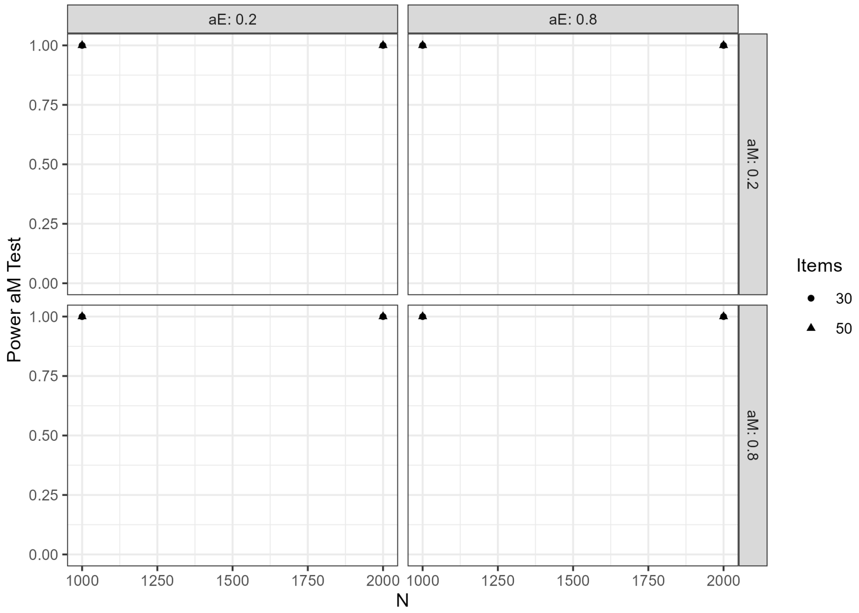

Change of Non-Moderate Response Parameter by -0.2

Figure 99

Type I Error when testing for parameter changes in α^e when α^{nm} changed by -0.2.

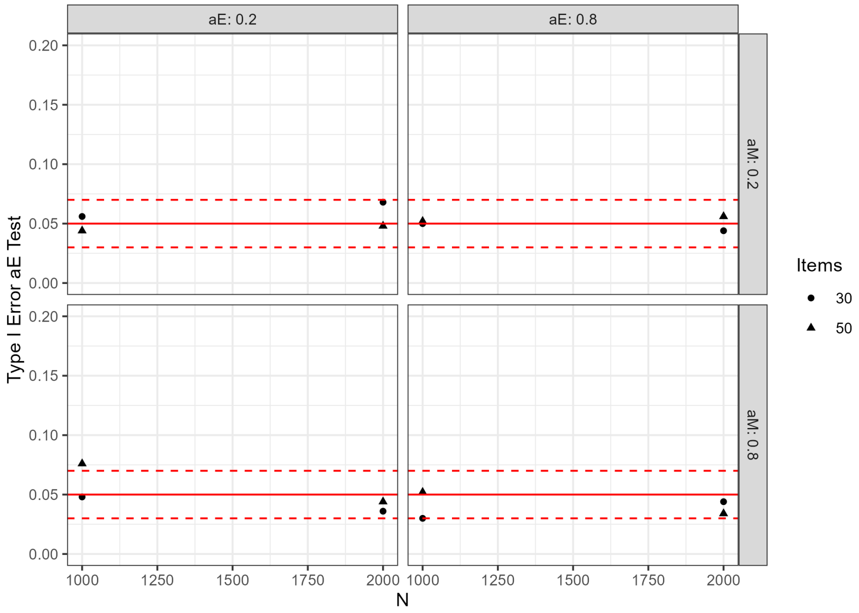

Figure 100

Power when testing for parameter changes in α^{nm} when α^{nm} changed by -0.2.

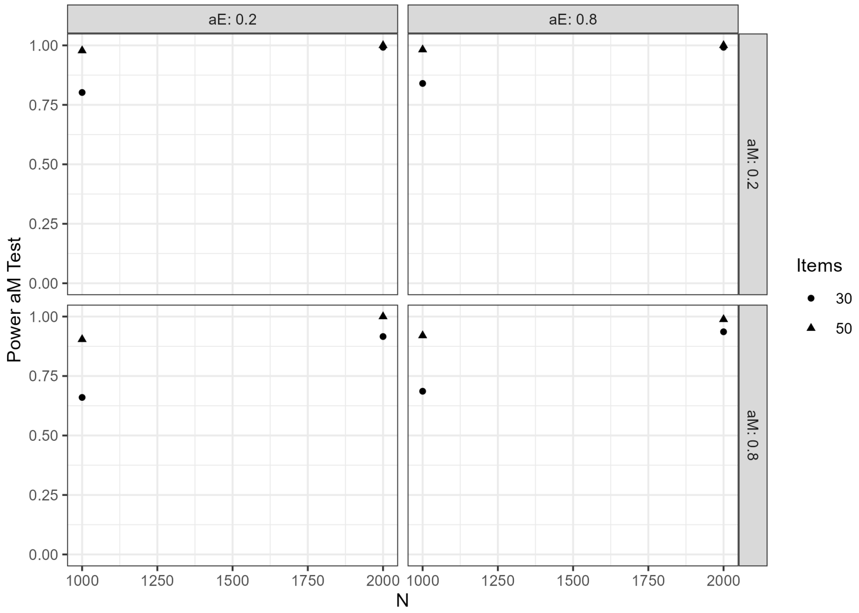

Change of Extreme Response Parameter by +0.5

Figure 101

Power when testing for parameter changes in α^e when α^e changed by +0.5.

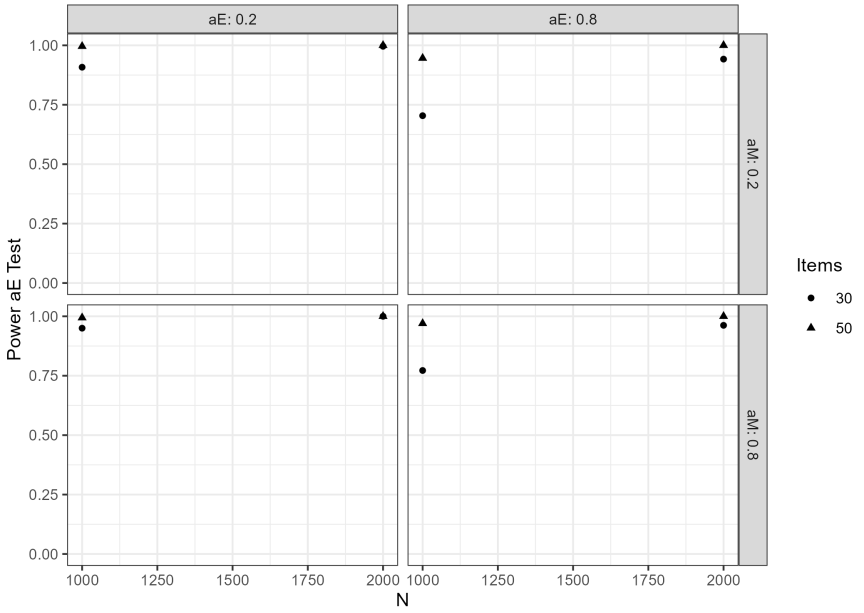

Figure 102

Type I Error when testing for parameter changes in α^{nm} when α^e changed by +0.5.

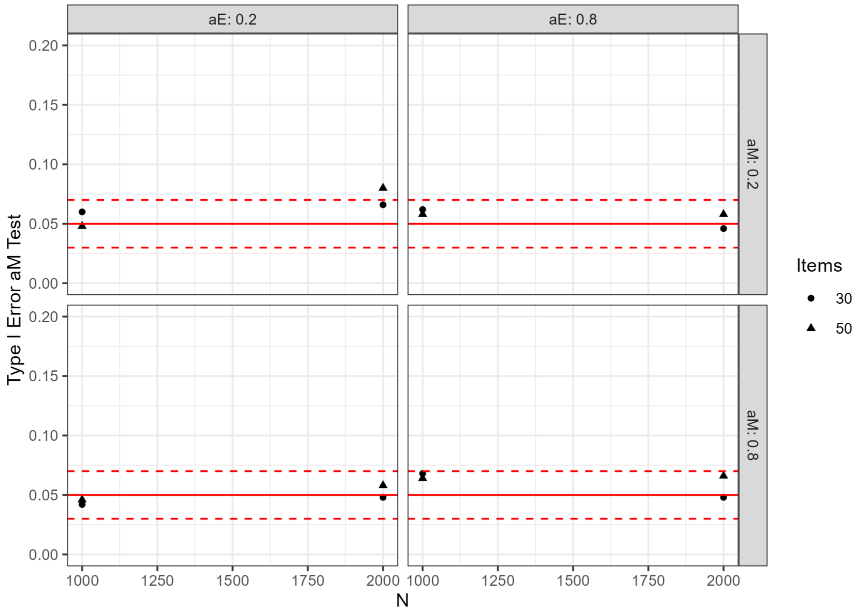

Change of Extreme Response Parameter by +0.2

Figure 103

Power when testing for parameter changes in α^e when α^e changed by +0.2.

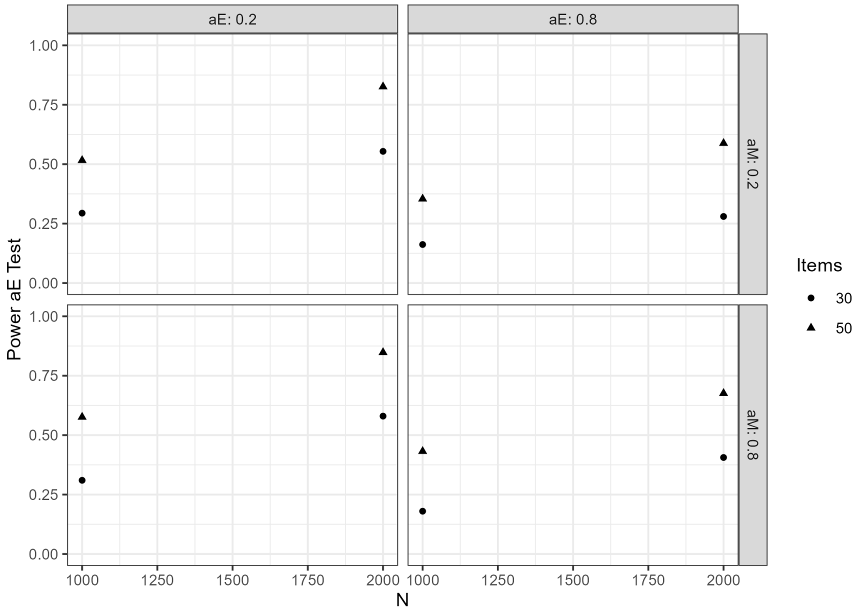

Figure 104

Type I Error when testing for parameter changes in α^{nm} when α^e changed by +0.2.

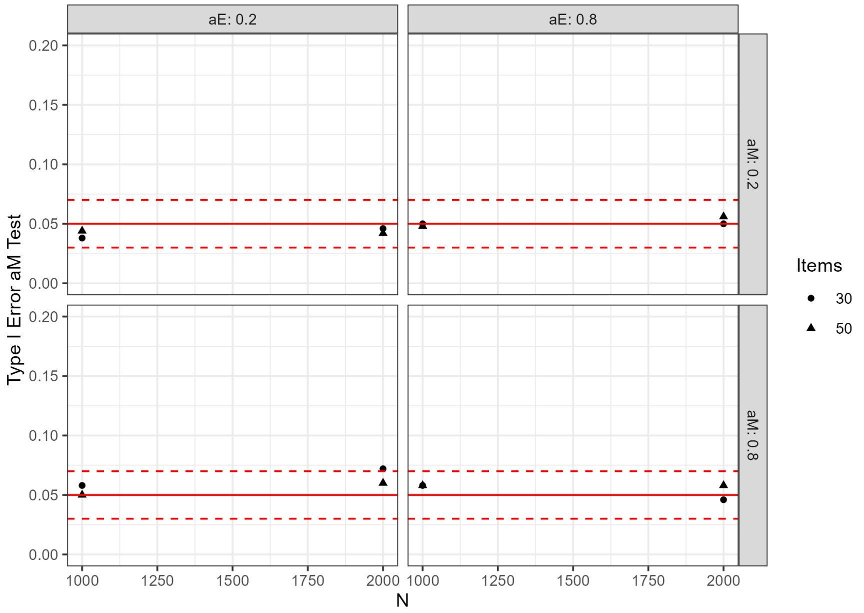

Change of Extreme Response Parameter by -0.5

Figure 105

Power when testing for parameter changes in α^e when α^e changed by -0.5.

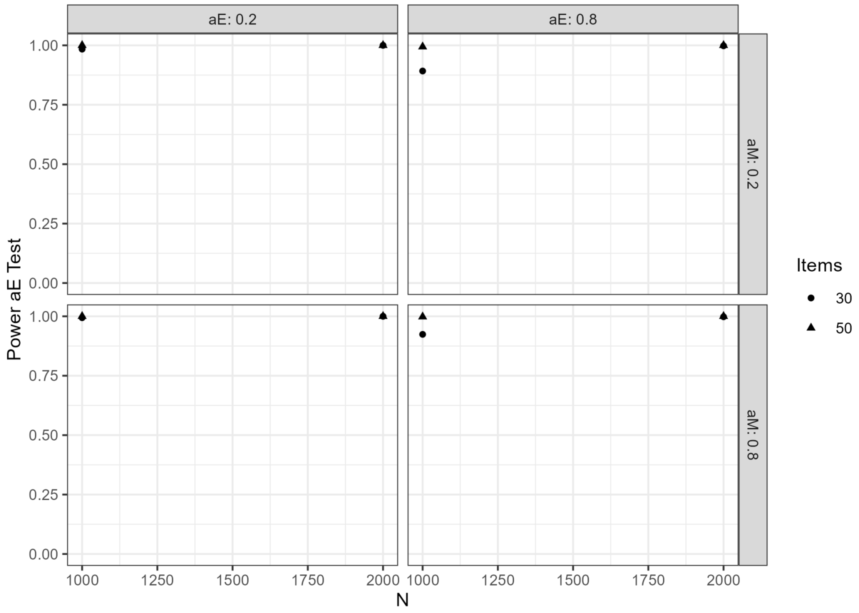

Figure 106

Type I Error when testing for parameter changes in α^{nm} when α^e changed by -0.5.

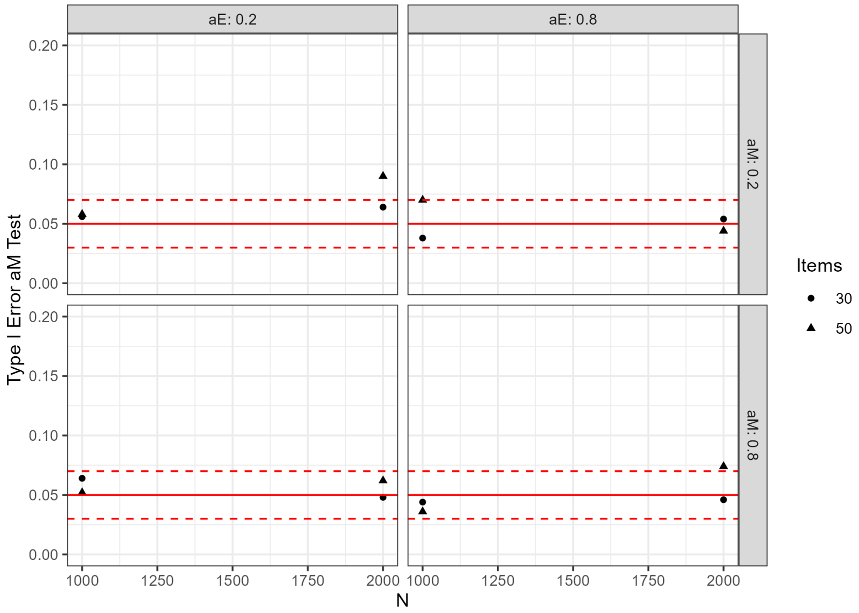

Change of Extreme Response Parameter by -0.2

Figure 107

Power when testing for parameter changes in α^e when α^e changed by -0.2.

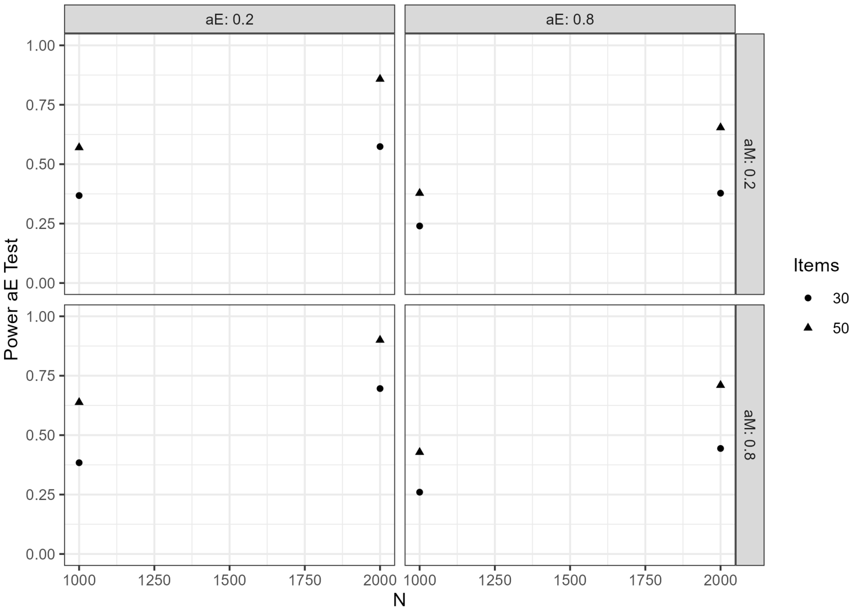

Figure 108

Type I Error when testing for parameter changes in α^{nm} when α^e changed by -0.2.

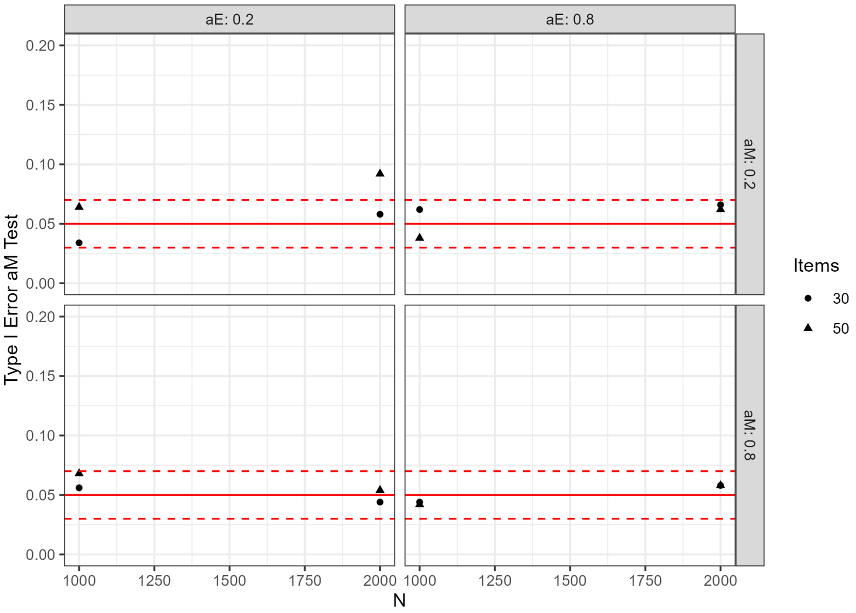

Supplement: Supplementary file 1 — Data S1. [file BMSP-78-420-s001.pdf]
