# Supplementary material for: Investigating heterogeneity in IRTree models for multiple response processes with score‐based partitioning
Source: Br J Math Stat Psychol. 2024 Nov 4;78(2):420–39. doi: 10.1111/bmsp.12367 (PMC11971602; doi:10.1111/bmsp.12367)

Supplementary Material: Investigating Heterogeneity in IRTree Models for Response Styles with Score-Based Partitioning

This supplementary document provides the complete results on the split point detection in conditions with parameter changes. We sort the results by type of covariate and test statistic as well as the specific condition. In conditions with a numerical covariate, the true split point is 40, wheres in conditions with an ordinal covariate, it is 3.

Numerical Covariate, Test Statistic DM

Change of Non-Moderate Response Parameter by +0.5

Figure 1

The mean proposed cutpoint under different conditions of sample size, test length and factor loadings when α^{nm} was affected by a parameter change of +0.5. The red line denotes the true cutpoint of 40.

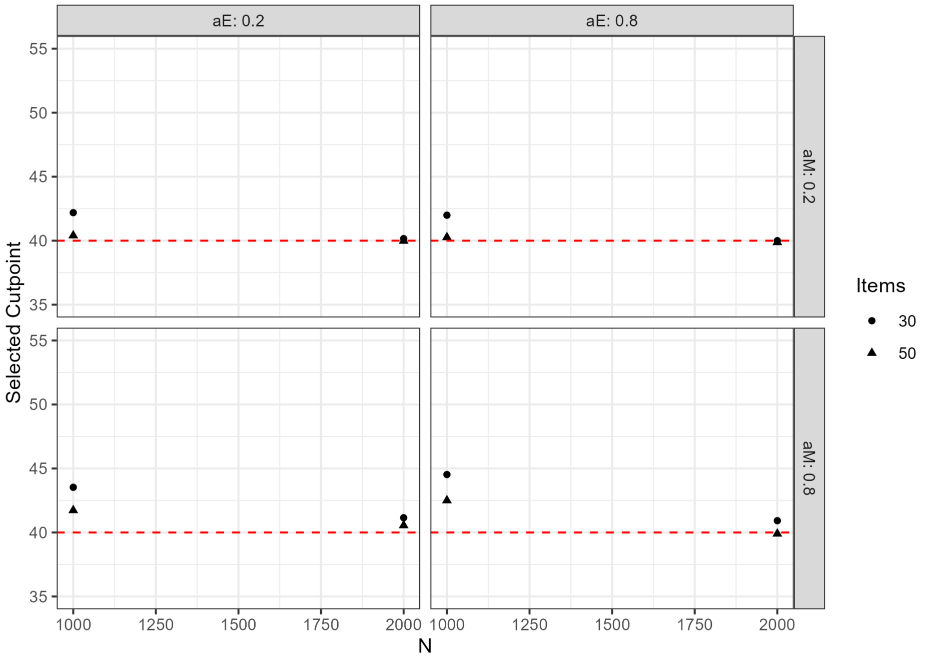

Change of Non-Moderate Response Parameter by +0.2

Figure 2

The mean proposed cutpoint under different conditions of sample size, test length and factor loadings when α^{nm} was affected by a parameter change of +0.2. The red line denotes the true cutpoint of 40.

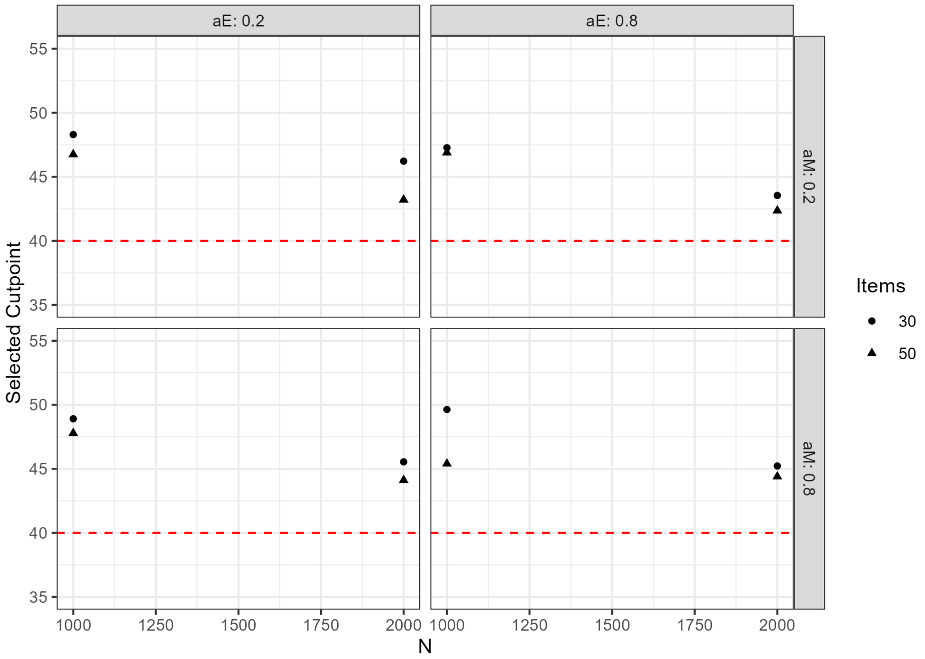

Change of Non-Moderate Response Parameter by -0.5

Figure 3

The mean proposed cutpoint under different conditions of sample size, test length and factor loadings when α^{nm} *was affected by a parameter change of -0.5. The red line denotes the true cutpoint of 40.*

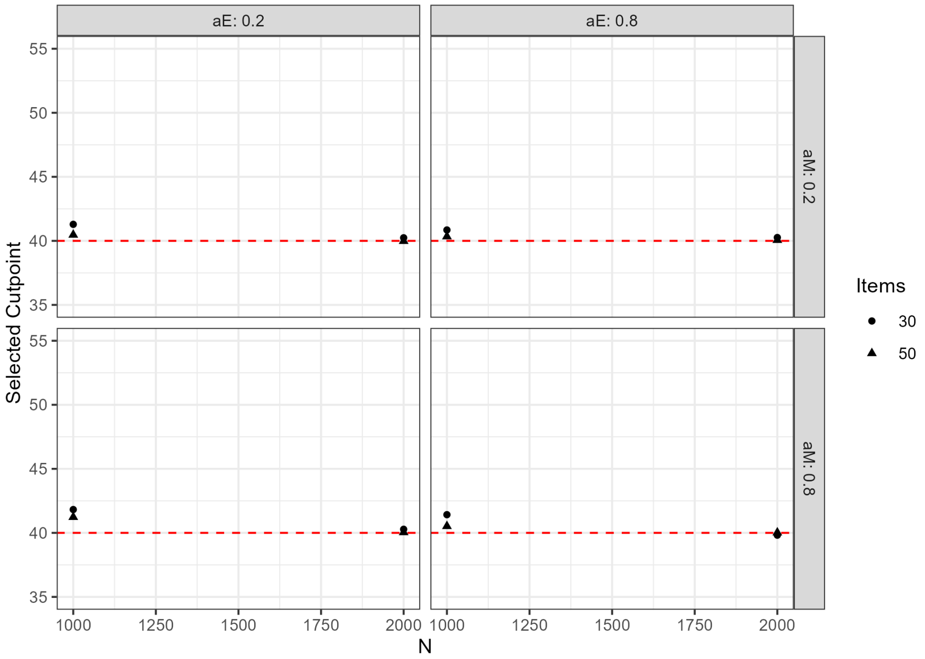

Change of Non-Moderate Response Parameter by -0.2

Figure 4

The mean proposed cutpoint under different conditions of sample size, test length and factor loadings when α^{nm} *was affected by a parameter change of -0.2. The red line denotes the true cutpoint of 40.*

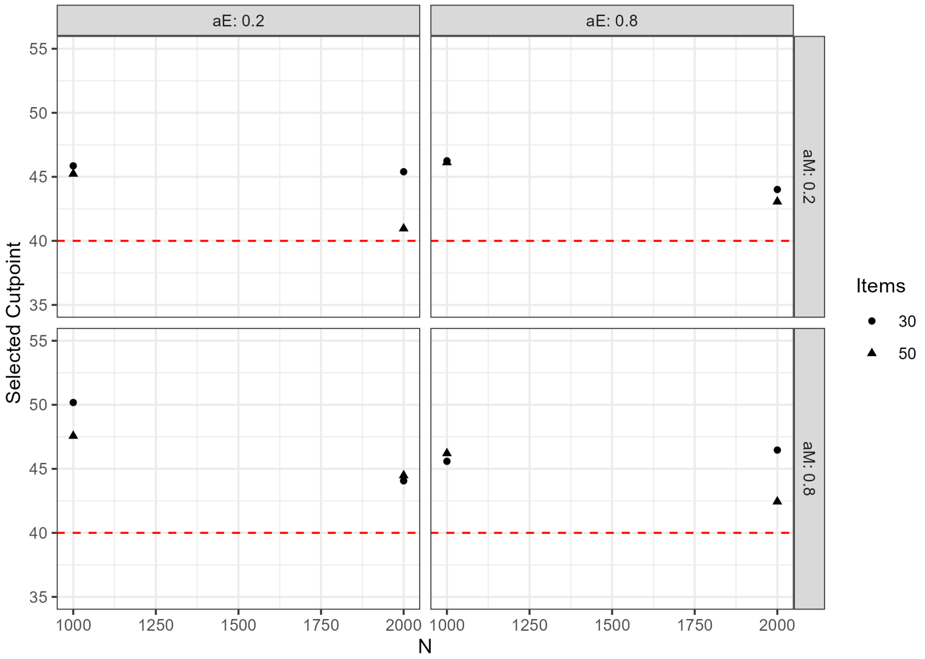

Change of Extreme Response Parameter by +0.5

Figure 5

The mean proposed cutpoint under different conditions of sample size, test length and factor loadings when α^e was affected by a parameter change of +0.5. The red line denotes the true cutpoint of 40.

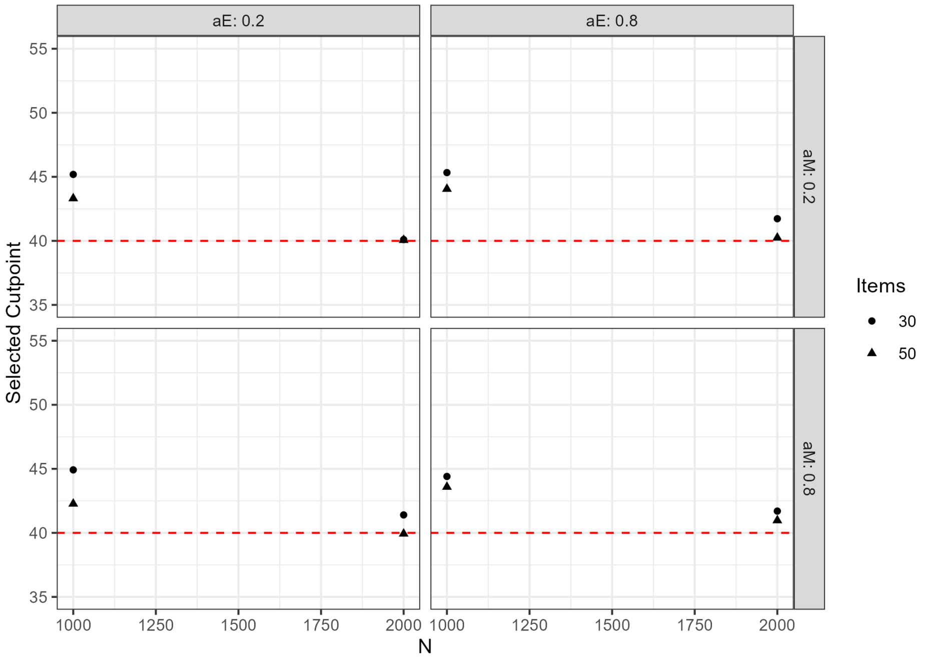

Change of Extreme Response Parameter by +0.2

Figure 6

The mean proposed cutpoint under different conditions of sample size, test length and factor loadings when α^e was affected by a parameter change of +0.2. The red line denotes the true cutpoint of 40.

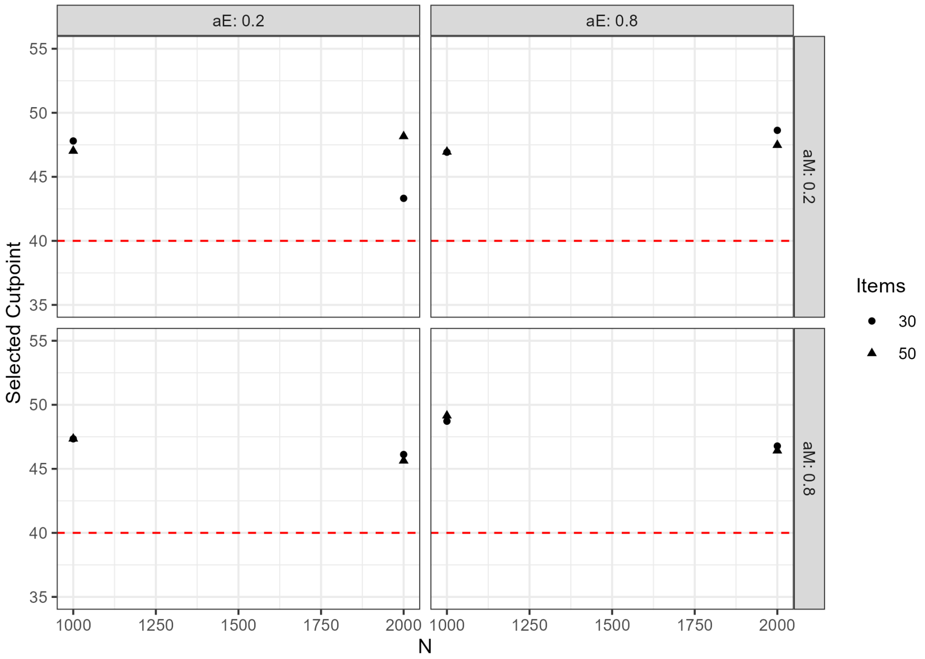

Change of Extreme Response Parameter by -0.5

Figure 7

The mean proposed cutpoint under different conditions of sample size, test length and factor loadings when α^e was affected by a parameter change of -0.5. The red line denotes the true cutpoint of 40.

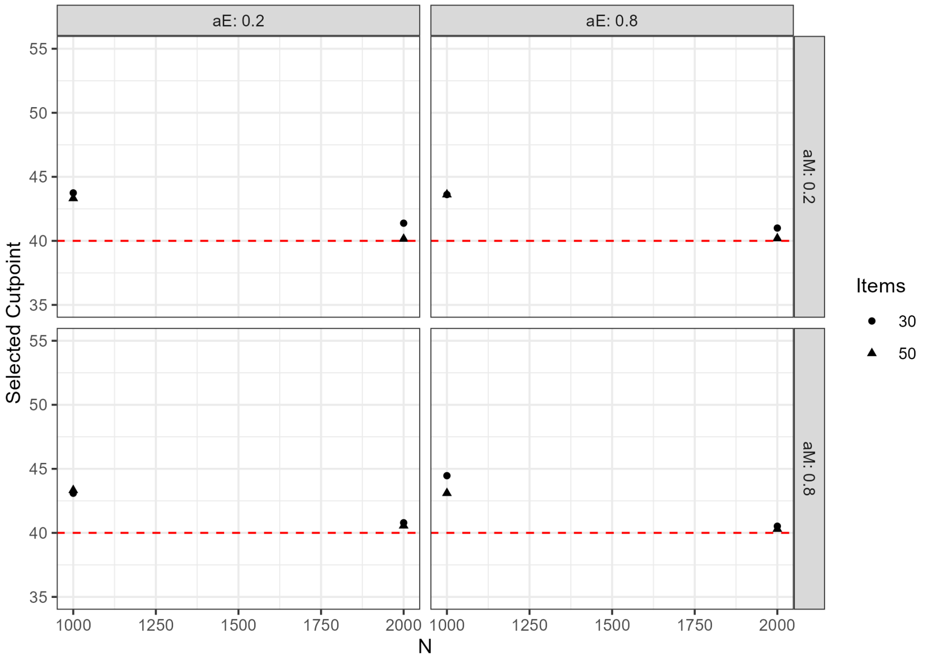

Change of Extreme Response Parameter by -0.2

Figure 8

The mean proposed cutpoint under different conditions of sample size, test length and factor loadings when α^e was affected by a parameter change of -0.2. The red line denotes the true cutpoint of 40.

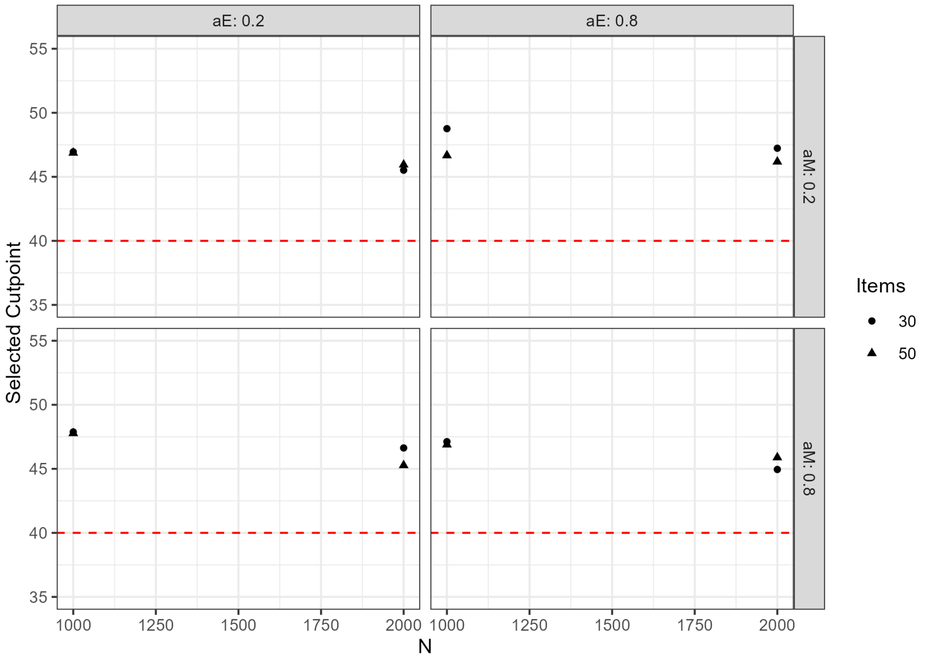

Ordinal Covariate, Test Statistic `WDMo`, Symmetrical Distribution of Covariate

Change of Non-Moderate Response Parameter by +0.5

Figure 9

The mean proposed cutpoint under different conditions of sample size, test length and factor loadings when α^{nm} was affected by a parameter change of +0.5. The red line denotes the true cutpoint of 3.

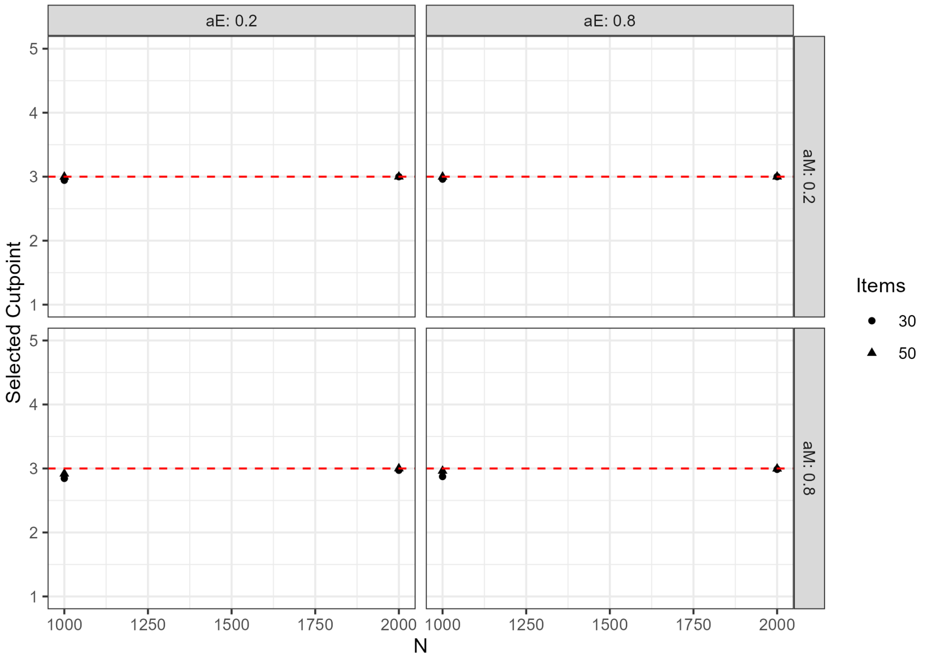

Change of Non-Moderate Response Parameter by +0.2

Figure 10

The mean proposed cutpoint under different conditions of sample size, test length and factor loadings when α^{nm} was affected by a parameter change of +0.2. The red line denotes the true cutpoint of 3.

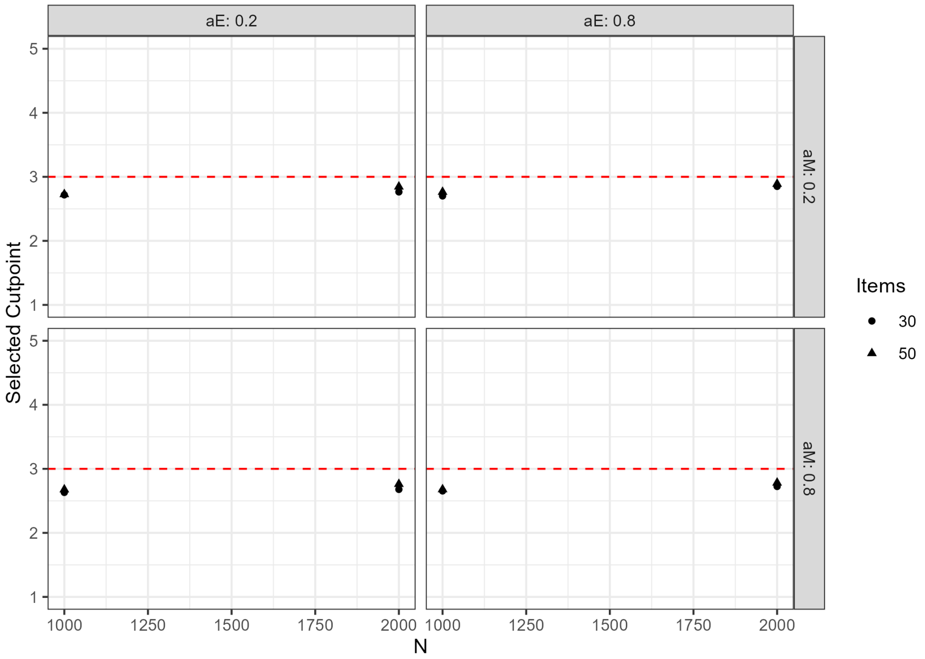

Change of Non-Moderate Response Parameter by -0.5

Figure 11

The mean proposed cutpoint under different conditions of sample size, test length and factor loadings when α^{nm} was affected by a parameter change of -0.5. The red line denotes the true cutpoint of 3.

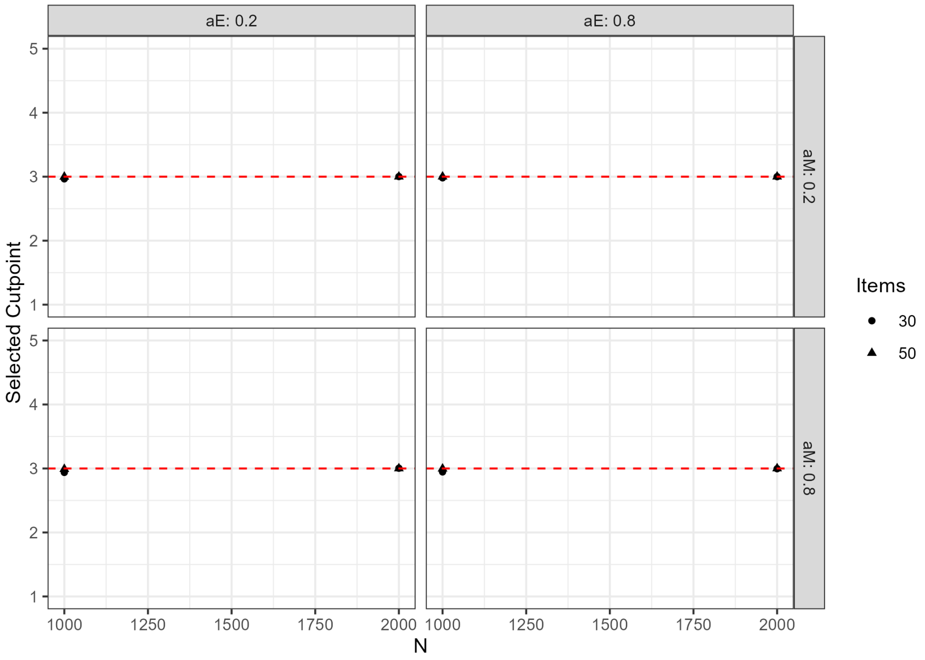

Change of Non-Moderate Response Parameter by -0.2

Figure 12

The mean proposed cutpoint under different conditions of sample size, test length and factor loadings when α^{nm} was affected by a parameter change of -0.2. The red line denotes the true cutpoint of 3.

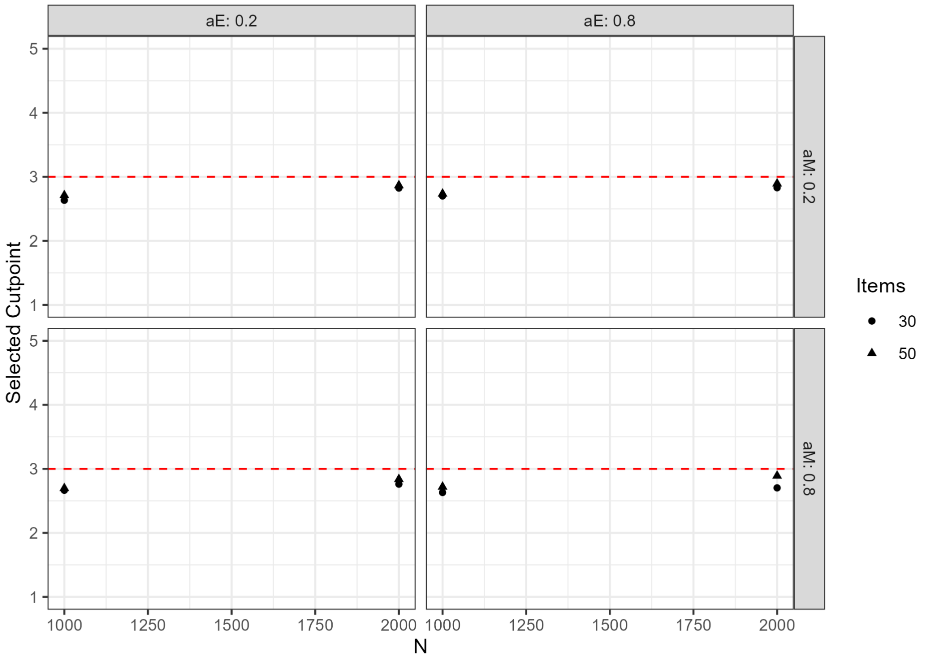

Change of Extreme Response Parameter by +0.5

Figure 13

The mean proposed cutpoint under different conditions of sample size, test length and factor loadings when α^e was affected by a parameter change of +0.5. The red line denotes the true cutpoint of 3.

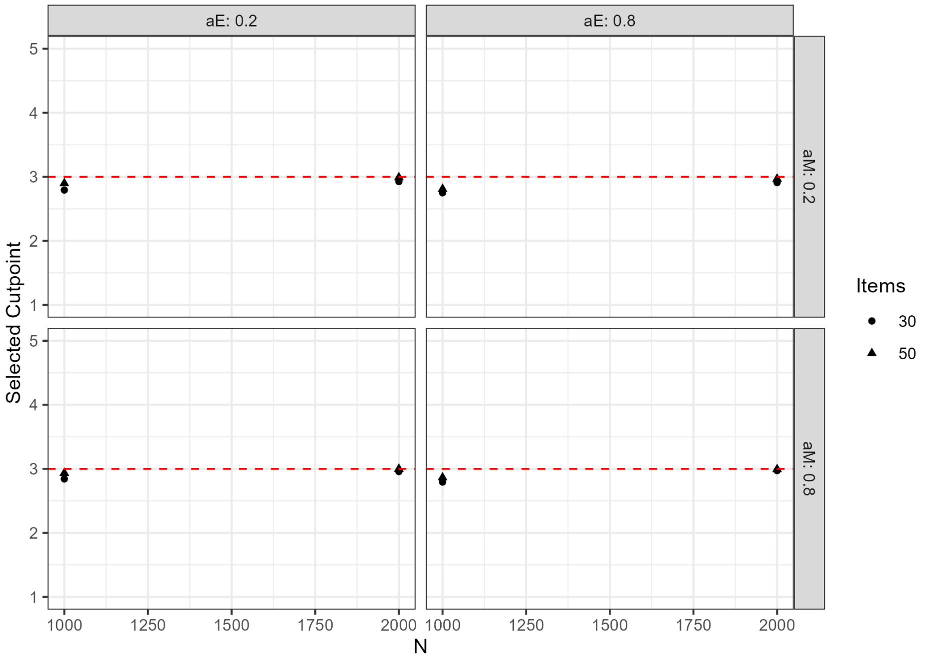

Change of Extreme Response Parameter by +0.2

Figure 14

The mean proposed cutpoint under different conditions of sample size, test length and factor loadings when α^e was affected by a parameter change of +0.2. The red line denotes the true cutpoint of 3.

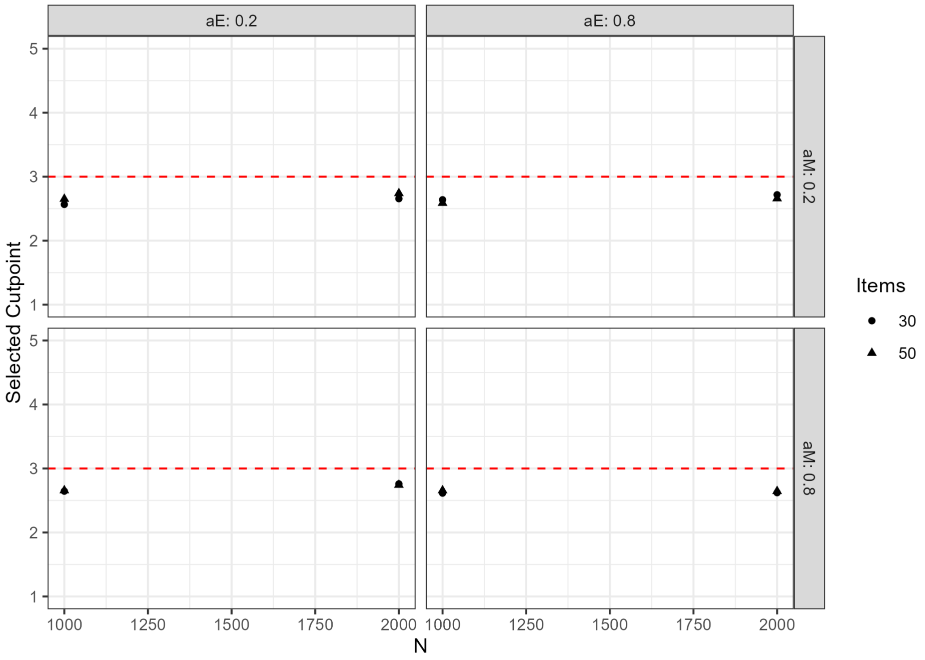

Change of Extreme Response Parameter by -0.5

Figure 15

The mean proposed cutpoint under different conditions of sample size, test length and factor loadings when α^e was affected by a parameter change of -0.5. The red line denotes the true cutpoint of 3.

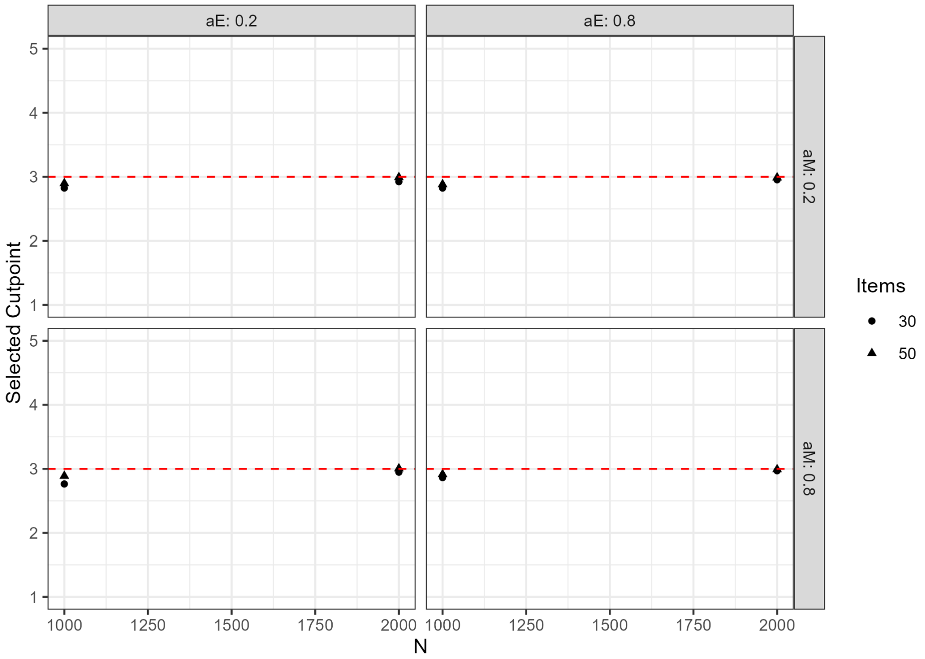

Change of Extreme Response Parameter by -0.2

Figure 16

The mean proposed cutpoint under different conditions of sample size, test length and factor loadings when α^e was affected by a parameter change of -0.2. The red line denotes the true cutpoint of 3.

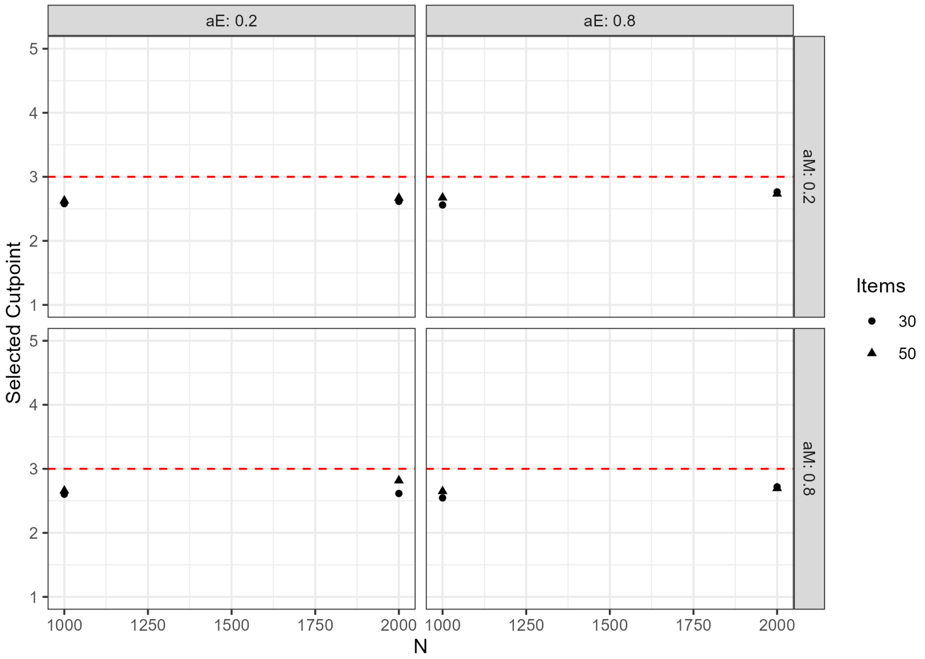

Ordinal Covariate, Test Statistic WDMo, Skewed Distribution of Covariate

Change of Non-Moderate Response Parameter by +0.5

Figure 17

The mean proposed cutpoint under different conditions of sample size, test length and factor loadings when α^{nm} was affected by a parameter change of +0.5. The red line denotes the true cutpoint of 3.

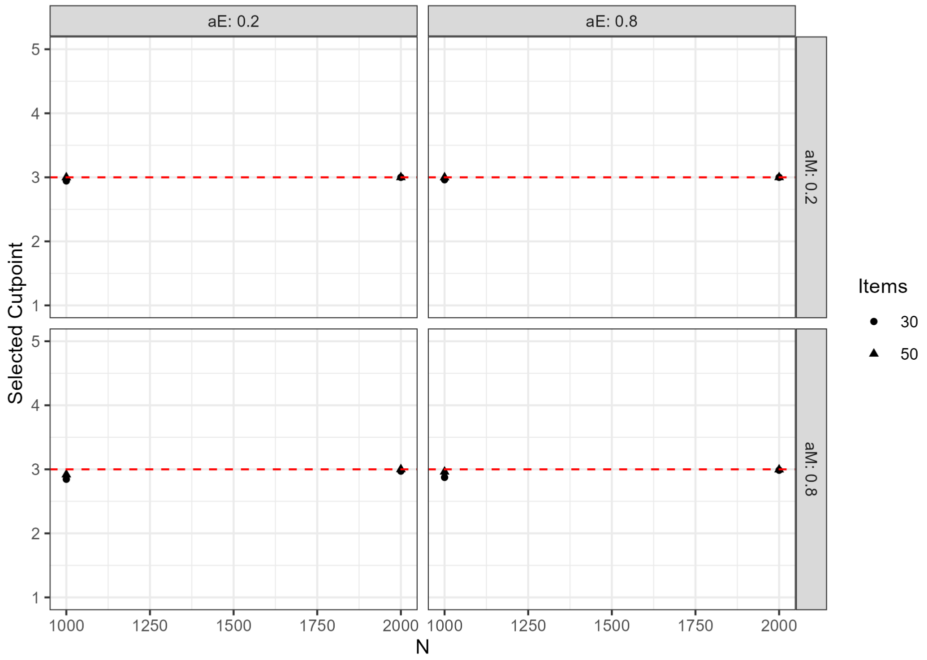

Change of Non-Moderate Response Parameter by +0.2

Figure 18

The mean proposed cutpoint under different conditions of sample size, test length and factor loadings when α^{nm} *was affected by a parameter change of +0.2. The red line denotes the true cutpoint of 3.*

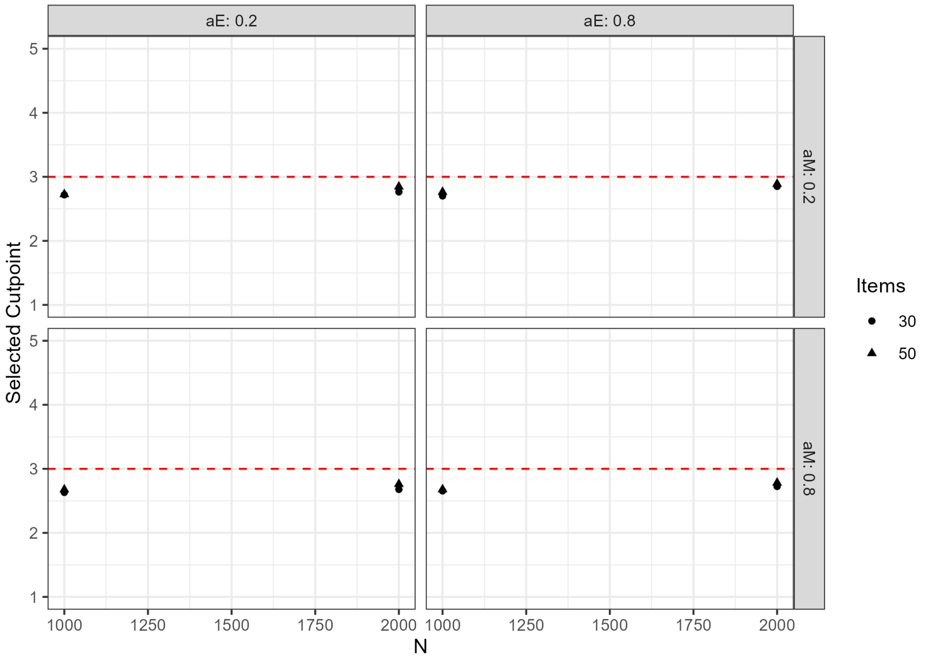

Change of Non-Moderate Response Parameter by -0.5

Figure 19

The mean proposed cutpoint under different conditions of sample size, test length and factor loadings when α^{nm} was affected by a parameter change of -0.5. The red line denotes the true cutpoint of 3.

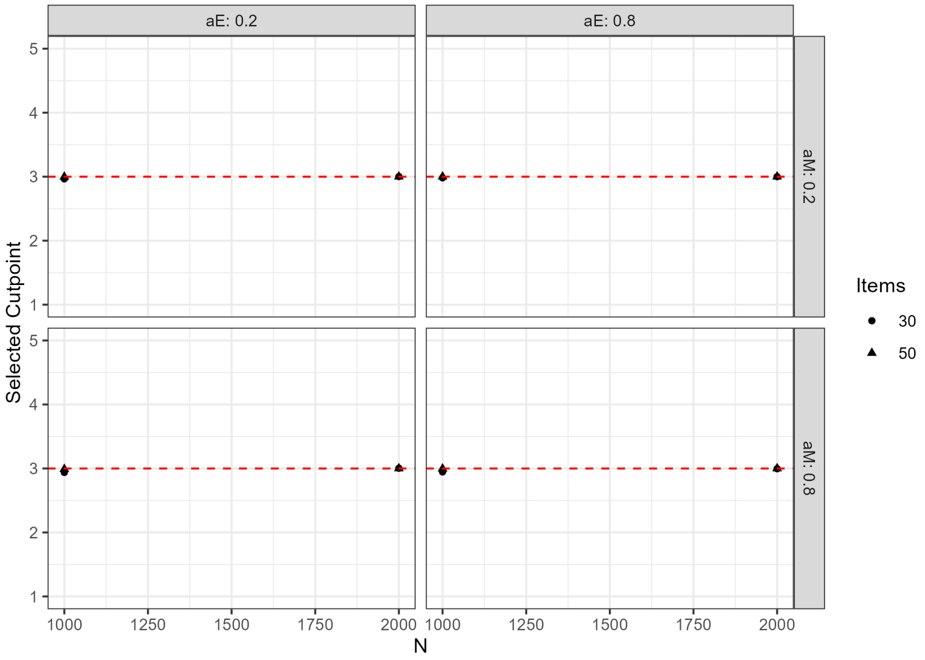

Change of Non-Moderate Response Parameter by -0.2

Figure 20

The mean proposed cutpoint under different conditions of sample size, test length and factor loadings when α^{nm} was affected by a parameter change of -0.2. The red line denotes the true cutpoint of 3.

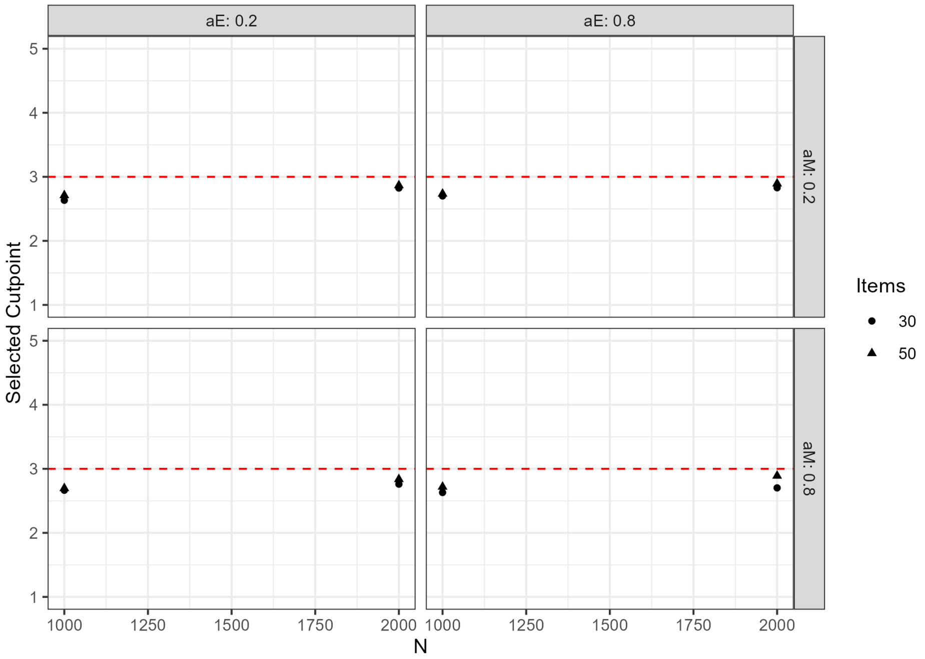

Change of Extreme Response Parameter by +0.5

Figure 21

The mean proposed cutpoint under different conditions of sample size, test length and factor loadings when α^e was affected by a parameter change of +0.5. The red line denotes the true cutpoint of 3.

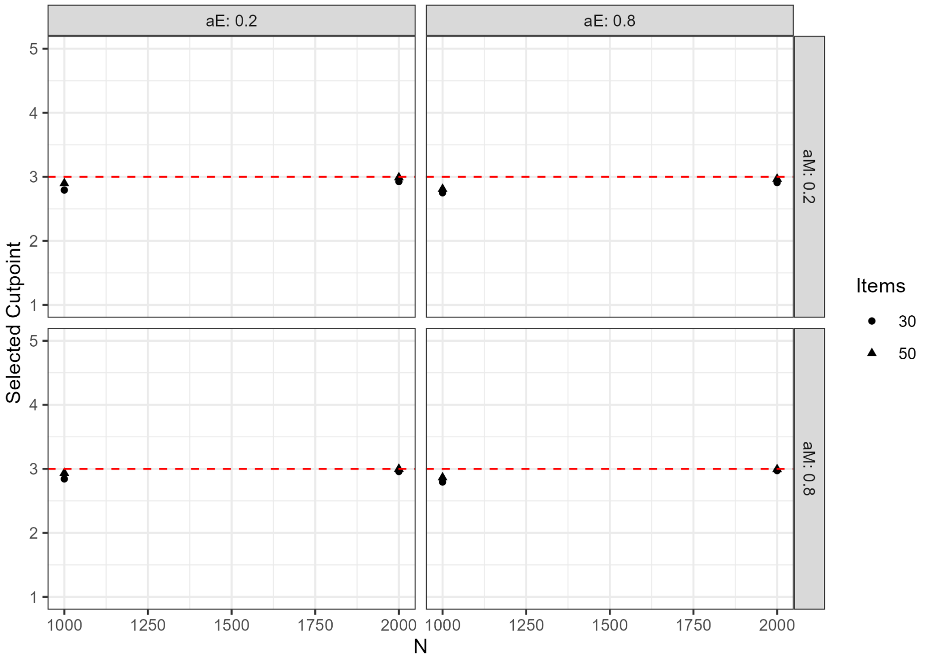

Change of Extreme Response Parameter by +0.2

Figure 22

The mean proposed cutpoint under different conditions of sample size, test length and factor loadings when α^e was affected by a parameter change of +0.2. The red line denotes the true cutpoint of 3.

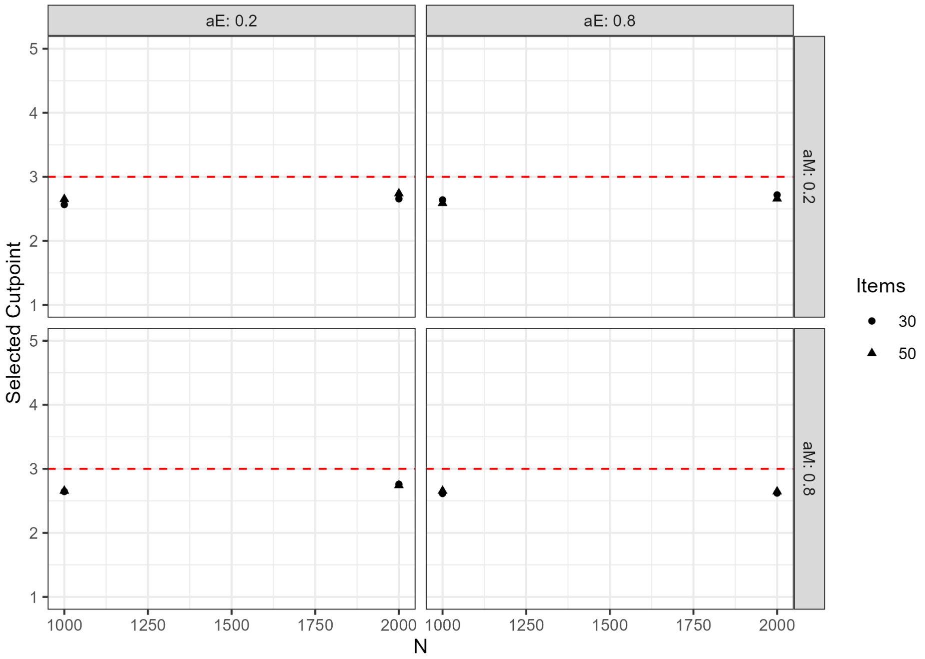

Change of Extreme Response Parameter by -0.5

Figure 23

The mean proposed cutpoint under different conditions of sample size, test length and factor loadings when α^e was affected by a parameter change of -0.5. The red line denotes the true cutpoint of 3.

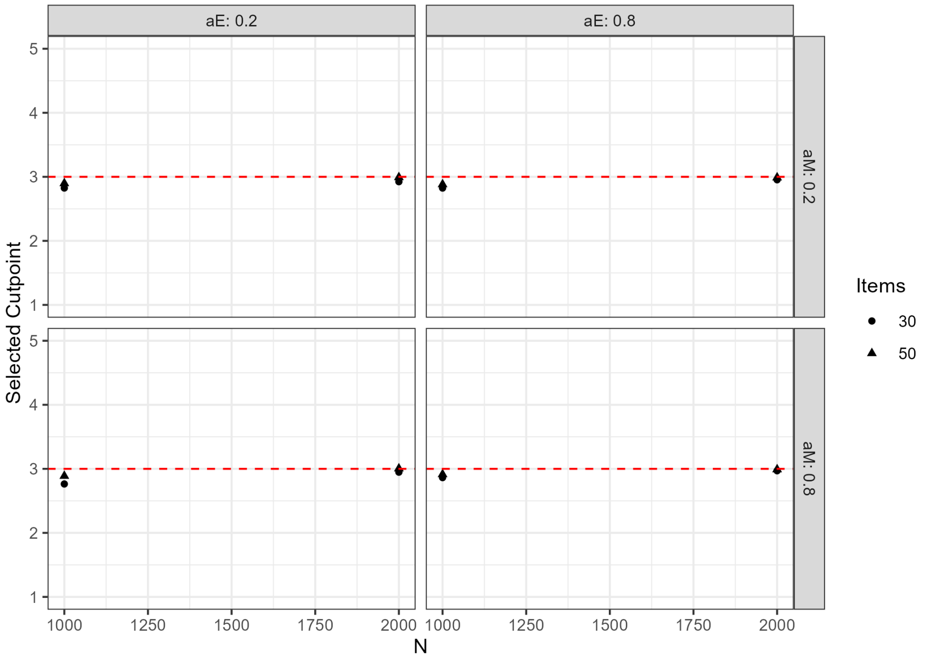

Change of Extreme Response Parameter by -0.2

Figure 24

The mean proposed cutpoint under different conditions of sample size, test length and factor loadings when α^e was affected by a parameter change of -0.2. The red line denotes the true cutpoint of 3.

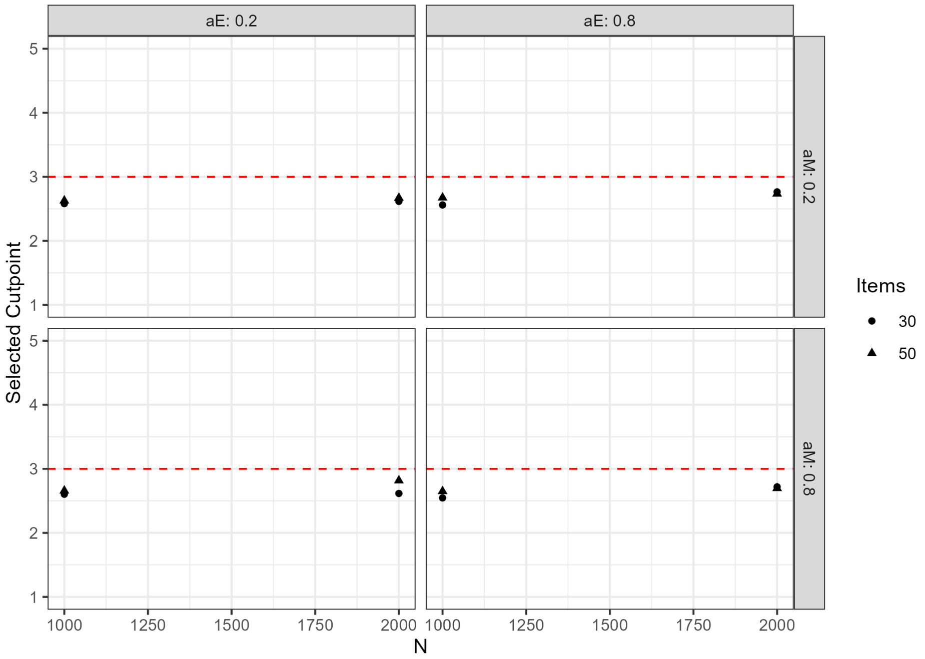

Ordinal Covariate, Test Statistic `maxLMo`, Symmetrical Distribution of Covariate

Change of Non-Moderate Response Parameter by +0.5

Figure 25

The mean proposed cutpoint under different conditions of sample size, test length and factor loadings when α^{nm} was affected by a parameter change of +0.5. The red line denotes the true cutpoint of 3.

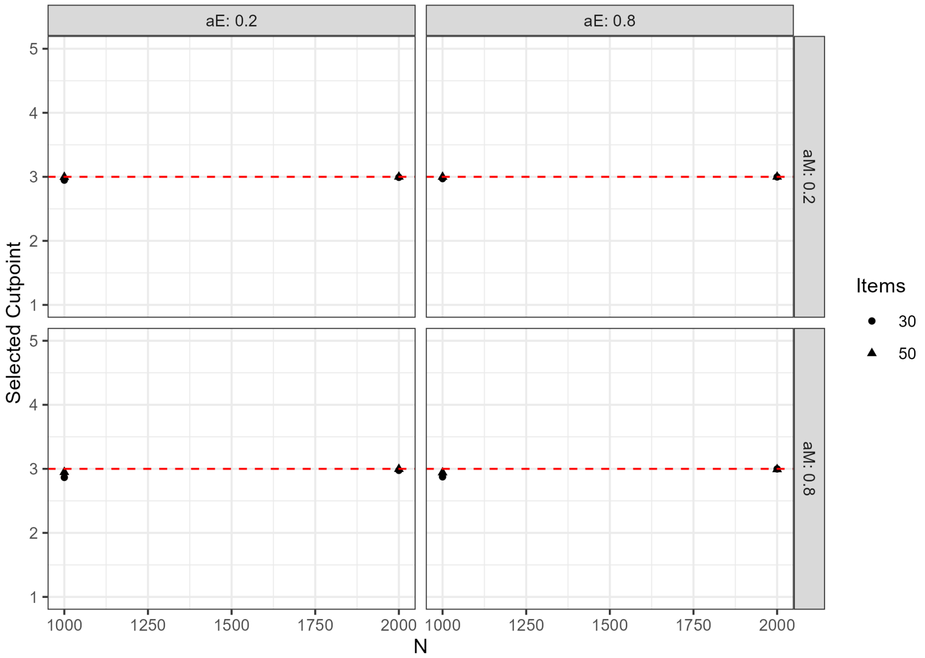

Change of Non-Moderate Response Parameter by +0.2

Figure 26

The mean proposed cutpoint under different conditions of sample size, test length and factor loadings when α^{nm} was affected by a parameter change of +0.2. The red line denotes the true cutpoint of 3.

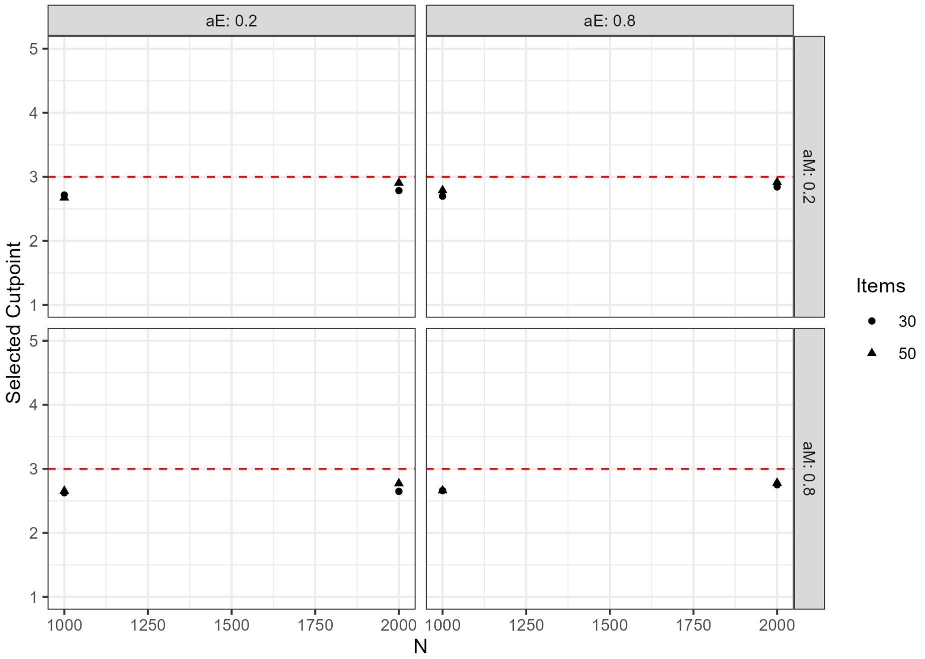

Change of Non-Moderate Response Parameter by +0.5

Figure 27

The mean proposed cutpoint under different conditions of sample size, test length and factor loadings when α^{nm} *was affected by a parameter change of +0.5. The red line denotes the true cutpoint of 3.*

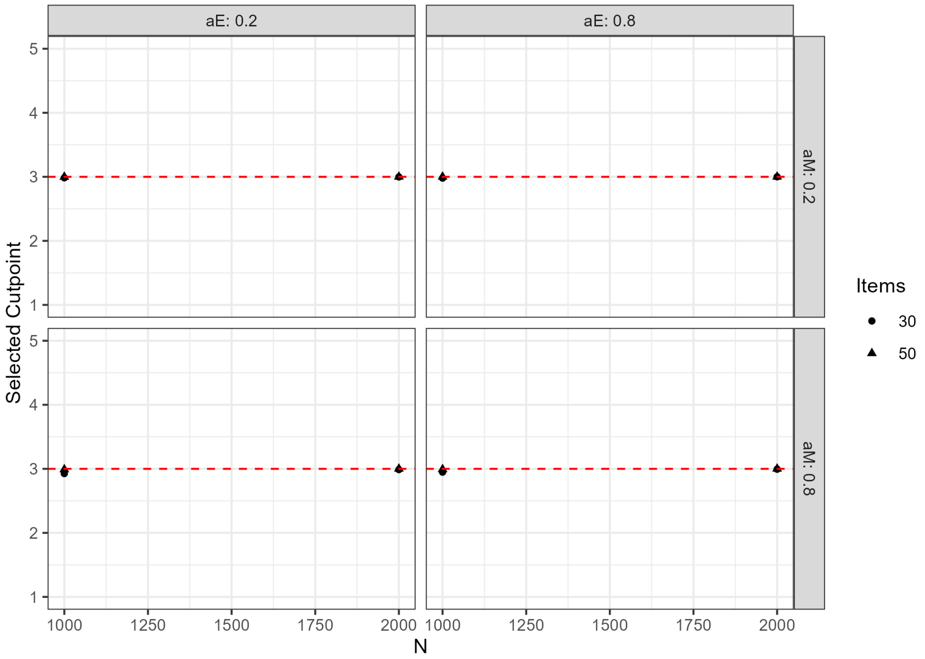

Change of Non-Moderate Response Parameter by -0.2

Figure 28

The mean proposed cutpoint under different conditions of sample size, test length and factor loadings when α^{nm} was affected by a parameter change of -0.2. The red line denotes the true cutpoint of 3.

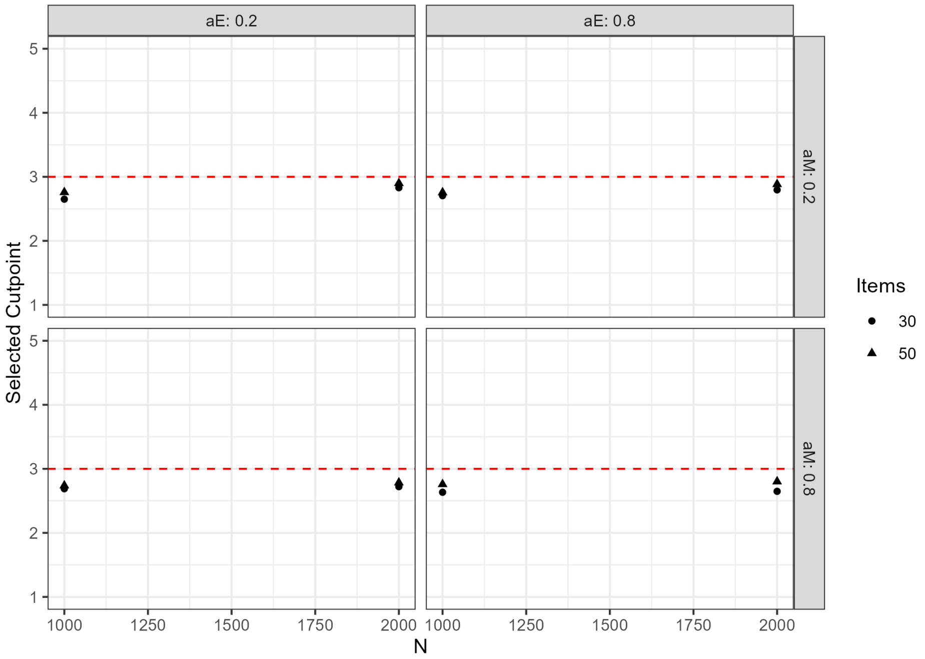

Change of Extreme Response Parameter by +0.5

Figure 29

The mean proposed cutpoint under different conditions of sample size, test length and factor loadings when α^e was affected by a parameter change of +0.5. The red line denotes the true cutpoint of 3.

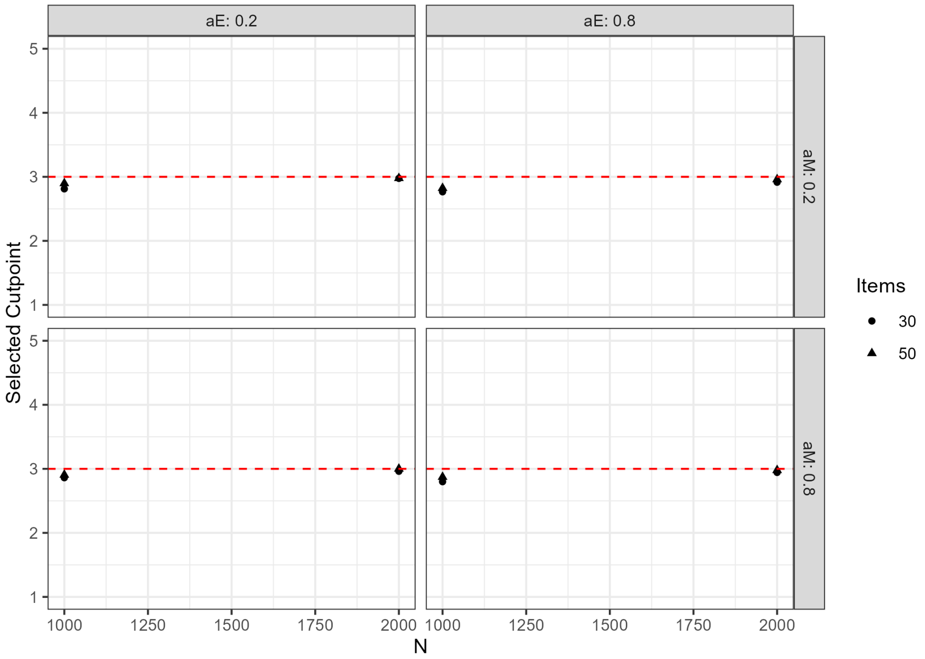

Change of Extreme Response Parameter by +0.2

Figure 30

The mean proposed cutpoint under different conditions of sample size, test length and factor loadings when α^e was affected by a parameter change of +0.2. The red line denotes the true cutpoint of 3.

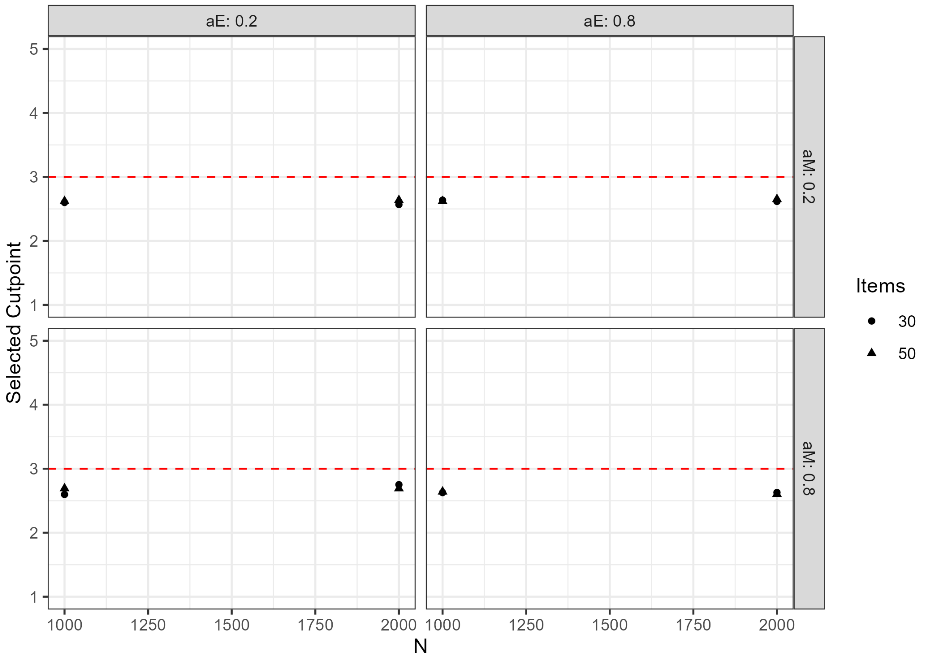

Change of Extreme Response Parameter by -0.5

Figure 31

The mean proposed cutpoint under different conditions of sample size, test length and factor loadings when α^e was affected by a parameter change of -0.5. The red line denotes the true cutpoint of 3.

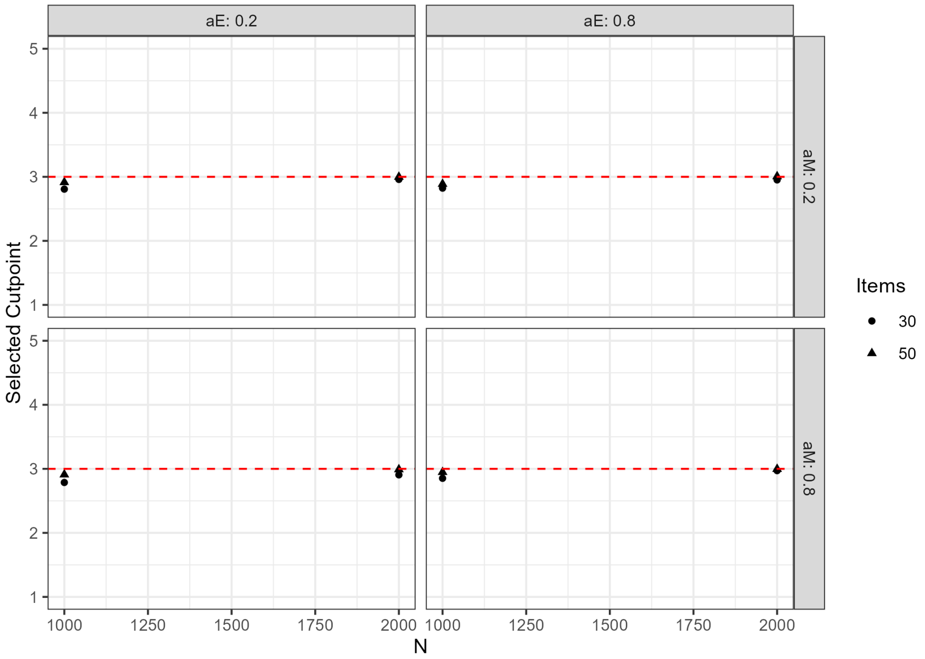

Change of Extreme Response Parameter by -0.2

Figure 32

The mean proposed cutpoint under different conditions of sample size, test length and factor loadings when α^e was affected by a parameter change of -0.2. The red line denotes the true cutpoint of 3.

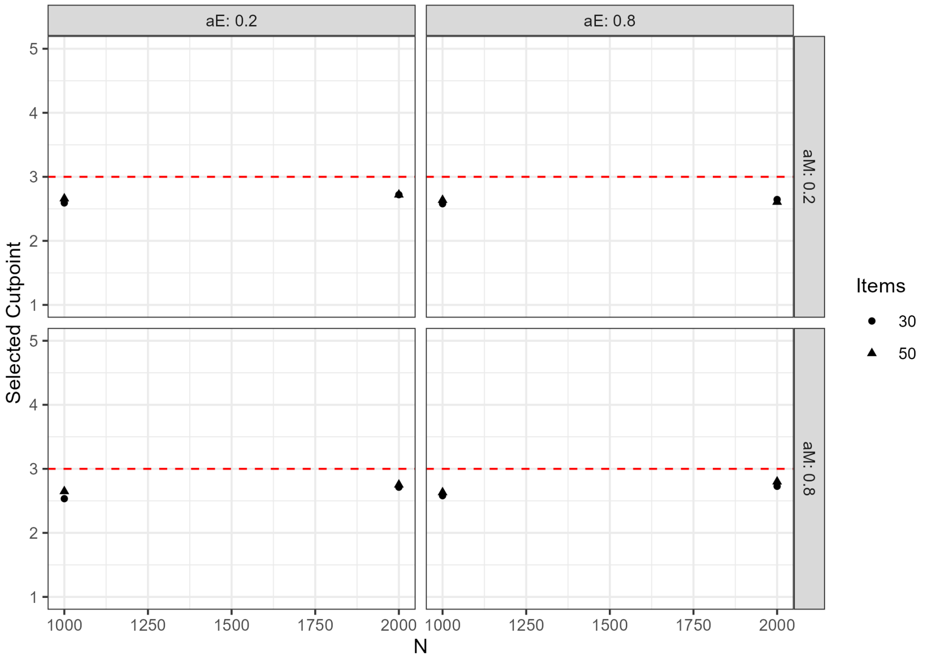

Ordinal Covariate, Test Statistic `maxLMo`, Skewed Distribution of Covariate

Change of Non-Moderate Response Parameter by +0.5

Figure 33

The mean proposed cutpoint under different conditions of sample size, test length and factor loadings when α^{nm} was affected by a parameter change of +0.5. The red line denotes the true cutpoint of 3.

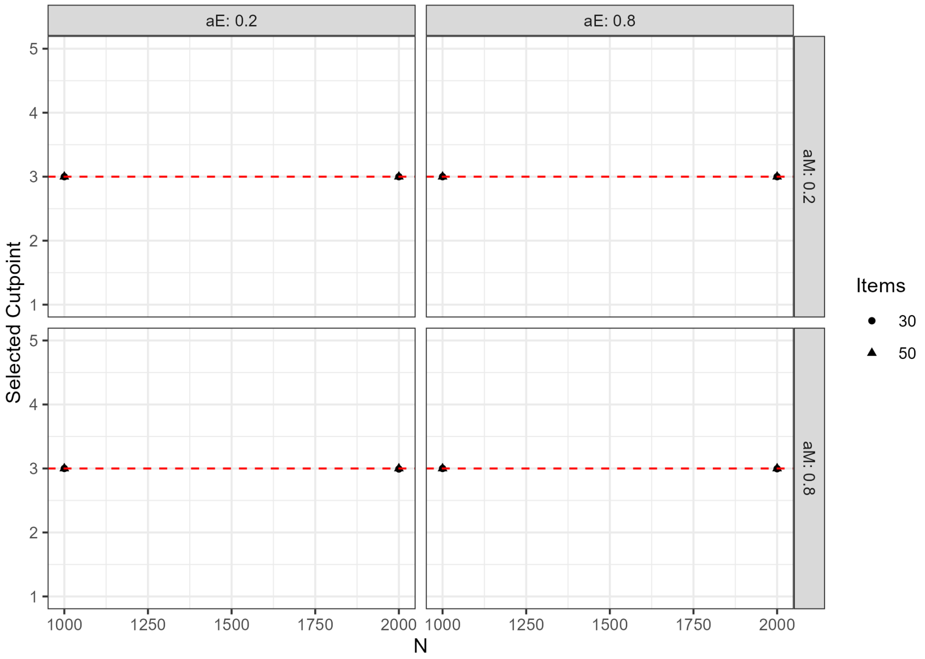

Change of Non-Moderate Response Parameter by +0.2

Figure 34

The mean proposed cutpoint under different conditions of sample size, test length and factor loadings when α^{nm} *was affected by a parameter change of +0.2. The red line denotes the true cutpoint of 3.*

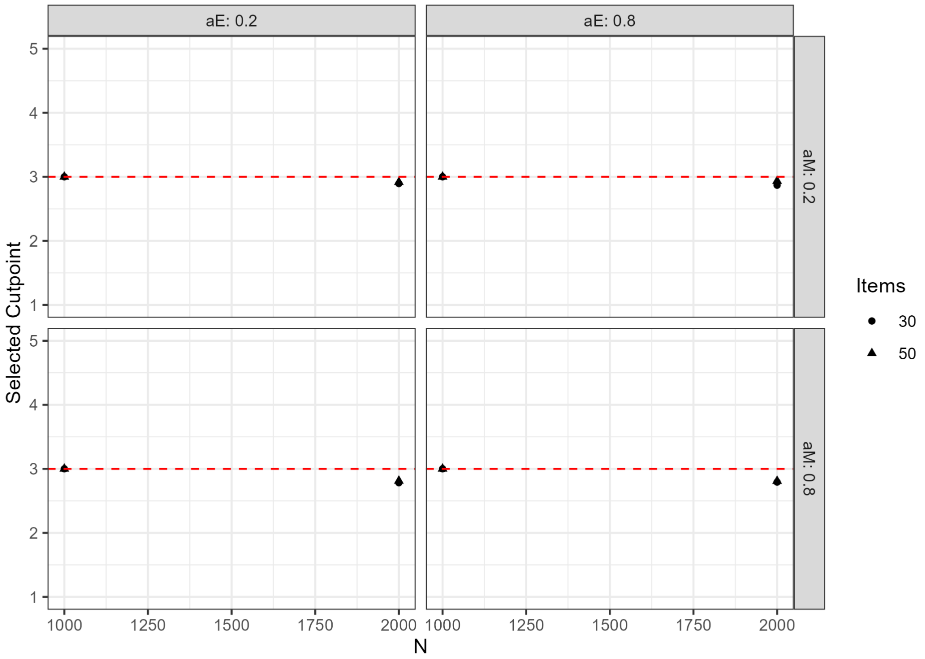

Change of Non-Moderate Response Parameter by -0.5

Figure 35

The mean proposed cutpoint under different conditions of sample size, test length and factor loadings when α^{nm} was affected by a parameter change of -0.5. The red line denotes the true cutpoint of 3.

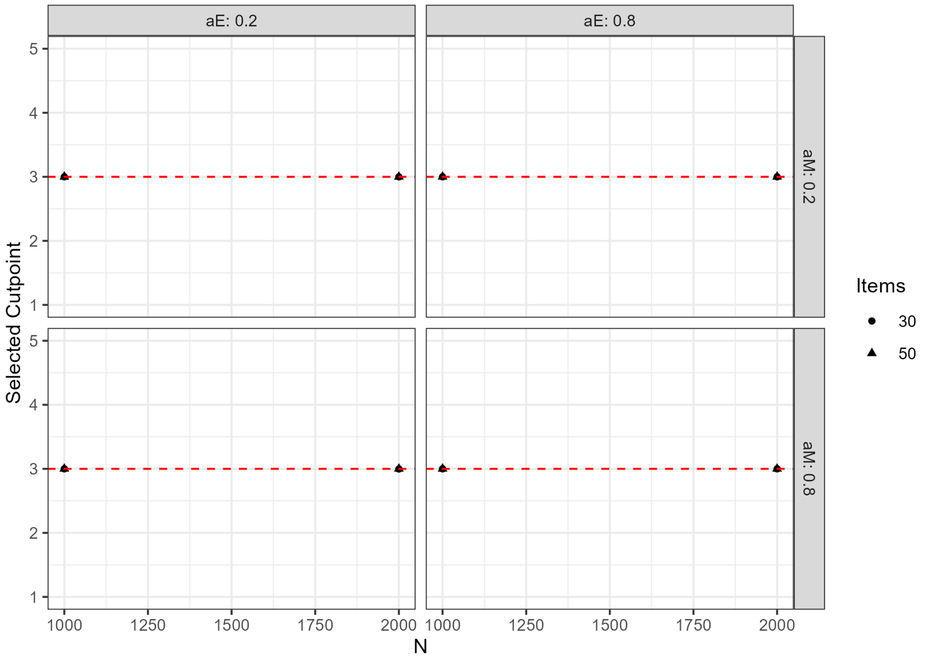

Change of Non-Moderate Response Parameter by -0.2

Figure 36

The mean proposed cutpoint under different conditions of sample size, test length and factor loadings when α^{nm} was affected by a parameter change of -0.2. The red line denotes the true cutpoint of 3.

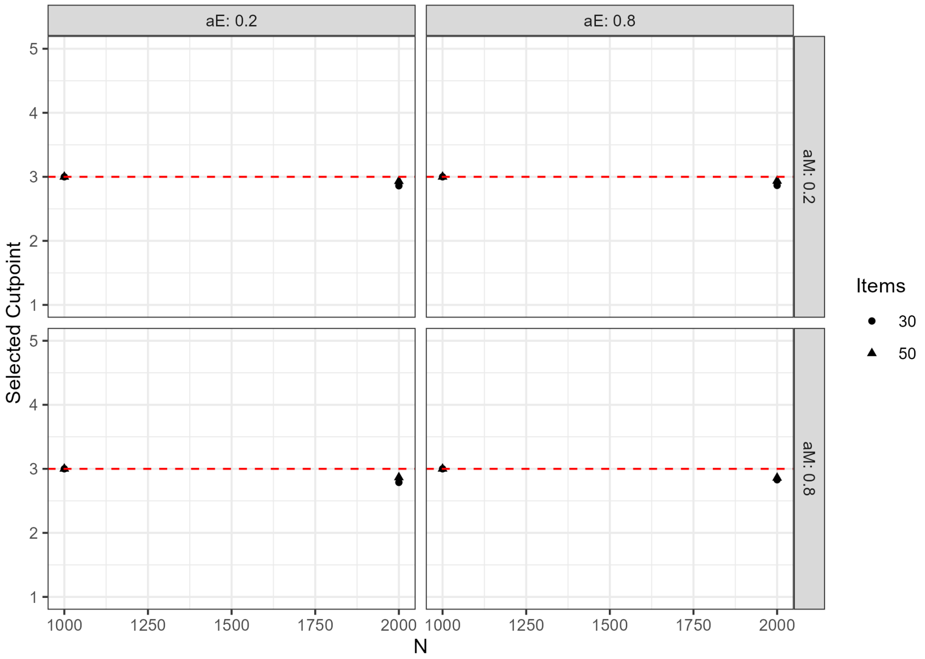

Change of Extreme Response Parameter by +0.5

Figure 37

The mean proposed cutpoint under different conditions of sample size, test length and factor loadings when α^e was affected by a parameter change of +0.5. The red line denotes the true cutpoint of 3.

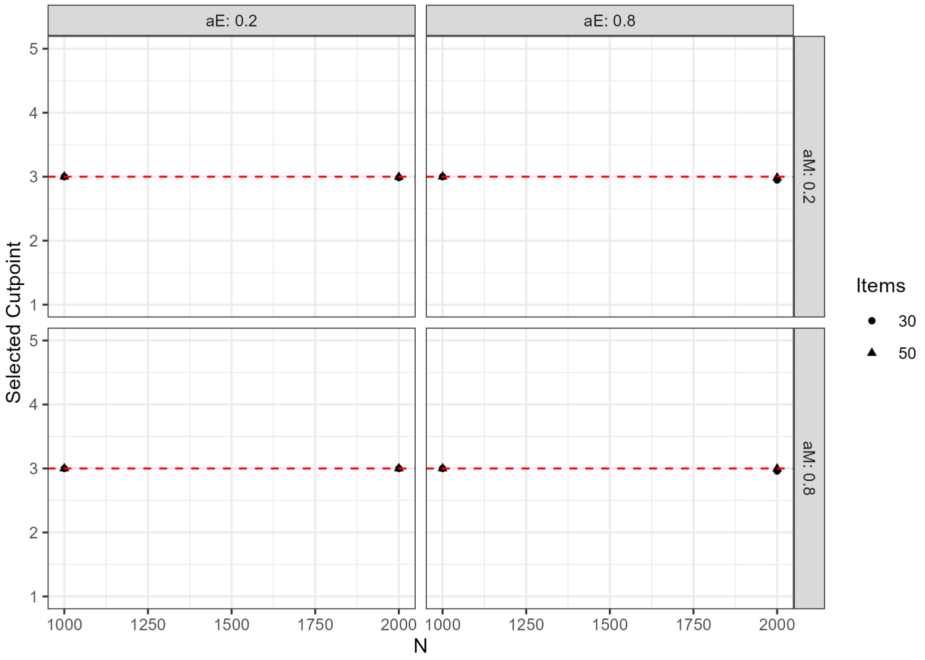

Change of Extreme Response Parameter by +0.2

Figure 38

The mean proposed cutpoint under different conditions of sample size, test length and factor loadings when α^e was affected by a parameter change of +0.2. The red line denotes the true cutpoint of 3.

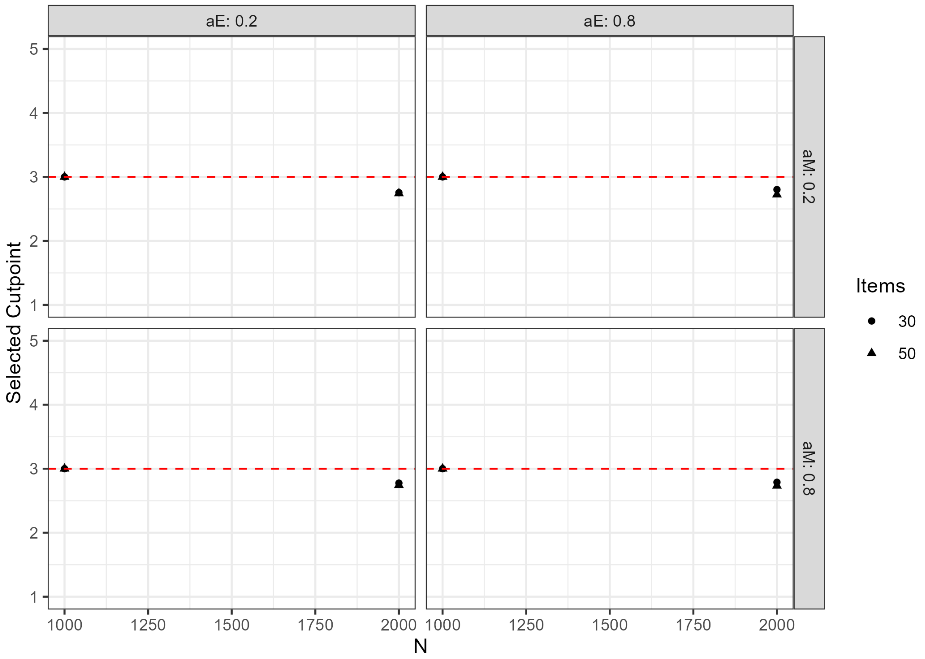

Change of Extreme Response Parameter by -0.5

Figure 39

The mean proposed cutpoint under different conditions of sample size, test length and factor loadings when α^e was affected by a parameter change of -0.5. The red line denotes the true cutpoint of 3.

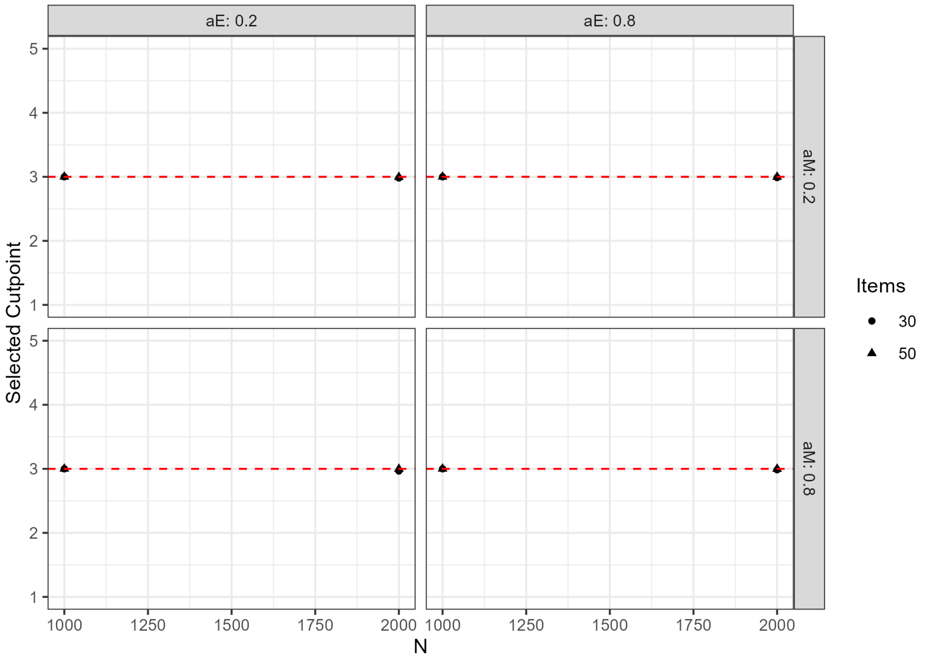

Change of Extreme Response Parameter by -0.2

Figure 40

The mean proposed cutpoint under different conditions of sample size, test length and factor loadings when α^e was affected by a parameter change of -0.2. The red line denotes the true cutpoint of 3.

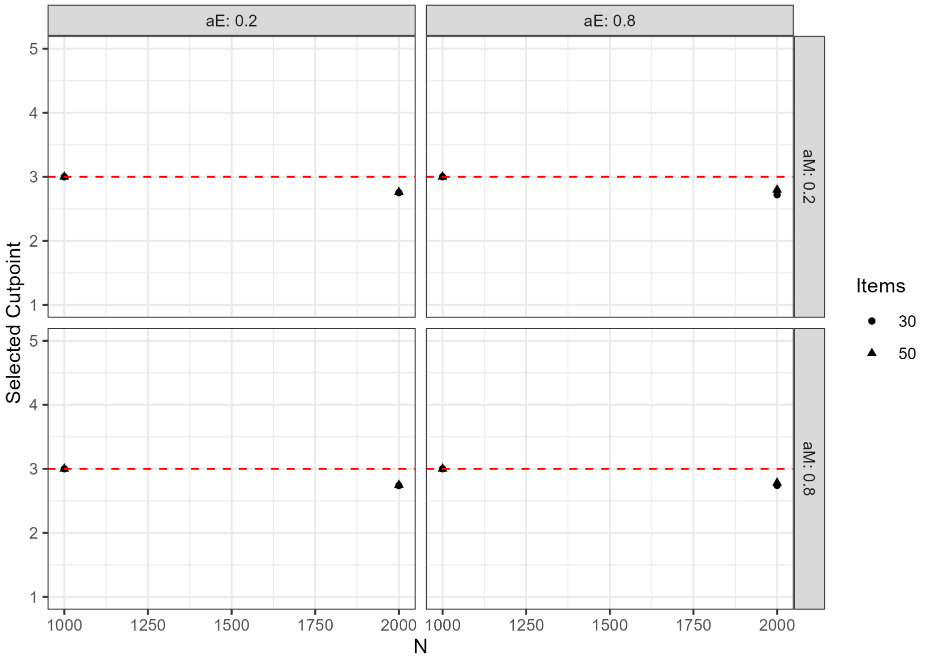

Supplement: Supplementary file 2 — Data S2. [file BMSP-78-420-s002.pdf]
